# Supplementary material for: The CHK1 inhibitor prexasertib in BRCA wild-type platinum-resistant recurrent high-grade serous ovarian carcinoma: a phase 2 trial
Source: Nat Commun. 2024 Mar 30;15:2805. doi: 10.1038/s41467-024-47215-6 (PMC10981752; doi:10.1038/s41467-024-47215-6)
Supplement: Supplementary file 1 — Supplementary Information [file 41467_2024_47215_MOESM1_ESM.pdf]

Supplementary Information

The CHK1 inhibitor prexasertib in *BRCA* wild-type platinum-resistant recurrent high-grade serous ovarian carcinoma: a phase 2 trial

Elena Giudice[1,2,#], Tzu-Ting Huang[1,#], Jayakumar R. Nair[1,#], Grant Zurcher[1], Ann McCoy[1], Darryl Nousome[3], Marc R. Radke[4], Elizabeth M. Swisher[4], Stanley Lipkowitz[1], Kristen Ibanez[1], Duncan Donohue[5], Tyler Malys[5], Min-Jung Lee[6], Bernadette Redd[7], Elliot Levy[8], Shraddha Rastogi[6], Nahoko Sato[6], Jane B. Trepel[6], and Jung-Min Lee[1,*]

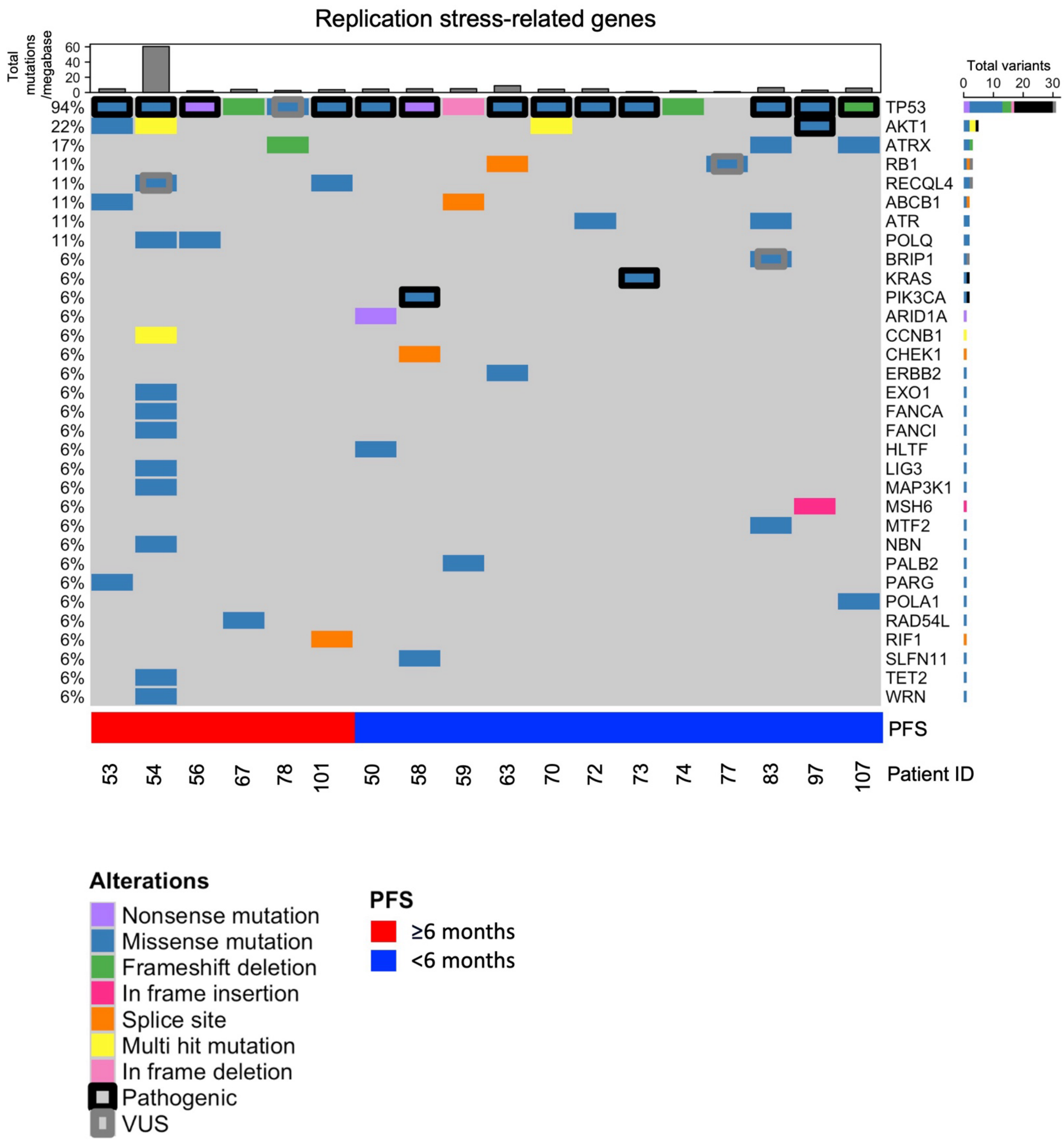

Supplementary Fig. 1: Oncoplot representing distribution of replication stress-associated genes from WES. WES data analysis revealed the genetic alterations of genes related to replication stress (cohort 5, n=18). Source data are provided as a Source Data file.

Abbreviations: PFS, progression-free survival; VUS, variants of uncertain significance; WES, whole exome sequencing.

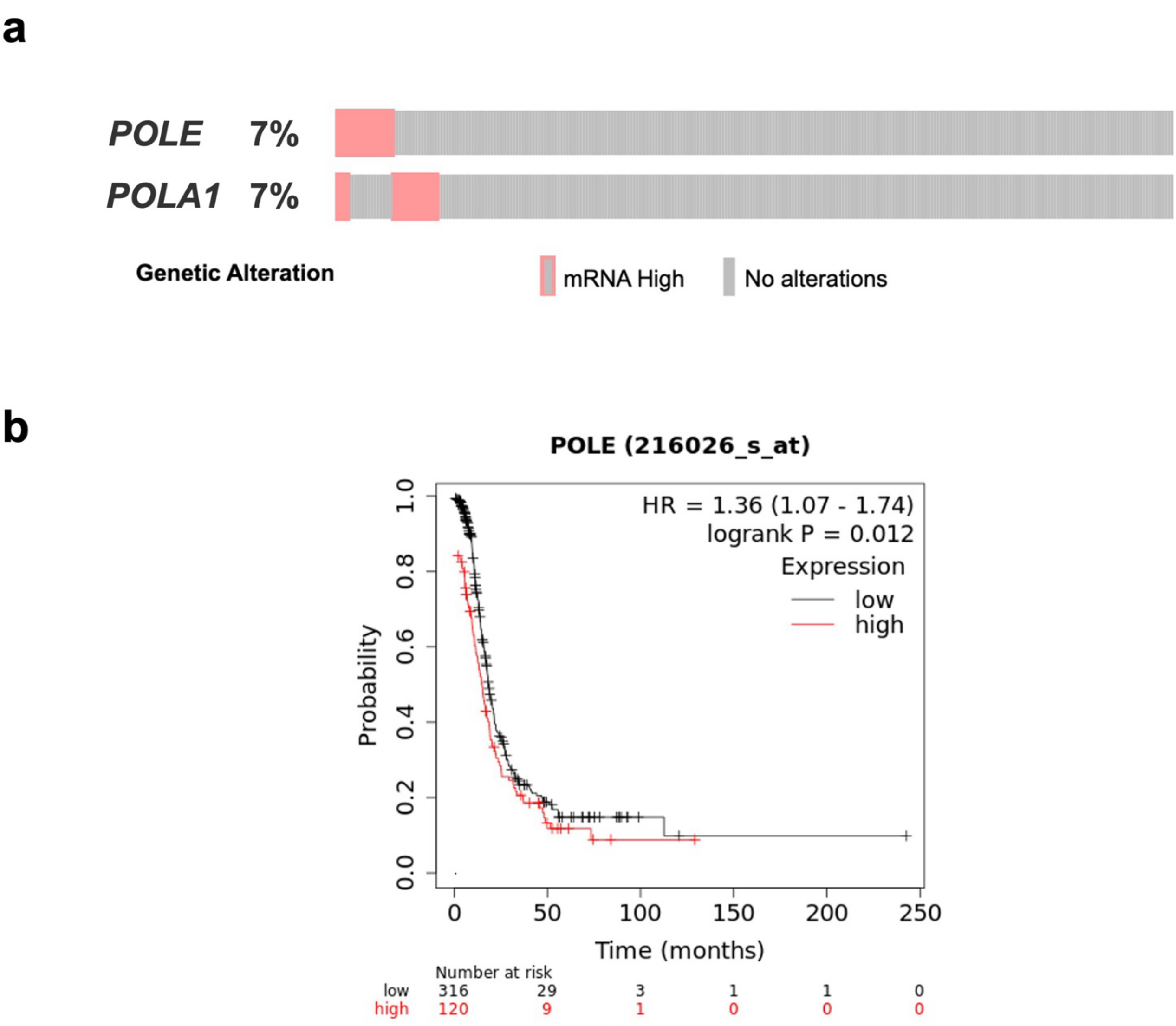

	Low *POLE*	High *POLE*
**Median survival (Months)**	**18.23**	**15.01**

Supplementary Fig. 2: Clinical relevance of *POLE* and *POLA1* in ovarian cancer.

a The alteration of mRNA expression levels of *POLE* and *POLA1* relative to diploid samples (n=300) in ovarian tumors were defined and downloaded from cBioPortal (TCGA, PanCancer Atlas, http://www.cbioportal.org). **b** Prognostic value of *POLE* was obtained from Kaplan–Meier plotter (http://kmplot.com/analysis/) [ovarian cancer] database. Tumors with only serous histology, *TP53* mutation and prior platinum treatment (indicating platinum-treated high-grade serous ovarian cancer) were included for this analysis. Tumors with high (n=120) and low *POLE* levels (n=316) were divided using auto-select best cutoff on the website. The PFS was assessed by a Kaplan-Meier survival plot, with the hazard ratio with 95% confidence intervals and logrank P value. Source data are provided as a Source Data file.

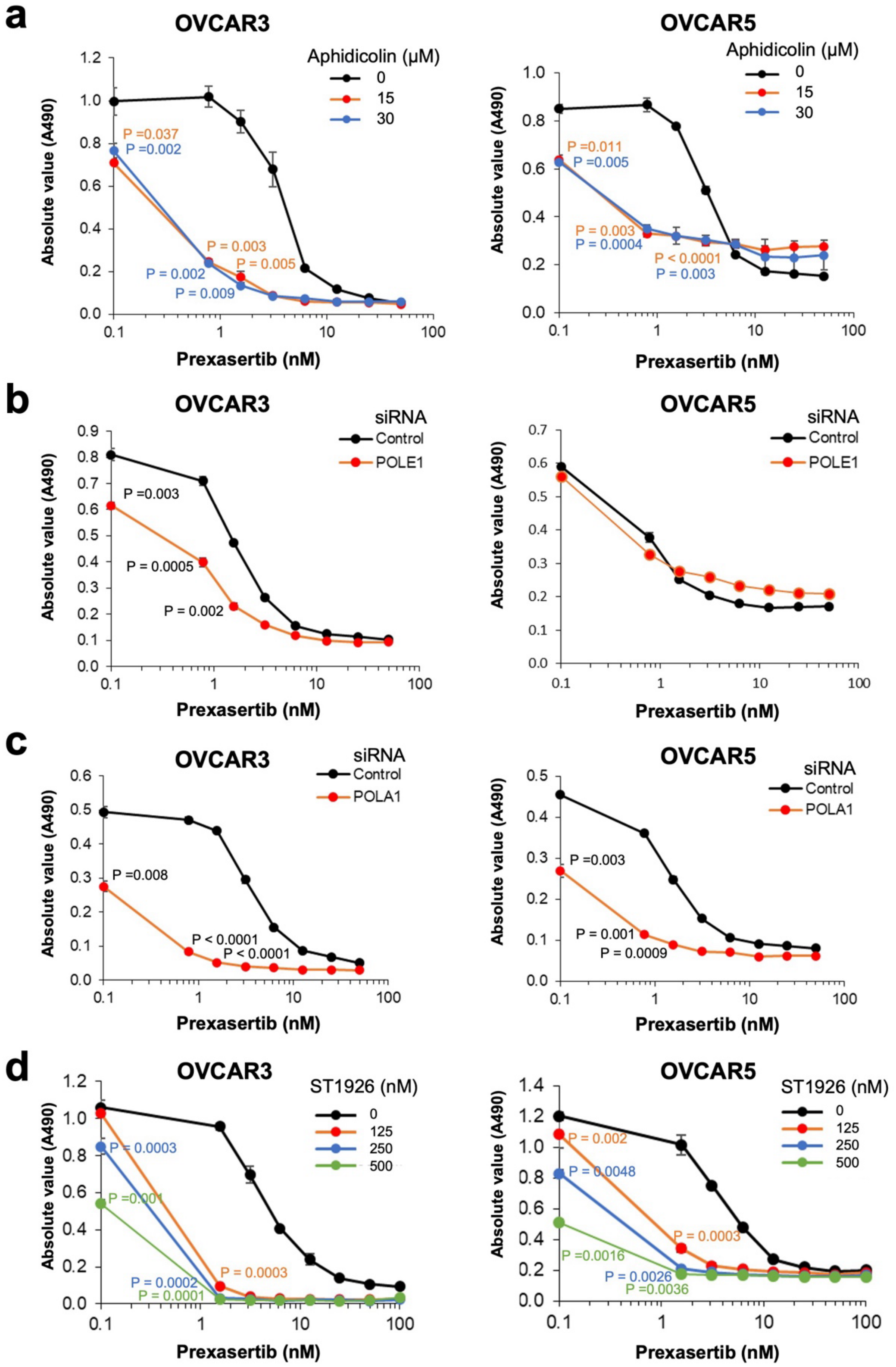

Supplementary Fig. 3: The role of B-family polymerases in CHK1i sensitivity in cisplatin-resistant HGSOC.

a XTT assays (n=3) were performed over 48 hours after treating cells with a gradient of aphidicolin (a pan inhibitor of B-family polymerases) (0-30 μM) alone or in combination with CHK1i prexasertib (0-50 nM). Growth inhibition or survival is plotted as absolute values (A490 nm). **b-c** XTT assays were performed similarly with siRNAs that target either *POLE1* (**b**, n=3) or *POLA1* (**c**, n=3) or a non-targeting control siRNA overnight followed by treatment with serial concentrations of prexasertib (0-50 nM) for 48 hours. **d** XTT assays (n=3) with a POLA1 specific inhibitor ST1926 (0-500 nM) alone or in combination with prexasertib (0-100 nM). Absolute values (A490 nm) are plotted against CHK1i concentrations in OVCAR3 and OVCAR5. Standard Student's *t*-test (two-sided) was used, and data are shown as mean ± SD. Source data are provided as a Source Data file.

Abbreviations: CHK1i, CHK1 inhibitor; HGSOC, high-grade serous ovarian cancer; PrexR, prexasertib-resistant; *POLA1*, DNA polymerase alpha 1; *POLE*, DNA polymerase epsilon.

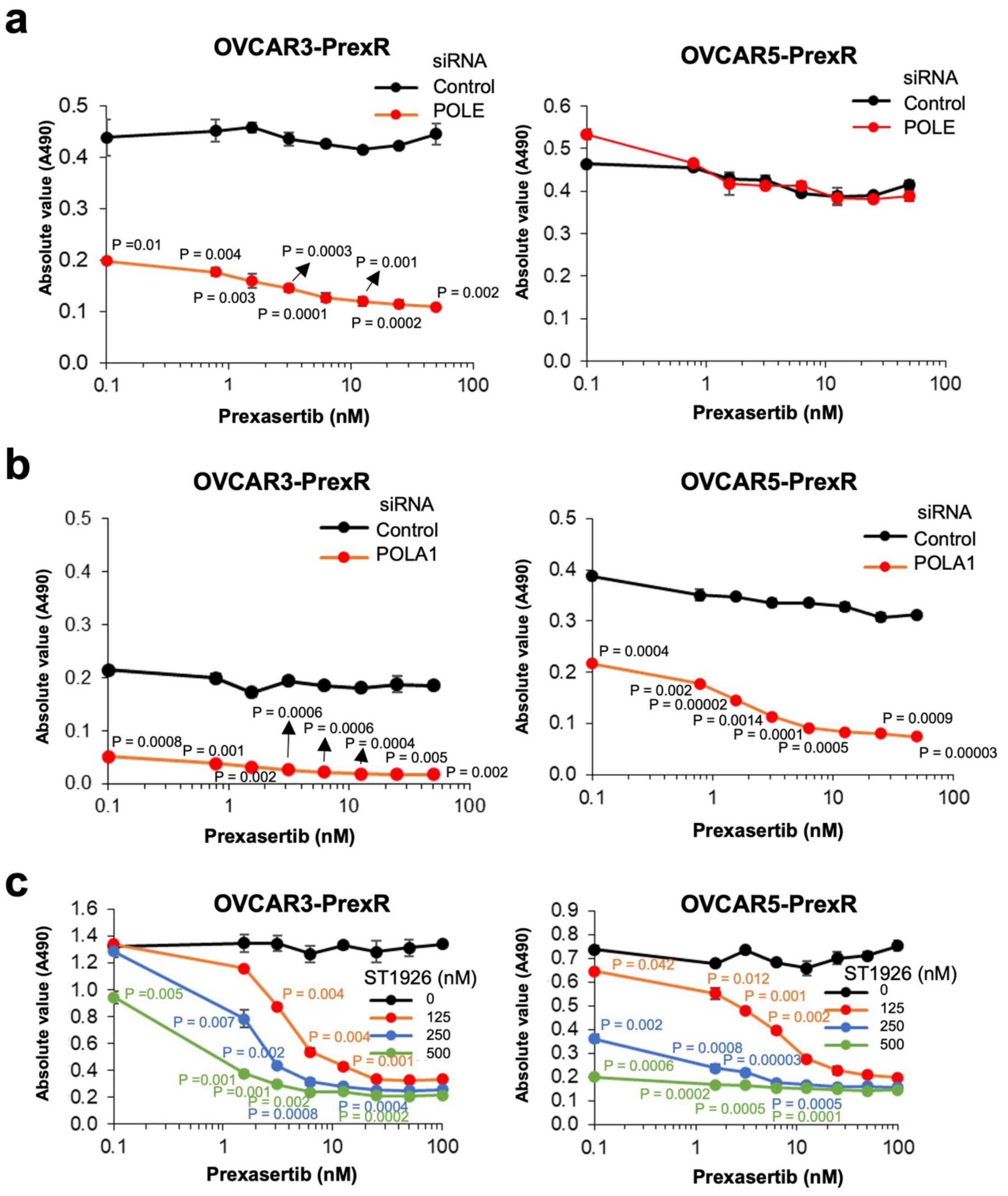

Supplementary Fig. 4: Targeting B-family polymerases to reverse CHK1i resistance in cisplatin-resistant HGSOC.

a-b XTT assays of PrexR variants of OVCAR3 and OVCAR5 that were either treated with control non-targeting siRNA or those that specifically target *POLE1* (**a**, n=3) or *POLA1* (**b**, n=3). The following day after transfection, cells were plated in 96-well plates and treated with increasing concentrations of CHK1i prexasertib (0-50 nM) for 48 hours. **c** XTT assays (n=3) with a POLA1 specific inhibitor ST1926 (0-500 nM) alone or in combination with prexasertib (0-100 nM). Absolute values (A490 nm) are plotted against CHK1i concentrations in PrexR variants of OVCAR3 and OVCAR5. Inhibition of cell growth was quantitated, and absolute absorption values are plotted

as line plots. Error bars indicate standard deviation values. Standard Student's *t*-test (two-sided) was used, and data are shown as mean ± SD. Source data are provided as a Source Data file.

Abbreviations: CHK1i, CHK1 inhibitor; HGSOC, high-grade serous ovarian cancer; PrexR, prexasertib-resistant; *POLA1*, DNA polymerase alpha 1; *POLE*, DNA polymerase epsilon.

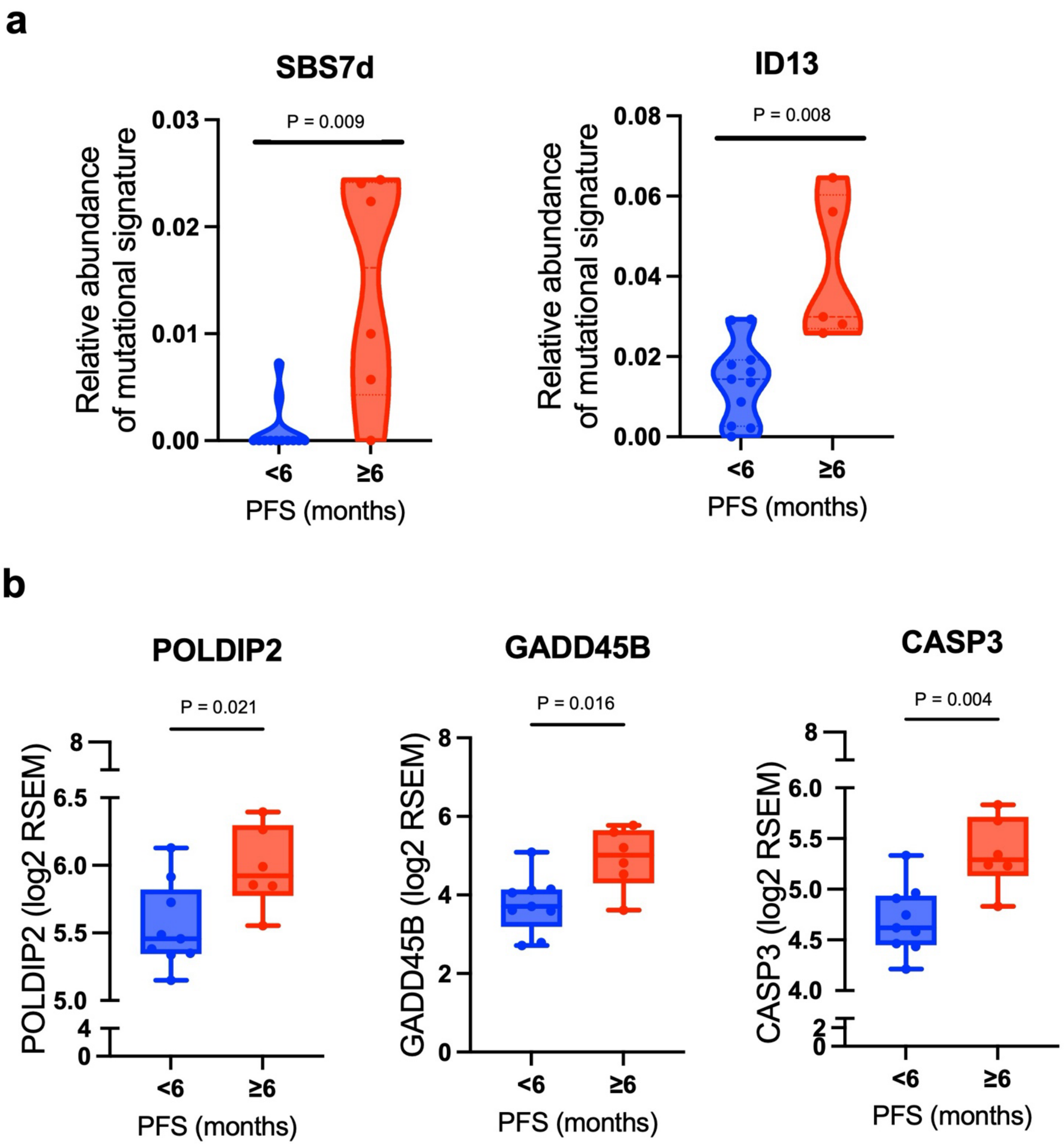

Supplementary Fig. 5: TLS-switching involvement in CHK1i response.

a Higher SBS7d and ID13 mutational signatures in patients with clinical benefit (n= 6, PFS ≥ 6 months) and no clinical benefit (n= 12, PFS < 6 months). Non-parametric Kruskal-Wallis test was used (pKW < 0.05 indicates significance, Supplementary Table 5). **b** High mRNA expression of genes involved in TLS-switching, and stress-induced response, *POLDIP2*, *GADD45B*, and *CASP3* are significantly associated with no clinical benefit (n= 9, PFS < 6 months) versus clinical benefit (n= 6, PFS ≥ 6 months). Regular *t*-test (two-sided) was used for raw P-value (Supplementary Data 5). The boxes extend from min to max values, with the median depicted by a horizontal line. Source data are provided as a Source Data file.

Abbreviations: CHK1i, CHK1 inhibitor; *CASP3*, caspase-3; *GADD45B*, growth arrest and DNA damage inducible beta; PFS, progression-free survival; *POLDIP2*, DNA polymerase delta interacting protein 2; TLS, translesion synthesis.

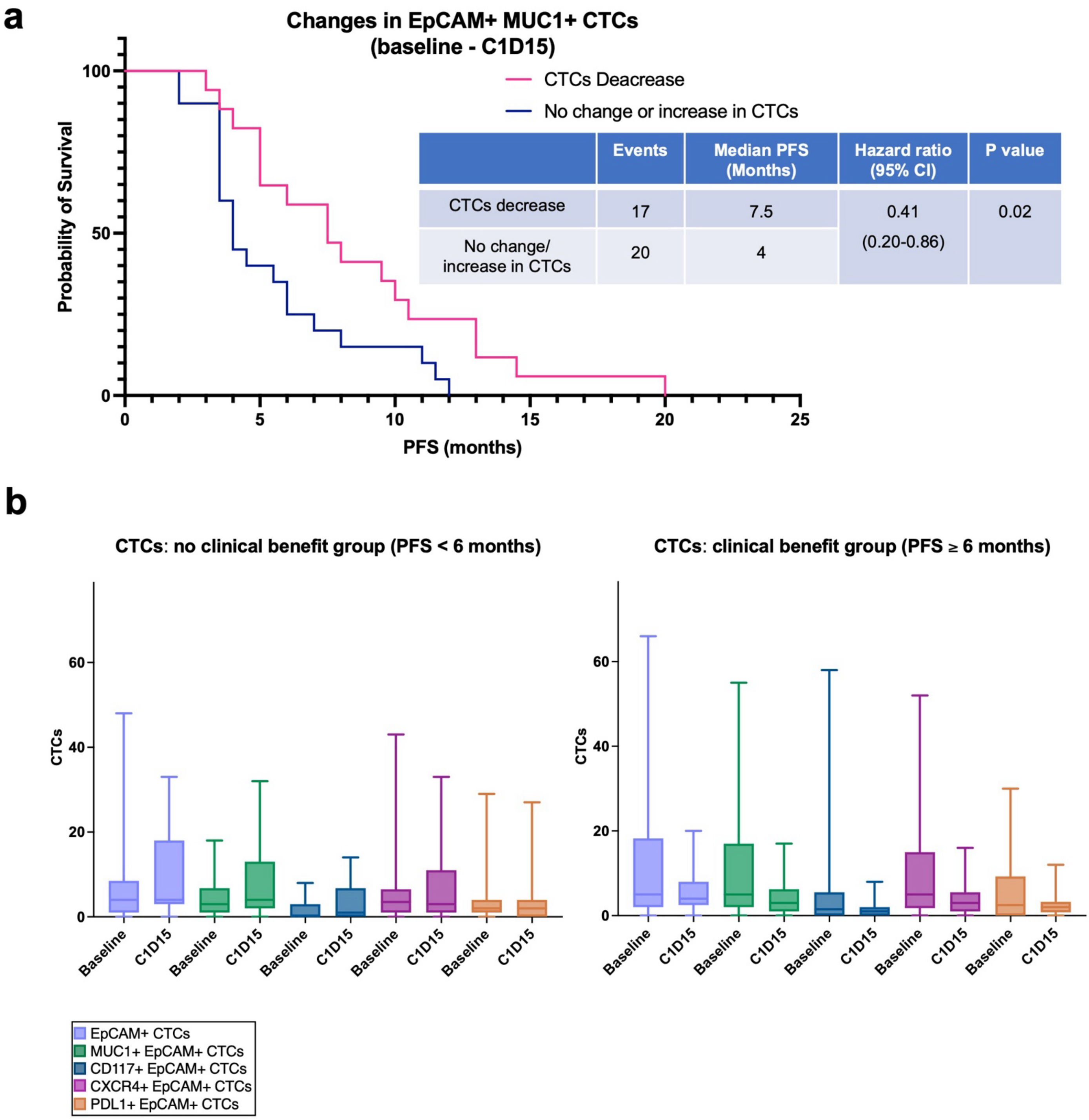

Supplementary Fig. 6: CTCs analyses.

a Kaplan–Meier estimates of PFS in patients with EpCAM+ MUC1+ CTCs versus no change or increase in EpCAM+ MUC1+ CTCs. Patients with decrease in EpCAM+ MUC1+ CTCs from baseline to C1D15 had a longer median PFS (7.5 months) compared to patients with no change or increase in EpCAM+ MUC1+ CTCs (4 months of median PFS). The PFS was assessed by a Kaplan-Meier survival plot, with the hazard ratio with 95% confidence intervals and logrank P value. **b** Dynamic changes of CTCs between baseline (no clinical benefit [n = 20] and clinical benefit [n = 18]) and C1D15 (no clinical benefit [n = 19] and clinical benefit [n = 18]). No changes

were observed in the count per tube of EpCAM+ CTCs, MUC1+ EpCAM+ CTCs, CD117+ EpCAM+ CTCs, CXCR4+ EpCAM+ CTCs, PDL1+ EpCAM+ CTCs in patients with and without clinical benefit. Non-parametric Wilcoxon rank sum test was used for unpaired samples, while Wilcoxon matched-pairs test was used to analyze paired samples. The boxes extend from min to max values, with the median depicted by a horizontal line. Source data are provided as a Source Data file.

Abbreviations: CTCs, circulating tumor cells; C1D15, cycle 1 day 15; CI, confidence interval; EpCAM, epithelial cell adhesion molecules; PFS, progression-free survival.

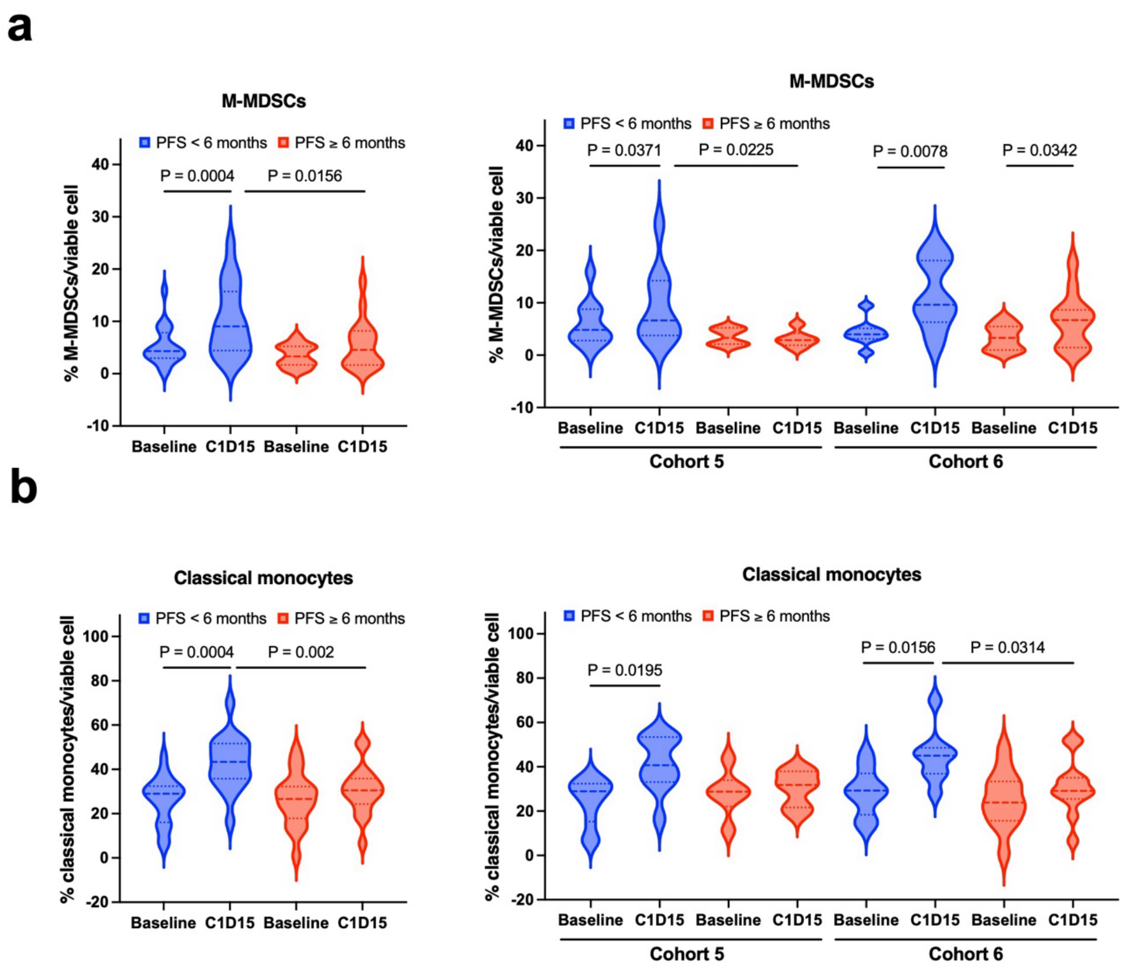

Supplementary Fig. 7: Changes in the systemic immune cell composition and functional changes upon CHK1i treatment.

a-b Dynamic changes of M-MDSCs (**a**) and classical monocytes (**b**) between baseline (PFS < 6 months [n = 19] and PFS ≥ 6 months [n=18]) and C1D15 (PFS < 6 months [n = 18] and PFS ≥ 6 months [n = 18]). Changes of M-MDSCs and classical monocytes in cohort 5 between baseline (PFS < 6 months [n = 11] and PFS ≥ 6 months [n=6]) and C1D15 (PFS < 6 months [n = 10] and PFS ≥ 6 months [n = 6]) and cohort 6 between baseline (PFS < 6 months [n = 8] and PFS ≥ 6 months [n=12]) and C1D15 (PFS < 6 months [n = 8] and PFS ≥ 6 months [n = 12]) were shown on the right. Non-parametric Wilcoxon rank sum test was used for unpaired samples, while Wilcoxon matched-pairs test was used to analyze paired samples. Source data are provided as a Source Data file.

Abbreviations: C1D15, cycle 1 day 15; M-MDSCs, monocytic myeloid-derived suppressor cells; PFS, progression-free survival.

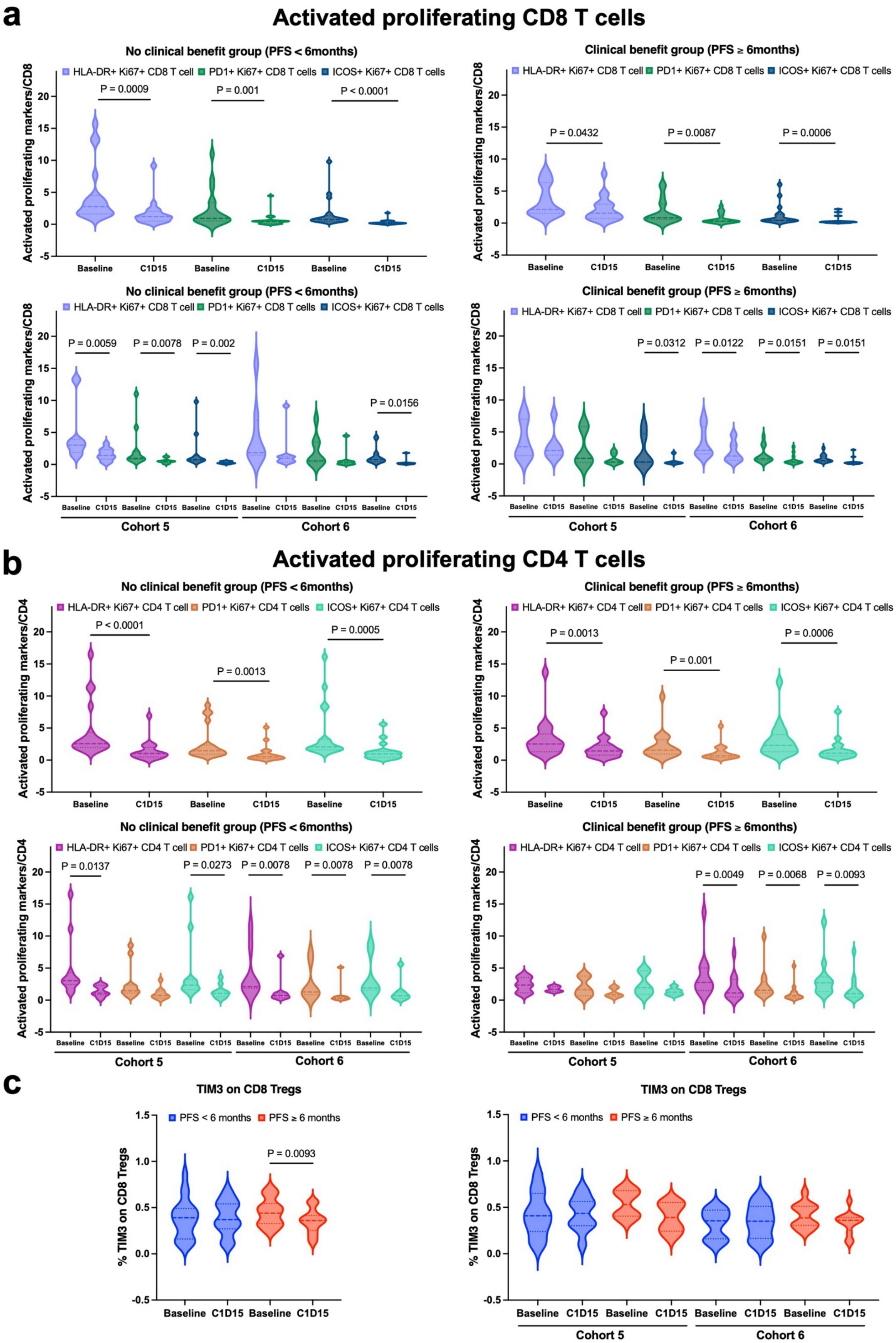
a
Activated proliferating CD8 T cells
No clinical benefit group (PFS < 6months)
Clinical benefit group (PFS ≥ 6months)
HLA-DR+ Ki67+ CD8 T cell
PD1+ Ki67+ CD8 T cells
ICOS+ Ki67+ CD8 T cells
Activated proliferating markers/CD8
P = 0.0009
P = 0.001
P < 0.0001
P = 0.0432
P = 0.0087
P = 0.0006
P = 0.0059
P = 0.0078
P = 0.002
P = 0.0156
P = 0.0312
P = 0.0122
P = 0.0151
P = 0.0151
Baseline
C1D15
Cohort 5
Cohort 6
b
Activated proliferating CD4 T cells
No clinical benefit group (PFS < 6months)
Clinical benefit group (PFS ≥ 6months)
HLA-DR+ Ki67+ CD4 T cell
PD1+ Ki67+ CD4 T cells
ICOS+ Ki67+ CD4 T cells
Activated proliferating markers/CD4
P < 0.0001
P = 0.0013
P = 0.0005
P = 0.0013
P = 0.001
P = 0.0006
P = 0.0137
P = 0.0273
P = 0.0078
P = 0.0078
P = 0.0078
P = 0.0049
P = 0.0068
P = 0.0093
Baseline
C1D15
Cohort 5
Cohort 6
c
TIM3 on CD8 Tregs
PFS < 6 months
PFS ≥ 6 months
% TIM3 on CD8 Tregs
P = 0.0093
Baseline
C1D15
Cohort 5
Cohort 6

Supplementary Fig. 8: Changes of activated proliferating CD4+ or CD8+ T-cells.

a-b Dynamic changes of T-cell activation markers, HLA-DR, or PD-1 or ICOS positive and proliferation marker (Ki67) positive T-cells on CD8 (**a**) and CD4 (**b**) between baseline (PFS < 6 months [n = 19] and PFS ≥ 6 months [n=18]) and C1D15 (PFS < 6 months [n = 18] and PFS ≥ 6 months [n = 18]) are shown. Changes of activated proliferating CD4+ or CD8+ T-cells in cohort 5 between baseline (PFS < 6 months [n = 11] and PFS ≥ 6 months [n=6]) and C1D15 (PFS < 6 months [n = 10] and PFS ≥ 6 months [n = 6]) and cohort 6 between baseline (PFS < 6 months [n = 8] and PFS ≥ 6 months [n=12]) and C1D15 (PFS < 6 months [n = 8] and PFS ≥ 6 months [n = 12]) are shown on the bottom. **c** Changes of TIM3 expression on CD8 Tregs between baseline (PFS < 6 months [n = 19] and PFS ≥ 6 months [n=18]) and C1D15 (PFS < 6 months [n = 18] and PFS ≥ 6 months [n = 18]) are shown. Changes of TIM3 expression on CD8 Tregs in cohort 5 between baseline (PFS < 6 months [n = 11] and PFS ≥ 6 months [n=6]) and C1D15 (PFS < 6 months [n = 10] and PFS ≥ 6 months [n = 6]) and cohort 6 between baseline (PFS < 6 months [n = 8] and PFS ≥ 6 months [n=12]) and C1D15 (PFS < 6 months [n = 8] and PFS ≥ 6 months [n = 12]) are shown on the right. Non-parametric Wilcoxon rank sum test was used for unpaired samples, while Wilcoxon matched-pairs test was used to analyze paired samples. Source data are provided as a Source Data file.

Abbreviations: PFS, progression-free survival; Tregs, regulatory T cells; TIM-3, T cell immunoglobulin and mucin domain-containing protein 3; PD1, programmed death 1; HLA-DR, Human leukocyte antigen DR antigen; ICOS, Inducible T-cell costimulatory.

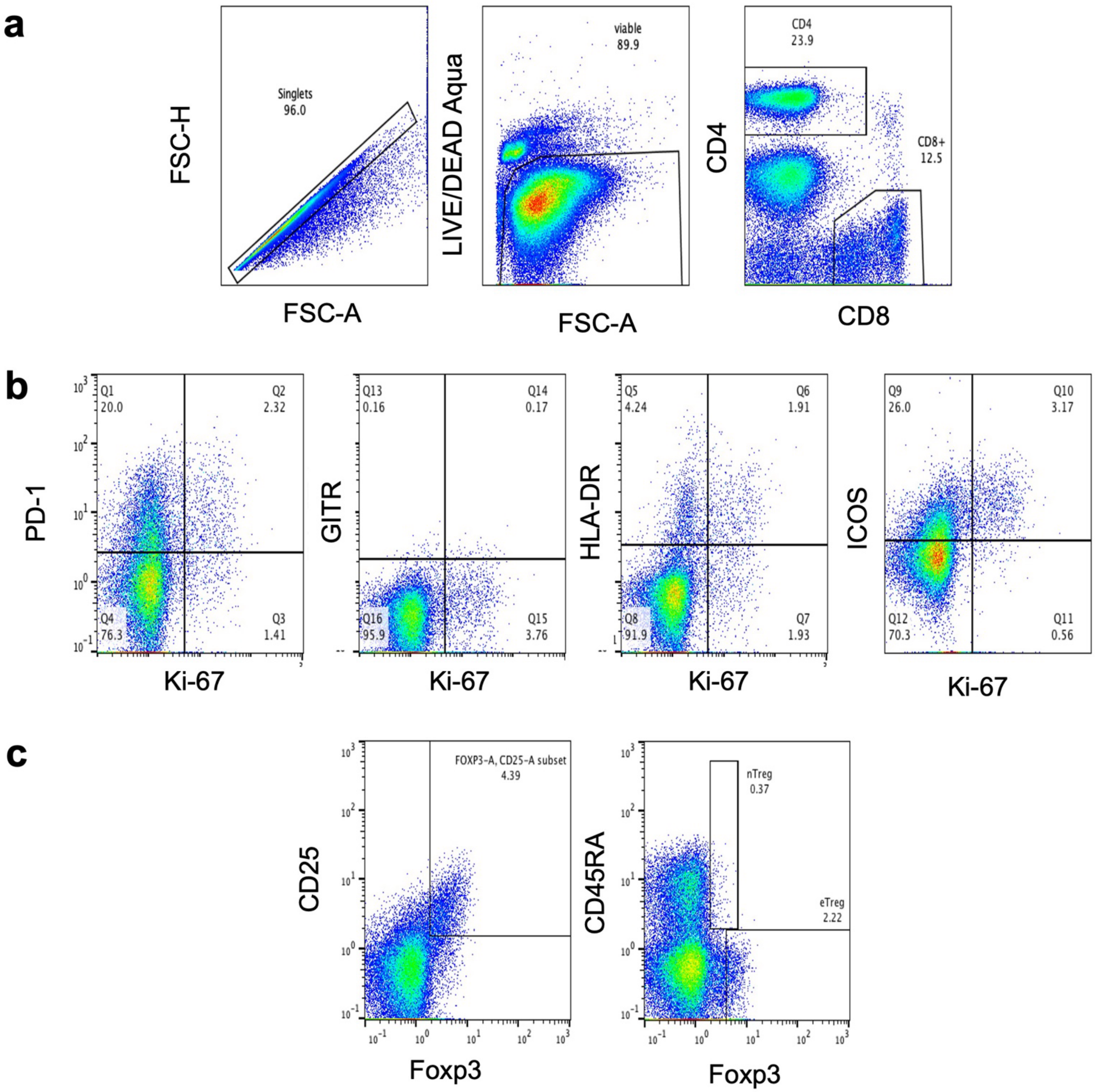

Supplementary Fig. 9: Representative raw data of flow gating strategy used to analyze CD4+ or CD8+ T-cells from patient PBMCs.

a Gating was done on PBMCs with doublet exclusion, then gating on viable cells, and then on subsets of CD4+ or CD8+ T-cell populations. **b** CD4+ or CD8+ T-cells were further gated for functional markers (PD-1, GITR, HLA-DR, ICOS or Ki-67); data shown are CD4+ T-cells. **c** CD4+ T- cells were gated for Treg (CD25+, Foxp3+), nTreg (CD45RA+, Foxp3 dim) or for eTreg (CD45RA-, Foxp3 high).

Abbreviations: PBMCs, peripheral blood mononuclear cells; Tregs, regulatory T-cells.

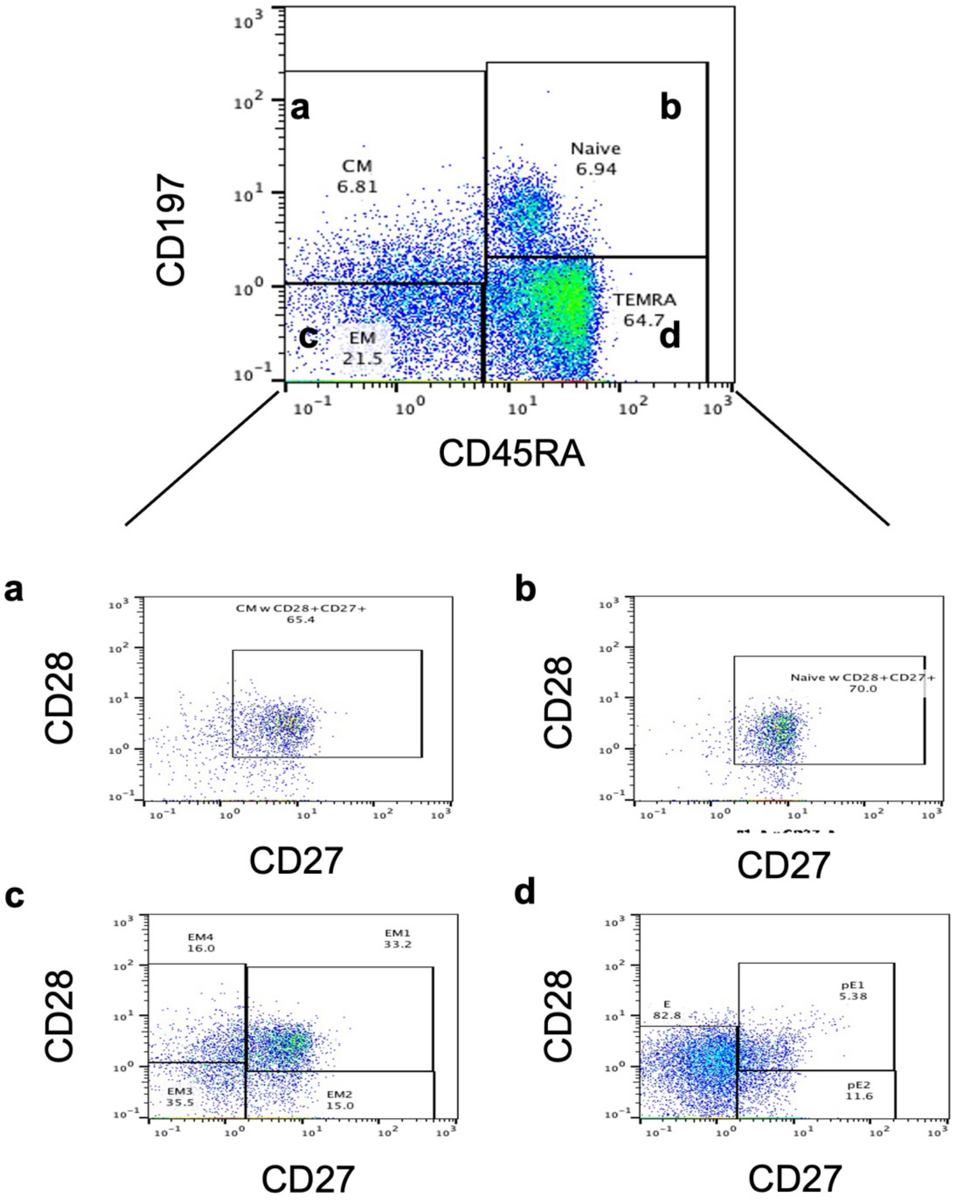

Supplementary Fig. 10: Representative raw data of flow gating strategy used to analyze Naïve, memory, effector CD4+ or CD8+ T-cells from patient PBMCs.

a-d Singlet, viable PBMCs were gated (not shown), then gating on viable cells, and then on subsets of CD4+ or CD8+ T-cell populations. **a** CM T-cells were defined as CD197+CD45RA-CD28+CD27+. **b** Naïve T-cells were defined as CD197+CD45RA+CD28+CD27+. **c** EM T-cells were subdivided by EM1 (CD197-CD45RA-CD28+CD27+), EM2 (CD197-CD45RA-CD28-CD27+), EM3 (CD197-CD45RA-CD28-CD27-) and EM4 (CD197-CD45RA-CD28+CD27-). **d** TEMRA T-cells were subdivided by effector (CD197-CD45RA+CD28+dimCD27+), pE1 (CD197-CD45RA+CD28+CD27+), pE2 (CD197-CD45RA+CD28-CD27+).

Abbreviations: PBMCs, peripheral blood mononuclear cells; CM, central memory; EM, effector memory; TEMRA, terminally differentiated effector memory.

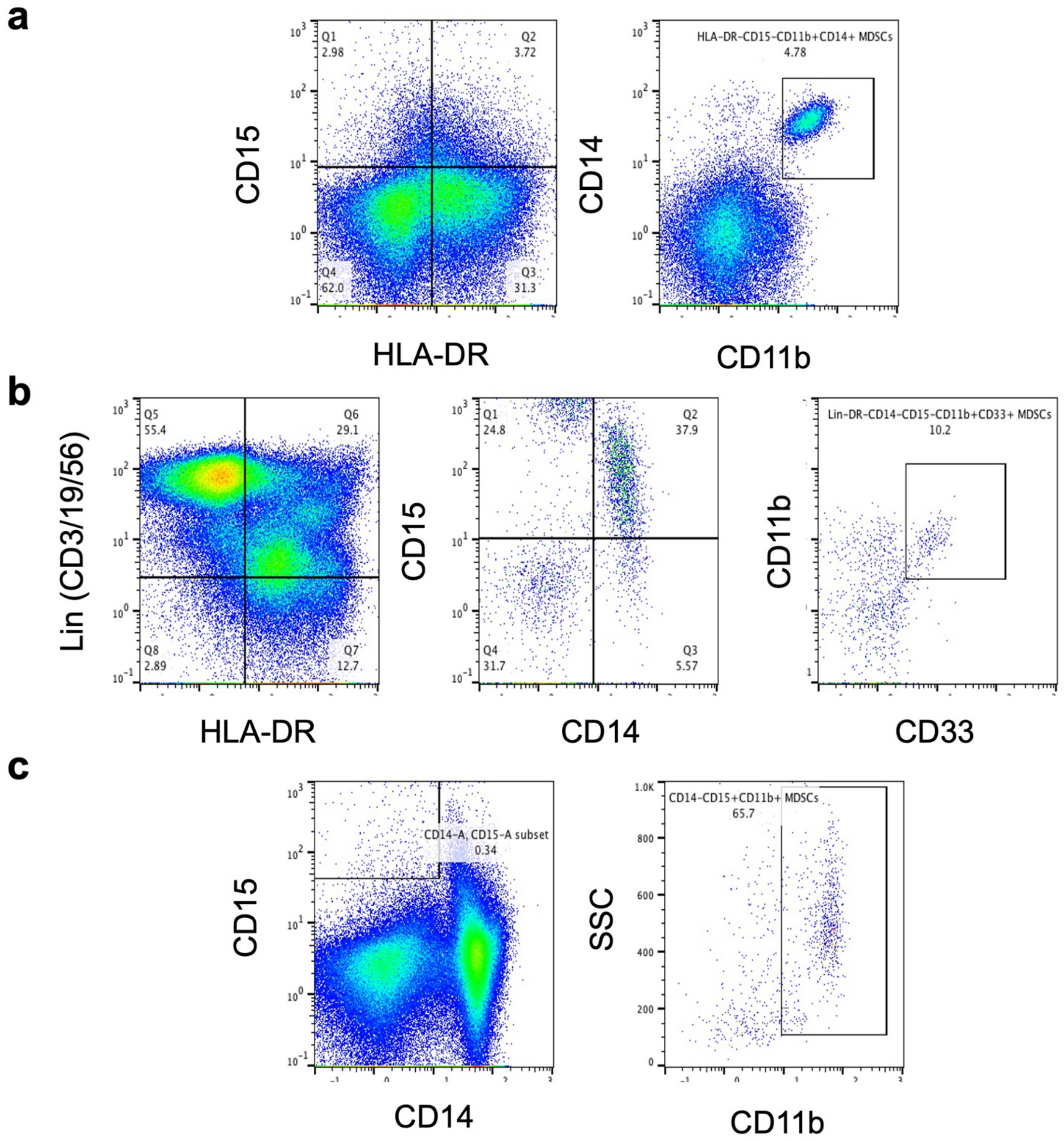

Supplementary Fig. 11: Representative raw data of flow gating strategy used for identification of MDSC subsets from patient PBMC.

Singlet, viable PBMCs were gated (not shown), and then (**a**) M-MDSCs were gated were defined as HLA-DR-CD15-CD11b+CD14+ (**b**), eMDSCs were defined as Lineage-(CD3-CD19-CD56-) CD14-CD15-HLA-DR-CD11b+CD33+ (**c**), and PMN-MDSCs were defined as CD11b+CD14-CD15+.

Abbreviations: MDSC, myeloid-derived suppressor cell; PBMCs, peripheral blood mononuclear cells; M-MDSCs, monocytic MDSCs; eMDSCs, early-stage MDSCs; PMN-MDSCs, polymorphonuclear MDSCs.

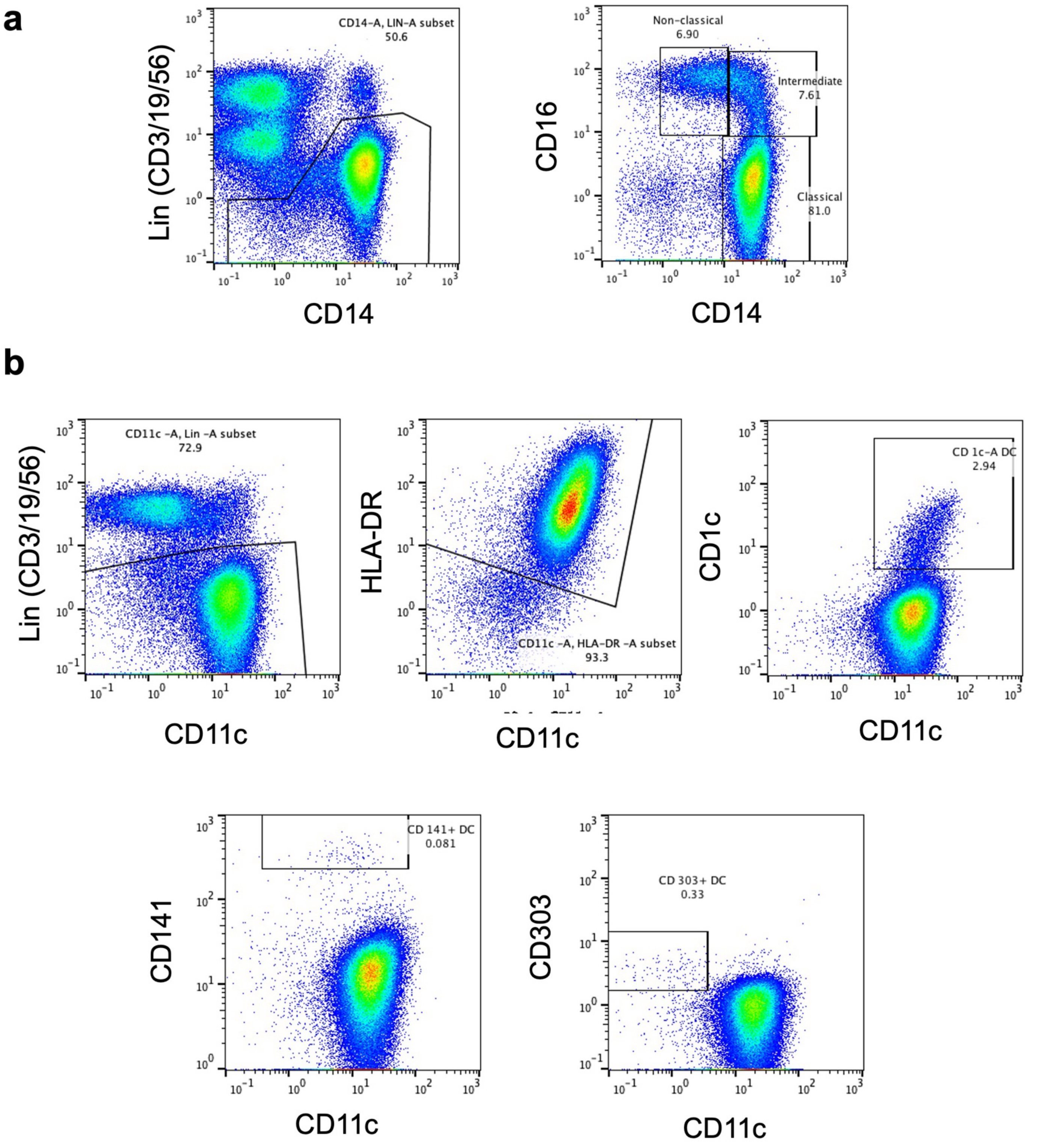

Supplementary Fig. 12: Monocytes and DCs gating strategies.

a Representative raw data of flow gating strategy used for identification of monocyte subsets from patient PBMC. Singlet, viable PBMCs were gated (not shown), and then total monocytes (CD14+CD3-CD19-CD56-) were subdivided by CD14 and CD16 expression: classical monocytes (CD14++CD16-); non-classical monocytes (CD14+CD16++); and intermediate monocytes (CD14++CD16+). **b** Representative raw data of flow gating

strategy used for identification of DC subsets from patient PBMC. Singlet, viable PBMCs were gated (not shown), and then lineage - (CD3-CD19-CD56-), HLA-DR+ cells were gated then subdivided into three different DC subsets CD1c+ mDCs, CD141+ mDCs and CD303+ pDCs.

Abbreviations: DCs, dendritic cells; PBMCs, peripheral blood mononuclear cells; mDC, myeloid DCs; pDCs, plasmacytoid DC.

Supplementary Table 1. Patient and disease characteristics at baseline.

Characteristic/demographic	All (n=49)	Cohort 5 (n=24)	Cohort 6 (n=25)
Age – years			
Median (IQR)	64.1 (56.8-69.9)	63 (56-67)	68.4 (57.9-72.9)
ECOG – n (%)			
0	7 (14.3%)	2 (8.3%)	5 (20%)
1	42 (85.7%)	22 (91.7%)	20 (80%)
2	0 (0%)	0 (0%)	0 (0%)
Platinum resistance status* – n (%)			
Primary	10 (20.4%)	4 (16.7%)	6 (24%)
Secondary	39 (79.6%)	20 (83.3%)	19 (76%)
Number of previous treatments – n (%)			
1	0 (0%)	0 (0%)	0 (0%)
2	8 (16.3%)	5 (20.8%)	3 (12%)
≥ 3	39 (79.6%)	17 (70.8%)	22 (88%)
Previous treatments – n (range, %)			
Number of systemic therapy lines	4 (3-7)	5 (3-6)	4 (3-6)
Number of cytotoxic chemotherapeutic agents	4 (3-6)	4 (2-9)	4 (3-6)
Radiotherapy	5 (10.2%)	2 (8.3%)	3 (12%)
Vaccines	3 (6.1%)	3 (12.5%)	0 (0%)
PARP inhibitors	22 (44.9%)	11 (45.8%)	11 (44%)
Bevacizumab	39 (79.6%)	15 (62.5%)	24 (96%)
VEGFR TKI inhibitor (cediranib)	2 (4.1%)	1 (4.2%)	1 (4%)
Immunotherapy (Immune checkpoint inhibitors and autologous transgenic T-cell infusion)	10 (20.4%)	7 (29.2%)	3 (12%)
Baseline CA-125 concentration - n (%)			
Normal (1.9-16.3 U/mL)	1 (2.0%)	1 (4.2%)	0 (0%)
Elevated (≥16.3 U/mL)	48 (98.0%)	23 (95.8%)	25 (100%)

* Patients were defined as having primary platinum-resistant disease if progression of disease occurred < 6 months after completing first-line platinum-based therapy or having secondary platinum-resistant disease if progression of disease occurred ≥ 6 months after first-line platinum-based therapy but < 6 months after second-line or last-line platinum-based therapy.

Supplementary Table 2. DNA-damage response and replication stress-associated genes detected with WES: gene list

Genes			
53BP1	ERBB3	NRAS	SHLD3
ABCB1	ERBB4	ORC6	SLFN11
ABCG2	EXO1	PALB2	SMARCAD1
AKT	EZH2	PARG	SMARCAL1
AKT1	FAM122A	PARP1	SRSF1
AKT2	FANCA	PI3K	STN1
AKT3	FANCC	PIK3CA	SUV39H1
APE	FANCD2	PIK3R1	TEN1
ARID1A	FANCE	PMS2	TET2
ATM	FANCF	POLA1	TNPO2
ATR	FANCG	POLD4	TP53
ATRX	FANCI	POLE4	TRIP13
AURKB	FANCL	POLQ	WEE1
BARD1	FANCM	PRPS1	WRN
BLM	FAT3	PTEN	ZRANB3
BRAF	GADD45G	PTIP	
BRCA1	GINS1	RAD50	
BRCA2	HLTF	RAD51	
BRIP1	KPNA2	RAD51B	
CCNB1	KRAS	RAD51C	
CCND1	LIG3	RAD51D	
CCND2	MAD2L2	RAD52	
CCNE1	MAP2K4	RAD54L	
CDK1	MAP3K1	RADX	
CDK4	MLH1	RB1	
CDK6	MLL	RECQL1	
CDKN1B	MRE11	RECQL4	
CDKN2A	MRE11A	RECQL5	
CDKN2B	MSH2	REV7	
CHD4	MSH6	RFC5	
CHEK1	MTF2	RICTOR	
CHEK2	MUS81	RIF1	
CTC1	MUTYH	RMI1	
DYNLL1	MYC	RPA1	
EMI1	MYCL1	RRM1	
EMSY	NBN	SHLD1	
ERBB2	NF1	SHLD2	

Supplementary Table 3. Differential mutational signatures according to proposed etiology of clinical benefit vs. no clinical benefit (WES analysis).

Proposed etiologies	Associated mutational signatures	pKW**	P_sum***
UV (SBS7a, SBS7b, SBS7c, SBS7d, DBS1, ID13)	SBS7a	0.158	0.002
	SBS7b	0.297	
	SBS7c	0.114	
	SBS7d	0.009	
	DBS1	0.491	
	ID13	0.008	
SlippageDNAstrng (ID1, ID2)	ID1	0.491	0.099
	ID2	0.041	
POLD1 (SBS10c*, SBS10d)	SBS10d	0.325	0.325
CN: FocalLOH (CN9, CN10, CN11, CN12)	CN9	0.11	0.347
	CN10	0.325	
	CN11	0.859	
	CN12	0.371	
DNAMMR (SBS6, SBS15, SBS21, SBS26, SBS44, DBS7, DBS10, ID7)	SBS6	0.809	0.438
	SBS15	0.955	
	SBS21	0.139	
	SBS26	0.138	
	SBS44	0.5	
	DBS7	0.361	
	DBS10	0.289	
	ID7	0.388	
CN: Chromothripsis (CN4, CN5*, CN6, CN7, CN8)	CN4	0.325	0.497
	CN6	0.5	
	CN7	0.371	
	CN8	0.417	
CN:LOH (CN13, CN14, CN15, CN16)	CN13	0.325	0.524
	CN14	0.329	
	CN15	0.595	
	CN16	0.449	
APOBEC (SBS2, SBS13)	SBS2	0.28	0.637
	SBS13	1	
HR (SBS3*, ID6)	ID6	0.65	0.65
Tobacco (SBS4, SBS92*, ID3)	SBS4	0.622	0.811
	ID3	0.727	
Polymerase epsilon exonuclease (SBS10a, SBS10b, DBS3)	SBS10a	0.535	0.828
	SBS10b	0.856	
	DBS3	0.527	
E. coli (SBS88, ID18*)	SBS88	0.95	0.95
Platinum (SBS31, SBS35, DBS5)	SBS31	1	0.938
	SBS35	0.767	
	DBS5	0.532	

*Missing signatures in patients' samples.
**non-parametric Kruskal-Wallis test (pKW < 0.05 indicates significance)
***Grouped p-values according to etiology were obtained using a Fisher exact method.

Supplementary Table 4. Antibodies used in this study.

Target	Clone	Company	Dilutions	Catalog #
CD11b	ICRF44	BioLegend	1:50	301318
CD11c	Bu15	BioLegend	1:50	337218
CD14	HCD14	BioLegend	1:50	325622
CD141	M80	BioLegend	1:50	344114
CD15	W6D3	BioLegend	1:100	323040
CD16	3G8	BioLegend	1:50	302018
CD19	H1B19	BioLegend	1:50	302220
CD197	G043H7	BioLegend	1:50	353204
CD1c	L161	BioLegend	1:50	331522
CD25	BC96	BioLegend	1:50	302612
CD27	LG.3A10	BioLegend	1:100	124222
CD279 (PD1)	EH12.2H7	BioLegend	1:50	329920
CD28	CD28.2	BioLegend	1:50	302966
CD3	OKT3	BioLegend	1:50	317312
CD303	201A	BioLegend	1:50	354210
CD33	WM53	BioLegend	1:50	303404
CD366 (TIM-3)	F38-2E2	BioLegend	1:50	345008
CD4	RPA-T4	BioLegend	1:50	357408, 357410
CD45RA	HI100	BioLegend	1:50	304122
CD56	5.1H11	BioLegend	1:50	304612
CD8	SK1	BioLegend	1:50	344714, 344748
CD83	HB15e	BioLegend	1:50	305308
CTLA-4	L3D10	BioLegend	1:50	349906
Foxp3	206D	BioLegend	1:50	320112
GITR	108-17	BioLegend	1:50	371204
HLA-DR	L243	BioLegend	1:50	307620, 307616
ICOS	C398.4A	BioLegend	1:50	313518
Ki-67	ki-67	BioLegend	1:50	350510
PE-conjugated anti-EpCAM	HEA-125	Miltenyi Biotec	1:200	130-091-253
CD45	clone HI30	BioLegend	1:50	304014
CD117	104D2	BioLegend	1:100	313212
CXCR4	12G5	BioLegend	1:100	306516
PDL1	29E.2A3	BioLegend	1:100	329708
MUC-1	HMPV	BD Biosciences	1:50	559774
CHK1	2G1D5	Cell signaling technology	1:2000	2360
CHK1-S345	133D3	Cell signaling technology	1:2000	2348
CHK1-S296	polyclonal	Cell signaling technology	1:2000	2349
KAP1-S824	polyclonal	Cell signaling technology	1:2000	4127
KAP1	4E1	Cell signaling technology	1:2000	5868
b-ACTIN	8H10D10	Cell signaling technology	1:2000	3700
POLE	polyclonal	ThermoFisher Scientific	1:2000	PA5-78113
POLA1	polyclonal	Abcam	1:2000	Ab31777
Anti-mouse IgG HRP	--	Cell signaling technology	1:3000	7076
Anti-rabbit IgG HRP	--	Cell signaling technology	1:3000	7074

Supplementary Table 5. Immune phenotypes and functional markers used for flow cytometric analysis

Immune subset	Phenotype	Functional Marker
Myeloid-derived suppressor cells (MDSCs)		
Monocytic MDSC (mMDSC)	CD11b+ CD14+ HLA-DR-/low CD15-	
Early MDSC (eMDSC)	Lin- (CD3, CD19, CD56)- CD14- HLA-DR- CD11b+CD33+	
Polymorphonuclear MDSC (PMN-MDSC)	CD14- CD11b+ CD15+	
Monocytes		
Classical Monocytes	CD14++ CD16-	HLADR
Intermediate Monocytes	CD14++ CD16+	
Non-classical Monocytes	CD14 dim CD16++	
Dendritic cells (DCs)		
CD1c+mDC	Lin- HLA-DR+ CD11c+ CD1c+	CD83
CD141+mDC	Lin- HLA-DR+ CD11c+ CD141+	
CD303+pDC	Lin- HLA-DR+ CD11c-/dim CD303+	
Regulatory T Cells (Tregs)		
Effector Treg (eTreg)	CD4+ CD45RA- Foxp3high	CTLA4 and TIM-3
naïve Treg (nTreg)	CD4+ CD45RA+ Foxp3low	
Treg	CD25+ Foxp3+ (CD4+ or CD8+)	
T cell activation, proliferation		
CD4+ T cells / CD8+ T cells	CD4+CD8- / CD8+ CD4-	HLA-DR, GITR, ICOS, PD-1, Ki67
Memory/naïve T-cells		
Central memory (CM) T cells	CD45RA- CD197+ CD28+ CD27+	
	(CD4+ or CD8+)	
naïve T cells	CD45RA+ CD197+ CD28+ CD27+	
	(CD4+ or CD8+)	
effector (E) T cells	CD45RA+ CD197- CD28- CD27-	
	(CD4+ or CD8+)	
effector memory (EM)1 T cells	CD45RA- CD197- CD28+ CD27+	
	(CD4+ or CD8+)	
EM2 T cells	CD45RA- CD197- CD28- CD27+	
	(CD4+ or CD8+)	
EM3 T cells	CD45RA- CD197- CD28- CD27-	
	(CD4+ or CD8+)	
EM4 T cells	CD45RA- CD197- CD28+ CD27-	
	(CD4+ or CD8+)	

Abbreviations: Lin-, Lineage negative; Foxp3, Forkhead box P3; CTLA-4, Cytotoxic T-lymphocyte antigen 4; TIM-3, T cell immunoglobulin and mucin domain-containing protein 3; PD1, programmed death 1; HLA-DR, Human leukocyte antigen DR antigen; ICOS, Inducible T-cell costimulatory; GITR, Glucocorticoid-induced TNFR-related protein

Supplementary Note: Study Protocol

Abbreviated Title: LY Chk1/2 Inhibitor

NIH Protocol #: 14-C-0156
Version Date: 11/13/2023
NCT Number: NCT02203513

Title: A phase II single arm pilot study of the Chk1/2 inhibitor (LY2606368) in BRCA1/2 mutation associated breast or ovarian cancer, triple negative breast cancer, and high grade serous ovarian cancer

NCI Principal Investigator: Jung-Min Lee, MD
Women's Malignancies Branch, CCR, NCI
Building 10, Room 4B54
National Institutes of Health
9000 Rockville Pike
Bethesda, MD 20892
Phone: (240) 760-6128
Email: leej6@mail.nih.gov

Investigational Agents:

Drug Name:	LY2606368 (Prexasertib)
IND Number:	123172
Sponsor:	NCI, Center for Cancer Research
Manufacturer:	Eli Lilly
Supplier:	Eli Lilly

STUDY SCHEMA

Dose Level (DL)	LY2606368 IV q2w (mg/m^2)
Starting dose, DL 1	105
DL-1	80
DL -2	60

Effective with amendment I (version date 04/24/2017), the prostate cancer cohort 4 was closed. Effective with 4/17/2020, the study was closed for new enrollment because Eli Lilly withdrew research sponsorship for drug supplies on 2/19/2020.

Study schema

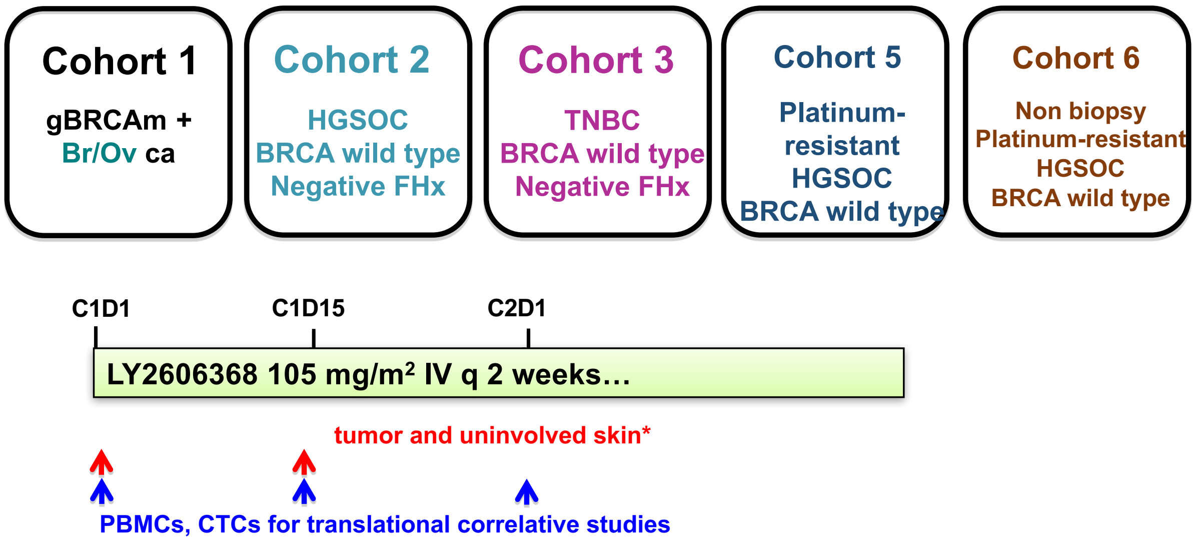

PRÉCIS

Background:

- Checkpoint kinases 1 and 2 (Chk1/2) are major regulators of the cell cycle and are intimately associated with the cellular response to DNA damage and repair. Chk1/2 also function as the primary mediators of cell cycle arrest in tumors with p53 dysfunction, such as high-grade serous ovarian cancer (HGSOC), and triple negative breast cancer (TNBC).
- Participants with germline BRCA1 or BRCA2 mutation have inherent defects in DNA damage repair pathways.
- Chk1/2 inhibition alone yielded DNA damage and mitotic catastrophe preclinically, even in the absence of DNA damage by external agents in tumors with underlying DNA repair dysfunction.
- The second-generation Chk1/2 inhibitor, LY2606368, yielded safety and preliminary single agent activity in advanced cancer participants.
- *We hypothesize that LY2606368 will result in clinical benefit in participants with gBRCAm-associated breast or ovarian cancers, and HGSOC and TNBC with low genetic risk.*

Objectives:

- To determine the objective response rate (CR+PR) of single agent LY2606368 in patients with gBRCAm-associated breast or ovarian cancer, HGSOC and TNBC with low genetic risk.
- To determine the safety and toxicity, and progression-free interval (PFI) of LY2606368 in pretreated participants.
- To determine biochemical changes in the DNA damage repair and cell cycle check point pathways in tumor and blood samples in response to treatment.
- To determine potential resistance mechanisms to LY2606368 treatment in HGSOC.

Eligibility:

- Participants with recurrent/refractory BRCA mutant breast or ovarian cancer, HGSOC, and TNBC, for whom there remains no standard curative measures.
- A documented deleterious germline or somatic BRCA mutation for breast or ovarian cancer participants enrolling in Cohort 1.
- Negative BRCA mutation testing or negative family history of hereditary breast and ovarian cancer syndrome for HGSOC (Cohort 2).
- Negative BRCA mutation testing or negative family history of hereditary breast and ovarian cancer syndrome for TNBC (Cohort 3).
- Effective with amendment I (version date 4/24/2017), mCRPC, Cohort 4 was closed.
- Negative BRCA mutation testing or negative family history of hereditary breast and ovarian cancer syndrome for recurrent platinum-resistant HGSOC with measurable and biopsiable disease (Cohort 5).

- Negative BRCA mutation testing or negative family history of hereditary breast and ovarian cancer syndrome for recurrent platinum-resistant HGSOC with measurable but *without* biopsiable disease (Cohort 6).
- Participants must be off prior chemotherapy, radiation therapy, hormonal therapy, or biological therapy for at least 4 weeks.
- ECOG performance status 0-2 and adequate organ and marrow function.

Design:

- This is an open label, single arm phase II trial to examine activity of LY2606368 in participants in the 6 independent cohorts (Cohorts 1-6).
- LY2606368 will be dosed at the RP2D of 105 mg/m^2 IV once every 14 days of a 28day-cycle.
- Research samples including whole blood, CTCs, and tumor biopsies will be obtained for PD endpoints at baseline, Cycle 1 Day 15 (6-24hr post-2nd dose), and/or at progression in all participants. Tumor biopsies will not be performed in Cohort 6.
- Participants (Cohorts 1-3, 5 and 6) will be evaluated every two cycles for response using RECIST v1.1 and every cycle for safety using CTCAE v4.0.

TABLE OF CONTENTS

STUDY SCHEMA 2
PRÉCIS 3
TABLE OF CONTENTS 5
STATEMENT OF COMPLIANCE 9
1. INTRODUCTION 9
1.1 Study Objectives 9
1.1.1 Primary Objective: 9
1.1.2 Secondary Objectives: 9
1.1.3 Exploratory Objectives: 9
1.2 Background and Rationale 10
2 ELIGIBILITY ASSESSMENT AND ENROLLMENT 22
2.1 Eligibility Criteria 22
2.1.1 Inclusion Criteria 22
2.1.2 Exclusion Criteria 26
2.2 Participant Registration and Status Update Procedures 27
2.3 Treatment Assignment and Randomization/Stratification Procedures 27
2.4 Screening and Baseline Evaluations 28
2.4.1 Imaging Studies 28
2.4.2 An EKG should be obtained within 1 week prior to enrollment. 28
2.4.3 Laboratory Evaluations [baseline is to be obtained within 1 week prior to enrollment]. 28
3 STUDY IMPLEMENTATION 29
3.1 Study Design 29
3.2 Drug Administration 30
3.3 Dose Modifications 31
3.3.1 Hematologic Toxicity 32
3.3.2 QTc Prolongation 33
3.3.3 Non-hematologic Toxicity 34
3.4 Study Calendar 35
3.5 Cost and Compensation 38
3.5.1 Cost 38
3.5.2 Compensation 38
3.5.3 Reimbursement 38
3.5.4 Criteria for removal from protocol therapy 38
3.5.5 Off-Study Criteria 38
3.5.6 Lost to follow up 39
4 CONCOMITANT MEDICATIONS/MEASURES 39
5 CORRELATIVE STUDIES AND BIOSPECIMEN COLLECTION 40
5.1 Correlative Studies for Research/Pharmacodynamic Studies 40
5.1.1 Rationale for Correlative Studies 40

5.1.2 Tumor biopsies ... 41
5.1.3 Laboratory Correlative Studies ... 42
5.1.4 Blood Samples ... 45
5.1.5 Other body fluids samples ... 47
5.1.6 Radiomics ... 47
5.2 Sample Storage, Tracking and Disposition ... 49
5.2.1 Clinical Pharmacology Program ... 49
5.3 Samples for Correlative studies Analysis ... 50
5.3.1 Management of Correlative Studies Results ... 50
6 DATA COLLECTION AND EVALUATION ... 51
6.1 Data Collection ... 51
6.2 Data Sharing Plan ... 52
6.2.1 Human Data Sharing Plan ... 52
6.2.2 Genomic Data Sharing Plan ... 53
6.3 Response Criteria ... 53
6.3.1 Definitions ... 53
6.3.2 Disease Parameters ... 54
6.3.3 Methods for Evaluation of Measurable Disease ... 55
6.3.4 Response Criteria ... 56
6.3.5 Duration of Response ... 57
6.4 Toxicity Criteria ... 58
7 NIH REPORTING REQUIREMENTS/DATA AND SAFETY MONITORING PLAN ... 58
7.1 Definitions ... 58
7.2 OHSRP Office of Compliance and Training / IRB Reporting ... 58
7.2.1 Expedited Reporting ... 58
7.2.2 IRB Requirements for PI Reporting at Continuing Review ... 58
7.3 NCI Clinical Director Reporting ... 59
7.4 NIH Required Data and Safety Monitoring Plan ... 59
7.4.1 Principal Investigator/Research Team ... 59
7.4.2 Central Reading of Scans ... 59
8 SPONSOR SAFETY REPORTING ... 60
8.1 Definitions ... 60
8.1.1 Adverse Event ... 60
8.1.2 Serious Adverse Event (SAE) ... 60
8.1.3 Life-threatening ... 61
8.1.4 Severity ... 61
8.1.5 Relationship to Study Product ... 61
8.2 Assessment of Safety Events ... 61
8.3 Reporting of Serious Adverse Events ... 62
8.4 Safety Reporting Criteria to the Pharmaceutical Collaborators ... 62
8.5 Reporting Pregnancy ... 63
8.5.1 Maternal exposure ... 63
8.5.2 Paternal exposure ... 63

8.6 Regulatory Reporting for Studies Conducted Under CCR-Sponsored IND............... 64
9 CLINICAL Monitoring 64
10 STATISTICAL CONSIDERATIONS 64
11 COLLABORATIVE AGREEMENTS 67
11.1 Cooperative Research and Development Agreement (CRADA)............................... 67
11.1.1 Eli Lilly and Company 67
12 HUMAN SUBJECTS PROTECTIONS 68
12.1 Rationale For Subject Selection 68
12.2 Participation of Children 68
12.3 Participation of Subjects Unable to Give Consent 68
12.4 Evaluation of Benefits and Risks/Discomforts 68
12.4.1 Risks of Radiation 69
12.4.2 Risk of Treatment: Details of the risk of drug therapy are detailed in section 13. 69
12.4.3 Blood draws: Side effects of blood draws include pain and bruising, lightheadedness, and rarely, fainting. 69
12.4.4 CT scans: In addition to the radiation risks discussed above, risks associated with CT scans are allergic reaction to and kidney damage from the contrast dye, nausea, vomiting, and anxiety. 69
12.4.5 Biopsies: The non-radiation risks associated with the biopsy procedure include pain, swelling and/or bleeding at the biopsy site; infection; and allergic reaction to the local anesthetic used. 69
12.5 Risks/Benefits Analysis 69
12.6 Consent Process and Documentation 70
12.6.1 Consent Process for Adults Who Lack Capacity to Consent to Research Participation.. 70
13 REGULATORY AND OPERATIONAL CONSIDERATIONS 70
13.1 Study Discontinuation and Closure 70
13.2 QUALITY ASSURANCE AND QUALITY CONTROL 71
13.3 CONFLICT OF INTEREST POLICY 71
13.4 Confidentiality and Privacy 71
14 PHARMACEUTICAL INFORMATION 72
14.1 LY2606368 72
14.1.1 Source 72
14.1.2 Toxicity 73
14.1.3 Formulation and preparation 76
14.1.4 Stability and Storage 76
14.1.5 Administration procedures 76
14.1.6 Incompatibilities 76
15 REFERENCES 77
16 APPENDICES 80
16.1 Appendix A – Performance Status Criteria 80

16.2 Appendix B – Drugs known to cause QT prolongation and/or induce torsades de pointes 81
16.3 Appendix C – Laboratory Standard Operating Procedure- Tissue Core Collection... 83
16.4 Appendix D – Hereditary Breast/Ovarian Cancer Syndrome Family History Criteria (modified from NCCN, 2013)........ 85

STATEMENT OF COMPLIANCE

The trial will be carried out in accordance with International Council on Harmonisation Good Clinical Practice (ICH GCP) and the following:

- United States (US) Code of Federal Regulations (CFR) applicable to clinical studies (45 CFR Part 46, 21 CFR Part 50, 21 CFR Part 56, 21 CFR Part 312, and/or 21 CFR Part 812)

National Institutes of Health (NIH)-funded investigators and clinical trial site staff who are responsible for the conduct, management, or oversight of NIH-funded clinical trials have completed Human Subjects Protection and ICH GCP Training.

The protocol, informed consent form(s), recruitment materials, and all participant materials will be submitted to the Institutional Review Board (IRB) for review and approval. Approval of both the protocol and the consent form must be obtained before any participant is enrolled. Any amendment to the protocol will require review and approval by the IRB before the changes are implemented to the study. In addition, all changes to the consent form will be IRB-approved; an IRB determination will be made regarding whether a new consent needs to be obtained from participants who provided consent, using a previously approved consent form.

1. INTRODUCTION

1.1 STUDY OBJECTIVES

1.1.1 Primary Objective:

- To determine the objective response rate (CR+PR) of single agent LY2606368, a second-generation Chk 1/2 inhibitor, in participants with BRCA mutation-associated breast and ovarian cancers, HGSOC and TNBC at low genetic risk.

1.1.2 Secondary Objectives:

- To determine the safety and toxicity of LY2606368 in pretreated participants.
- To determine the progression-free interval (PFI) of the response to LY2606368
- To investigate response and resistance mechanisms of LY2606368 in recurrent platinum-resistant HGSOC participants.

1.1.3 Exploratory Objectives:

- To determine biochemical changes in the DNA damage repair and cell cycle check point pathways in tumor and blood samples in response to treatment.
- To investigate potential predictive biomarkers to Chk1/2 inhibitor in participants.
- To confirm the efficacy of LY2606368 in recurrent HGSOC participants by independent central review.

1.2 Background and Rationale

Approximately 15% of high grade serous ovarian cancers (HGSOC) are deficient in homologous recombination (HR) DNA double strand break (DSB) repair due to germline *BRCA1/2* mutation (gBRCAm) [1]. Acquired defects in the HR pathway are found in another approximately 35%[2-4]. The BRCA-like behavior has been described based on clinical and molecular features that parallel with gBRCAm-associated cancers' characteristics. The major clinical BRCA-like behavior identified is susceptibility to platinums and other DNA damaging agents[5-7]. Some of the molecular events described in BRCA-like behavior include *BRCA1* promoter methylation, *Fanconi F* methylation, upregulation of *EMSY*, and loss or reduction of proteins necessary for HR, such as RAD51, ATM, ATR, and Chk2[4,8-11]. The BRCA-like phenotype requires *p53* mutation, which secondarily leads to abnormal cell cycle checkpoint function[12]. Emerging data also indicate that half of metastatic castrate-resistant prostate cancer (mCRPC) patients have p53 mutations and approximately 20% of them have alterations in genes involved in DNA damage repair pathways, such as *BRCA1*, *BRCA2* or *ATM*[13,14]. Further, Small and colleagues recently presented at 2015 ASCO that upregulation of genes involved in cell cycle checkpoints were identified in tumor samples from mCRPC patients who were resistant to abiraterone or enzalutamide, suggesting DNA damage repair and cell cycle pathways as potential therapeutic targets

Checkpoint kinases (Chk1/2) are major regulators of the cell cycle and are intimately associated with the cellular response to DNA damage and repair (**Figure 1**). When damaged DNA is present, Chk1 maintains genomic integrity by stalling cell cycle progression thereby providing time for the appropriate repair to occur[15]. Chk1 functions as the primary mediator of cell cycle arrest in tumors with mutant p53, such as HGSOC and triple negative breast cancer (TNBC)[16,17]. When Chk1 protein is depleted, more replication origins are activated than the replication apparatus can tolerate in early S phase, resulting in slowed and arrested DNA replication forks and DNA DSB[18]. In p53-deficient tumor cells, loss of Chk1 results in DNA damage in the absence of the G1/S and G2/M checkpoints. Cells then proceed prematurely into mitosis with incompletely replicated and broken chromosomes, arresting in prometaphase[19]. In addition, Chk1 phosphorylates multiple downstream targets that regulate DNA replication, chromosome alignment, spindle checkpoints, and exit from cytokinesis[20]. Cells deficient in Chk1 have increased spontaneous chromosome missegregation. These cells lose the ability to recruit spindle checkpoint protein BubR1 to kinetochores and fail to activate the spindle checkpoint in response to misaligned chromosomes[20]. This finding suggests that Chk1 is essential for stable attachment of mitotic spindles to metaphase chromosomes. Thus, it is postulated that effective Chk1 inhibition alone can generate DNA damage and mitotic catastrophe. These functions of Chk1 indicate that its inhibition should negatively affect the viability of tumor cells, even in the absence of DNA damage by external agents.

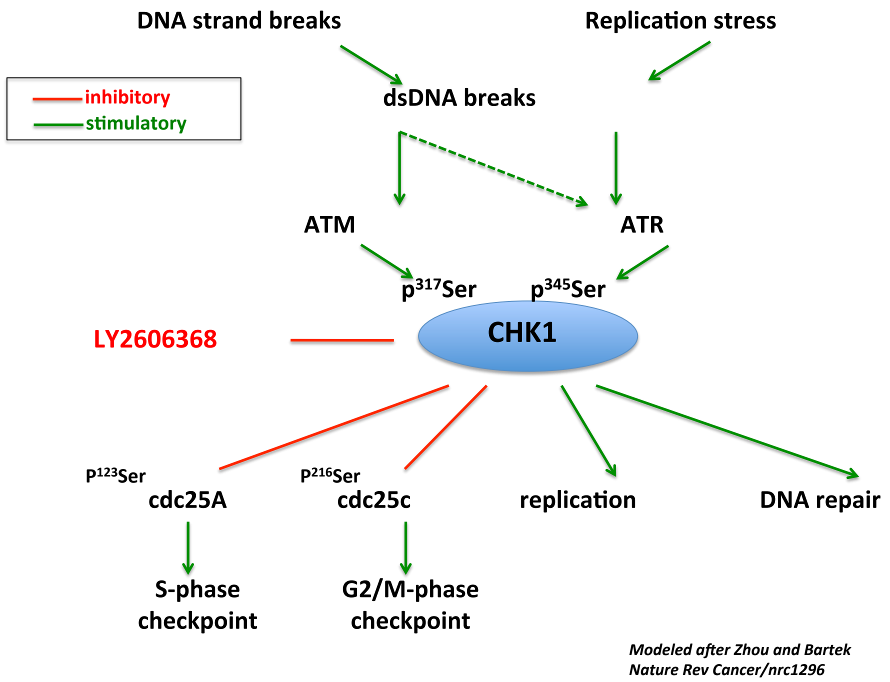

Figure 1: Homologous Recombination Pathway: Role of Chk1

<u>Figure 1</u>

<u>BRCA1/2 and Chk1/2 pathway interactions</u>

The concept of synthetic lethality has been applied to preclinical and clinical models that are deficient in a DNA damage repair pathway[21-23]. Following DNA damage, BRCA1 is involved in the control of cell cycle checkpoints[24], which represents another potential reason to target BRCA1 and Chk1/2 therapeutically. Chk1 is activated by ATR in response to stressors such as replication stress, chemotherapeutic agents, and single strand breaks (SSBs); whereas, Chk2 is activated by ATM in response to ionizing radiation, chemotherapeutics, or DSBs[25]. Activation of Chk1/2 leads to arrest at different phases of the cell cycle depending on the specific kinase activated, allowing DNA repair to occur. Yarden et al[26] demonstrated BRCA1 regulates the expression, phosphorylation and cellular localization of Chk1. The presence of phosphorylated BRCA1 affects the expression and localization of Cdc25C, a downstream target of Chk1[26].

Mateo *et al* recently presented preliminary findings from a phase II trial of olaparib monotherapy in mCRPC at 2015 AACR. All patients with mCPRC had been treated previously with docetaxel; 48/50 patients (96%) had been previously treated with abiraterone, and 20 patients (58%) with cabazitaxel. Sixteen of 49 evaluable patients experienced objective response with a RR of 32.7%. Furthermore, homozygous deletions and/or deleterious mutations in DNA repair genes were identified in 30.6% of evaluable patients (15/49), with the majority in *BRCA2* and *ATM* mutations. Among these fifteen patients, 13 (86.7%) responded to olaparib, suggesting targeting DNA repair in mCRPC offers novel direction of treatment in heavily pre-treated mCRPC patients.

Inhibitors of Chk1/2 abrogate normal cell cycle arrest, thereby preventing the repair of DNA damage. Altering the function of the checkpoint kinases may directly or indirectly impact BRCA1 function and thus may be a suitable target for therapy in gBRCAm-associated breast or ovarian cancers, and tumors with underlying DNA repair and cell cycle check points dysfunction. In this regard, patients with gBRCAm have an inherent defect in DNA damage response, which should predispose them to the synergistic effect of Chk1 inhibition leading to cell cycle progression, crisis and cell death. Based upon these mechanistic considerations and our early clinical activity findings from a current study, we believe it is reasonable to test clinical activity of Chk1/2 inhibitor on gBRCAm-associated and tumors with BRCA-like phenotype.

<u>LY2606368, a second-generation Chk1/2 inhibitor</u>

Preclinical data from Lilly indicates LY2606368 behaves mechanistically as a DNA-damaging agent, a cell cycle checkpoint 1/2-inhibitor, and a cell-cycle disruptor when used in vitro as a single agent, in ovarian (Ov) and breast (Br) cancer (Ca) cell lines (LY Investigator's Brochure [IB] 2016).

Preclinical Efficacy Pharmacology Summary

LY2606368 is a second generation, potent ATP-competitive inhibitor of Chk1 and Chk2. LY2606368 inhibits the activity of Chk1 with an IC_{50} of 1 nM and an inhibition binding constant (K_i) of 0.5 nM in an in vitro enzyme assay. Chk2 and the p90S6 kinases (Rsk) were inhibited by LY2606368 at an IC_{50} of less than 10 nM. Further, LY2606368 yielded single agent cytotoxicity with submicromolar concentration in BrCa cell lines including BRCA mutated BrCa and TNBC lines (**Figure 2**). LY2606368 has been shown to inhibit tumor growth when used either as monotherapy or in combination with other cytotoxic agents. The single agent activity of LY2606368 is related to the ability of this molecule to potently inhibit Chk1 thereby effectively interfering with its function to support normal DNA replication. This occurs via regulation of origin firing and/or aspects of chromosomal dynamics during mitosis that are thought to be dependent upon Chk1 kinase activity.

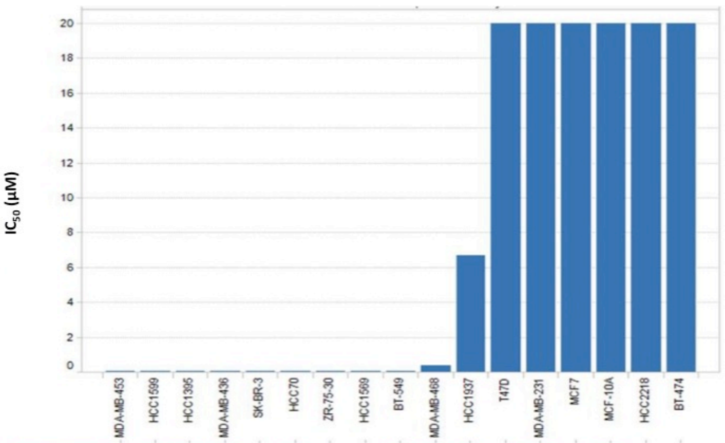

	MDA-MB-453	HCC1599	HCC1395	MDA-MB-436	SK-BR-3	HCC70	ZR-75-30	HCC1569	BT-549	MDA-MB-468	HCC1937	T47D	MDA-MB-231	MCF7	MCF-10A	HCC2218	BT-474
TNC		+	+	+		+			+	+	+		+		+		
HER2	+				+		+	+								+	+
ER							+					+		+		+	+
BRCA1/2		+	+	+			+	+			+						+
DSB Gene†	+			+				+		+							+

Figure 2: IC_{50} (the geometric mean of triplicate samples) of LY2940930 indicating potent single agent cytotoxic activity in various breast cancer cell lines. TNC: triple negative breast cancer, + DSB: double-stranded break

The preclinical studies in mouse models showed single agent activity of LY2606368 as monotherapy in ovarian tumor xenografts[27] (**Figure 3** and **Figure 4**). Treatment of tumor-bearing animals with LY2606368 alone on a 3 day per week for 3 weeks treatment schedule resulted in profound growth inhibition such that greater than 80% inhibition was observed at doses of 12mg/kg in both subcutaneous and orthotopic models. Moreover, the studies in the orthotopic SKOV3 OvCa model (BRCA1/2 wild type) demonstrated that treatment with LY2606368 not only robustly inhibited growth of the primary tumor but also significantly reduced the incidence of metastases and accumulation of ascites fluid (**Figure 5**). Overall, these animal studies indicate the potential therapeutic benefit of LY2606368 monotherapy in OvCa.

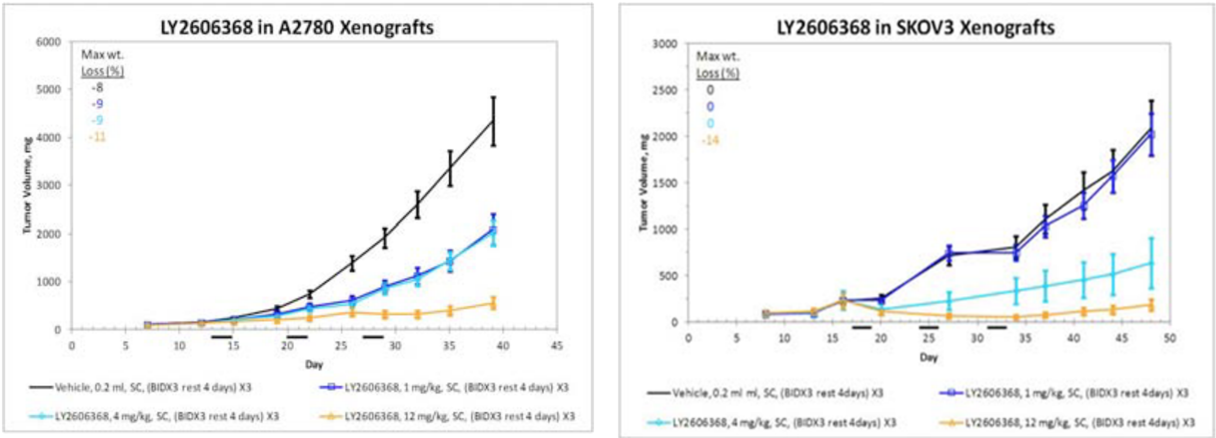

Figure 3: **Growth inhibition of A2780 and SKOV3 subcutaneous xenografts** Mouse bearing subcutaneous [13] A2780 OvCa xenografts (A) and SKOV3 OvCa xenografts (B) were treated with either 0.2mL of vehicle or LY2606368 SQ BID (1, 4, or 12mg/kg) for 3 days per week for 3 cycles beginning on day 13 (A) and on day 17 (B) after tumor cell implantation.

The table in the upper left corner of the tumor volume graph provides information regarding the maximum change in body weight (% relative to day 7 for A2780, and % relative to day 8 for SKOV3) observed during the study.

Figure 4

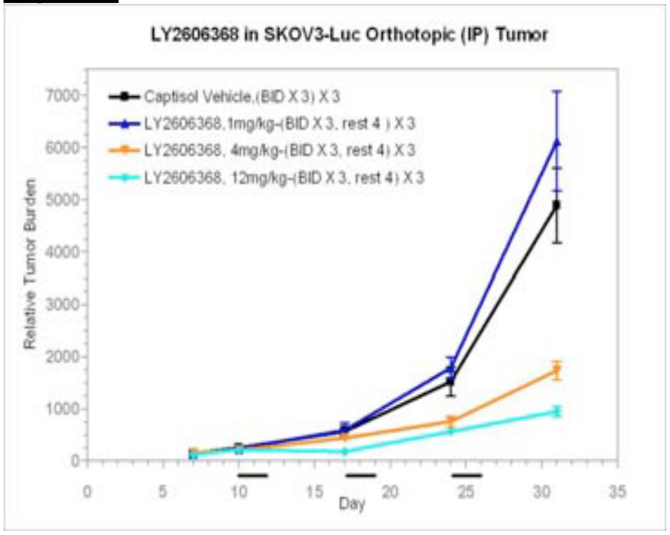

Figure 4: LY 260368 inhibits growth of SKOV3 tumor cells implanted IP. Mice bearing intraperitoneal SKOV3-Luc tumors were treated with either 0.2mL of vehicle or LY2606368 SQ BID (1, 4, or 12mg/kg) for 3 days per week for 3 weeks. The bold black bars along the x-axis depict the timing of dosing. Tumor burden was determined at weekly intervals using optical luminescence as a surrogate measure of tumor volume.

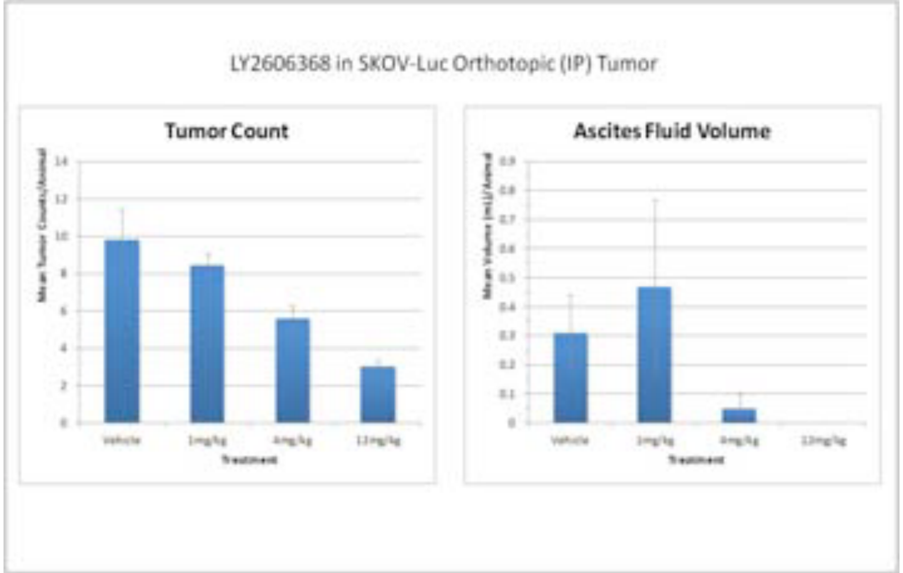

Figure 5: **LY2606368 reduces IP tumor counts and ascites fluid volume.** Mice bearing IP SKOV3-Luc tumors were treated with either 0.2 mL of vehicle or LY2606368 (1, 4, or 12mg/kg for 3 weeks (3 days per week) SQ BID. Tumor counts (left) and ascites volume (right) were determined at necropsy.

Identification of a gene signature highly represented in TNBC

Patients with BRCA mutation-associated and BRCA-like ovarian and/or breast cancers consist of approximately 50% of HGSOC[3,4] and 20% of TNBC[28], respectively, for a minimum estimate of approximately 14,000 at-risk women per year. High-throughput gene expression studies have identified gene signatures characteristic of TNBC subtypes. These studies have shed important insights regarding the molecular architecture of TNBC. Among these findings, dysregulation of the tumor suppressor p53 and Rb pathways is found in the majority of TNBC samples leading to alterations in the expression of genes involved in proliferation, DNA synthesis, DNA damage

and repair, cell cycle regulation, mitosis and apoptosis. Deeb and colleagues reported a core p53/Rb gene signature (Tag signature) that was highly expressed in a genetic mouse model of TNBC, but did not identify ER+ tumors, and which was prognostic of poor outcome[29]. This gene signature consists of approximately 150 genes highly enriched for DNA synthesis, DNA proliferation, cell-cycle, mitosis and apoptosis regulatory genes. This signature was also highly enriched in gene expression profiles of TNBC cells lines as reported by Neve et al[30]. Based upon this analysis, human TNBC cells and a murine TNBC cell line were used to identify potential therapeutic targets based upon the Tag signature. A custom siRNA library screen was used to determine which of the TNBC-Tag signature genes are critical for growth or survival of TNBC cells. Among these signature genes were Chk1/2 and ribonucleotide reductases 1 and 2 (RRM1 and RRM2; **Figure 6**)[31]. Chk1 has been reported to be highly expressed in TNBC. Further validation studies confirmed that siRNAs that inhibited Chk1 also yielded cytotoxicity in TNBC cells[31].

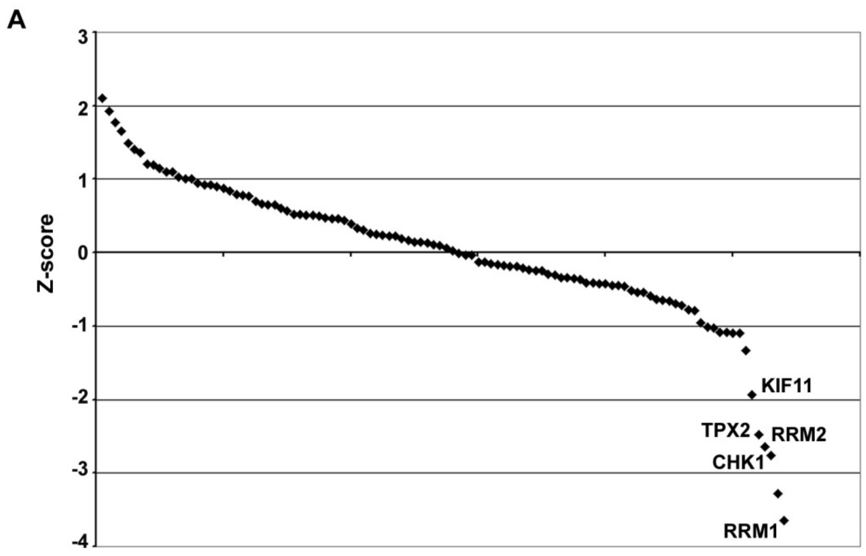

Figure 6: A custom siRNA library screen showed Chk1 and ribonucleotide reductases 1 and 2 (RRM1 and RRM2) are critical for growth and survival in TNBC.

LY2606368, a Chk1/2 inhibitor preliminary clinical data (2016 IB)

As of 04 April 2016, there are 4 Eli Lilly-sponsored and 1 investigator-initiated studies assessing LY2606368. 210 patients have been treated with LY2606368.

- The first-in-human Phase 1 study, Study I4D-MC-JTJA (JTJA), evaluated LY2606368 as a monotherapy in patients with advanced cancer.
- Study I4D-MC-JTJF (JTJF) is evaluating LY2606368 in combination with chemotherapy or targeted agents in patients with advanced cancer.
- Study I4D-JE-JTJK (JTJK) is evaluating LY2606368 monotherapy in Japanese patients with advanced cancer.
- Study I4D-MC-JTJI (JTJI) is evaluating LY2606368 in combination with chemotherapy

and radiation in patients with locally advanced head and neck cancer.

- Our study (14-C-0156) is the investigator-imitated phase 2 study of LY2606368 monotherapy in patients with breast, ovarian, or prostate cancer (NCT0220351). Effective with Amendment I (version date 4/24/2017), mCRPC cohort 4 was closed to accrual.

Toxicity and dose

The first-in-human Phase 1 study (study JTJA) evaluated 2 schedules (Schedule 1; Day 1 - 3 every 14 days, and Schedule 2: Day 1 every 14 days) in patients with advanced cancers (Part A). In addition, Part B consisted of 2 cohorts, B1 (squamous cell cancer of the head and neck) and B2 (squamous cell cancer of all tumor types) using Schedule 2 (**Table 1**). Part C consisted of the group of squamous cell cancer patients focused on squamous cell of the head and neck, squamous non-small cell lung cancer and squamous cell cancer of anus.

Table 1. Dose Levels (DLs): a phase 1 study of LY2606368 in advanced cancer patients

Schedule	Population	Cohort	Dose (mg/m^2)
**1: days 1-3 q 2wk**	**Advanced solid tumors**	**A1<br>A2<br>A3<br>A4<br>A5<br>A6<br>A8**	**10<br>12<br>15<br>20<br>30<br>40<br>50**
**2: day 1 q2wk**	**Advanced solid tumors**	**A7<br>A9<br>A10<br>A11<br>A12<br>A13**	**40<br>60<br>80<br>105<br>130<br>120**
**2: day 1 q2wk**	**SCCHN<br>SCC regardless of anatomical site**	**B1<br>B2**	**105<br>105**
**2: day 1 q2wk**	**SCCHN<br>Non-small cell lung cancer (squamous histology)<br>Squamous cell cancer of anus**	**C1<br>C2<br>C3**	**105<br>105<br>105**

SCCHN-squamous cell cancer (SCC) of head and neck

Dose limiting toxicities were defined in study JTJA and are limited to events occurring in cycle 1 of Part A that were possibly related to LY2606368 treatment (**Table 2**).

Table 2. Dose limiting toxicities in Part A of study JTJA

Event	Schedule	Dose (mg/m^2)
Grade 3 thrombocytopenia with bleeding	1	30
Grade 4 neutropenia lasting for >5 days	1	50
Grade 4 neutropenia and leukopenia lasting for >5 days and febrile neutropenia	1	50
Grade 4 neutropenia and thrombocytopenia lasting for >5 days	2	130
Grade 4 leukopenia and neutropenia lasting for >5 days	2	130
Grade 4 leukopenia and neutropenia lasting for >5 days	2	120
Grade 4 leukopenia, thrombocytopenia, and neutropenia lasting for >5 days	2	120

The maximum tolerated dose (MTD) of Schedule 1 was determined to be 40 mg/m^2 and the MTD of Schedule 2 was determined to be 105 mg/m^2. The schedule 2 MTD was selected as the recommended Phase 2 dose (RP2D) for LY2606368 monotherapy based on
1) Clinical PK data demonstrating potentially efficacious LY2606368 systemic exposure ranges; clinical PK data (**Table 4**) coincided with the predicted AUC (0-72) range (1008 to 3533 ng•hr/mL; median predicted AUC[0-72] = 1896 ng•hr/mL) to achieve the maximal tumor response after LY2606368 monotherapy in human,
2) Acceptable safety profile for each schedule, and
3) Increased patient convenience of fewer days of dosing.

The most common serious adverse events (SAE)s related to LY2606368 monotherapy (schedule 2: 105mg/m^2 every 14 days) were hematologic toxicities or complications resulting from neutropenia, leukopenia, or thrombocytopenia. Neutropenia was the most frequent toxicity and was observed at all doses and schedules of LY2606368. Overall, 89% of patients experienced neutropenia and/or leukopenia deemed related to LY2606368 treatment, with 71% grade 4 neutropenia. In general, neutrophil counts nadir on day 8 and grade 4 neutropenia was often transient (< 5 days). Febrile neutropenia was reported in 11% of patients, and no patients have discontinued or died because of febrile neutropenia. Patients who have grade 4 neutropenia or febrile neutropenia received (peg)filgrastim according to 2006 ASCO G-CSF guideline[32]. Related grade 4 thrombocytopenia has been observed in 6% of patients and two patients (2%) had clinically significant bleeding (epistaxis and hematochezia) in the setting of thrombocytopenia. Other hematologic adverse events (AEs) included anemia (49% [all grades]; 19% [grade 3/4]) and thrombocytopenia (46% [all grades]; 22% [grade 3/4]). Two patients (2%) had clinically significant bleeding with thrombocytopenia. Non-hematologic toxicity occurred at a lower frequency and was predominantly grade 1 or 2. Fatigue, nausea and headache are the only non-hematologic AEs that are related to study drug treatment occurring in >10% of patients.

Pharmacokinetics
Interim PK data were available from study JTJA including 27 patients across the dose range of 10 to 50 mg/m^2 on Schedule 1 (days 1-3 q 14 days), 18 patients across the dose range of 40 to 130 mg/m^2 on Schedule 2 from Part A, 31 patients in Part B (day 1 q 14 days), and 70 patients in

Part C (day 1 q 14 days). LY2606368 exposure increased in a dose-dependent manner from 10 to 130 mg/m^2 on day 1 of cycle 1 across both schedules of administration. The systemic clearance (CL) and volume of distribution at steady state (V_{ss}) were consistent after repeat administration in Parts B and C, suggesting time-independent PK behavior. A moderate-to-large degree of interpatient PK variability (percentage coefficient of variation [CV%]) in LY2606368 systemic CL was also observed (**Table 3**).

Table 3. Pharmacokinetic Summary from Parts B and C of study JTJA (RP2D 105mg/m^2 every 2 weeks schedule; source: 2016 IB)

	Schedule 2 (Cohorts B1 and B2 combined)		Schedule 2 (Cohorts C1, C2, and C3 combined)	
	Geometric Mean (CV%)			
	105 mg/m^2			
	Cycle 1 Day 1	Cycle 2 Day 1	Cycle 1 Day 1	Cycle 2 Day 1
N	26	26	66	56
C_{max} (ng/mL)	694 (57)	651 (58)	657 (52)	610 (47)
$C_{av,72}$ (ng/mL)	25.1 (39)[a]	25.8 (39)[b]	25.9 (48)[c]	26.1 (43)[d]
$AUC_{(0-\infty)}$ (ng·hr/mL)	1920 (42)[a]	1950 (42)[b]	1930 (51)[c]	1930 (46)[d]
$AUC_{(0-72)}$ (ng·hr/mL)	1810 (39)[a]	1860 (39)[b]	1870 (48)[c]	1880 (43)[d]
$AUC_{(0-24)}$ (ng·hr/mL)	1530 (40)[a]	1580 (40)[b]	1650 (45)[c]	1680 (38)[e]
$AUC_{(tlast-\infty)}$ (%)	4 (69)[a]	4 (65)[b]	5 (79)[c]	5 (74)[d]
CL (L/hr)	102 (46)[a]	98.7 (47)[b]	98.9 (54)[c]	100 (49)[d]
V_{ss} (L)	1480 (67)[a]	1280 (71)[b]	983 (67)[c]	909 (60)[d]
$t_{1/2}$ (hr)	19.2 (67)[a]	16.0 (60)[b]	9.95 (76)[c]	8.78 (74)[d]
R_A[f]	NC	1.02 (33)[g]	NC	1.08 (35)[h]

Abbreviations: $AUC_{(0-\infty)}$ = area under the plasma concentration-time curve from time zero to infinity; $AUC_{(0-24)}$ = area under the plasma concentration-time curve from time zero to 24 hours; $AUC_{(0-72)}$ = area under the plasma concentration-time curve from time zero to 72 hours; $AUC_{(tlast-\infty)}$ = $AUC_{(0-\infty)}$ extrapolated beyond the last measurable plasma concentration; $C_{av,72}$ = average plasma concentration over 72 hours after prexasertib infusion; CL = systemic clearance; C_{max} = maximum plasma concentration; CV% = percent coefficient of variation; NC = not calculated; RA = intercycle accumulation ratio; $t_{1/2}$ = half-life; V_{ss} = volume of distribution at steady state.

[a] N = 25, [b]N = 24, [c]N = 64, [d]N = 49, [e]N = 50, [f]RA = (Cycle 2, Day 1 $AUC_{[0-24]}$/Cycle 1, Day 1 $AUC_{[0-24]}$]), [g]N = 20, [h]N = 45.

Furthermore, 44% of the noncompartmental $AUC_{(0-72)}$ values calculated were equal to or greater than the median $AUC_{(0-72)}$ (1896 ng*hr/mL; based on the Calu-6 xenograft PK/PD model to achieve the maximal tumor response in humans) predicted to correlate with the the maximal tumor response after LY2606368 monotherapy, while 95% of the $AUC_{(0-72)}$ values calculated were greater than the lower end of the 90% PI (1008 ng*hr/mL) for maximum tumor response.

A preliminary population PK analysis is available from study JTJA (n=146). There is low-to-moderate degree of interpatient variability across PK parameters of the model (CV% range: 23-51%). The population PK model also predicted that LY2606368 exposure following administration of 105mg/m^2 ($AUC_{(0-72)}$ of 2112 ng*hr/mL; 90% PI: 1179-3434 ng*hr/mL) is greater than the $AUC_{(0-72)}$ noncompartmental (model independent) determined values (**Table 3**).

In addition, the population PK analysis supports a body surface area (BSA)-based dose regimen is appropriate for LY2606368 administration. The BSA distribution based on specific noncompartmental PK parameters (C_{max} and $AUC_{(0-72)}$) showed overlap of baseline BSA values across the 4 exposure quartiles for C_{max} and $AUC_{(0-72)}$, and similar BSA distributions across each exposure quartile following administration of 105mg/m^2 (**Figure 7**).

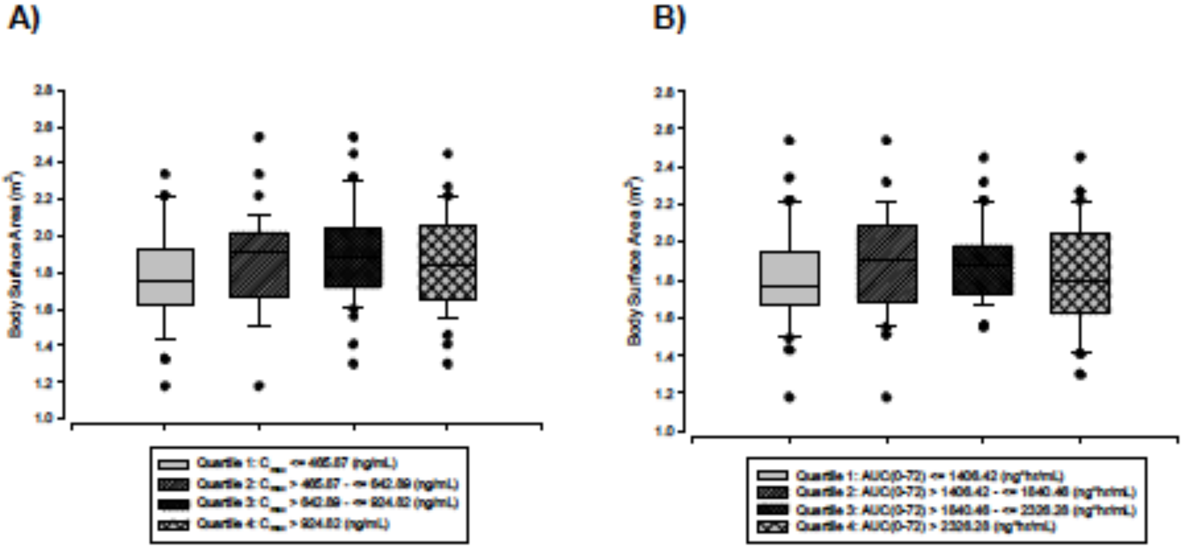

Figure 7. Box plots of baseline BSA stratified by (A) observed C_{max} and by (B) calculated noncompartmental $AUC_{(0-72)}$, values following LY2606368 at 105mg/m^2 administration (study JTJA parts B and C)

Clinical drug interactions

The potential drug-drug interaction was evaluated in human liver microsomes. In vitro model showed LY2606368 inhibited CYP1A2 (K_i =1.6 uM =767 ng/mL) and CYP2D6 (K_i =2.8 uM =1343 ng/mL), but showed no inhibition of CYP2C8, CYP2C9, CYP2C19, CYP2B6, or CYP3A4 over a range of concentration of 0.103 to 25 uM. However, in simulations of human PK models, the increase in CYP2D6 substrate AUC and C_{max} ratios were <1.2 for both substrates at RP2D (105mg/m^2) of LY2606368 (See 2016 IB for further information). Overall, the data suggest the potential for a clinical drug-drug interaction is minimal.

Preliminary efficacy data

In part A (dose escalation) of study JTJA, two of the 45 patients (4.4%) had PR: one patient with anal squamous cell cancer (SCC) and one had SCC of the head and neck. Fifteen of 45 patients (33%) achieved RECIST SD, ranging 1.2 to 6.7 months, six of whom had SCC.

Preliminary activity of LY2606368 in HGSOC (NCI study 14-C-0156; NCT02203513)

Platinum-resistant recurrent HGSOC

Patients with high grade serous ovarian cancer (HGSOC) often are initially responsive to platinum-based chemotherapy and then become increasingly resistant with more lines of therapy [33]. However, once a patient is platinum resistant or refractory there is not a consensus regarding which therapeutic agent is preferred. Pegylated liposomal doxorubicin (PLD), gemcitabine, topotecan, taxanes, and etoposide are all commonly used in this setting. In platinum-resistant patients who had received 1 to 2 previous lines of therapy, bevacizumab when added to paclitaxel, PLD, or topotecan chemotherapy increased the overall response rate (ORR) from 12% to 27% [34]. Agents that are approved by the U.S. FDA for patients who have received at least 1 previous line of chemotherapy include topotecan, PLD, and bevacizumab in combination with paclitaxel, PLD, or topotecan. More recently, exploiting defects in homologous recombination repair pathways with PARP inhibitors has been shown to be an effective strategy for treating ovarian cancer. Olaparib obtained accelerated approval for patients with deleterious germline *BRCA* mutated advanced ovarian cancer after 3 or more lines of chemotherapy. The RR (n=137) was 34%, and median duration of response was 7.9 months (Kim et al. 2015). However, no agents are specifically approved for patients who do not harbor a *BRCA1/2* mutation and have had at least 2 lines of previous chemotherapy, and new treatment options are needed.

LY2606368 inhibits checkpoint kinase 1, a multifunctional protein kinase that controls DNA replication, mediates cell cycle arrest, and is essential for homologous recombination repair (McNeely et al. 2014). In phase 1 testing, durable objective clinical responses, including a CR, were observed [35]). The mechanism of action of LY2606368 is distinct from traditional cytotoxic agents, and it is anticipated that the mechanisms of sensitivity and resistance will also be different. This makes LY2606368 a potentially attractive agent for the treatment of patients with HGSOC whose disease has progressed after at least 2 lines of chemotherapy.

Preliminary clinical evidence of LY2606368 in heavily pretreated HGSOC

Preliminary results from 32 heavily pretreated HGSOC patients have been presented at the European Society of Medical Oncology meeting in October 2016, including 7 patients in Cohort 1 (patients with a documented *BRCA* mutation) and 25 patients in Cohort 2 (patients with a negative family history of hereditary breast ovarian cancer syndrome, or negative germline *BRCA* mutation test). Transient grade 4 neutropenia was observed in 69% of patients and resolved to less than or equal to grade 2 within 8 days after onset. Two patients (6%) had febrile neutropenia. Only one patient had dose reduced to 80mg/m^2. Other toxicities included decreased white blood cell count (78%), anemia (66%), and decreased platelet count (34%). Nonhematologic AEs were generally mild and included fatigue, nausea, vomiting, and diarrhea (9% each). One patient experienced Grade 3 diarrhea and vomiting during infusion.

Among 20 evaluable patients in HGSOC (Cohort 2), there were 7 confirmed PRs, for an ORR of 7 of 20 (35%). Patients had a median of 5 previous therapies (range: 1 to 13). Of the 20 evaluable patients, 15 had platinum-resistant or platinum-refractory disease. Five of 15 patients had a PR for an ORR of 33%. The interim median duration of response is 6 months (3+ to 9 months). The numbers of previous therapies for the responders were 2, 4, 5, 6, and 13. An additional 5 patients with platinum-resistant or platinum-refractory disease had SD ≥4 months for an overall clinical benefit rate of 66% (10 of 15). Six of 10 patients who had a PR or SD ≥4 months had received previous bevacizumab. Notably, an expected RR for this patient population with existing agents is approximately 15% (unpublished meta-analysis conducted by Eli Lilly in

2 lines+ ovarian cancer) . None of the 6 evaluable patients in Cohort 1 achieved CR or PR, but 4 (67%) achieved SD lasting at least 4 months, for a DCR of 67%. However, our *BRCA* mutant patient cohort, in which all patients received prior PARP inhibitors, did not show response to LY2606368, suggesting CHK1/2 inhibition alone may not reverse the observed PARP inhibitor resistance. **Figure 8** presents a plot of best responses for target lesions by patient, among patients treated with LY2606368.

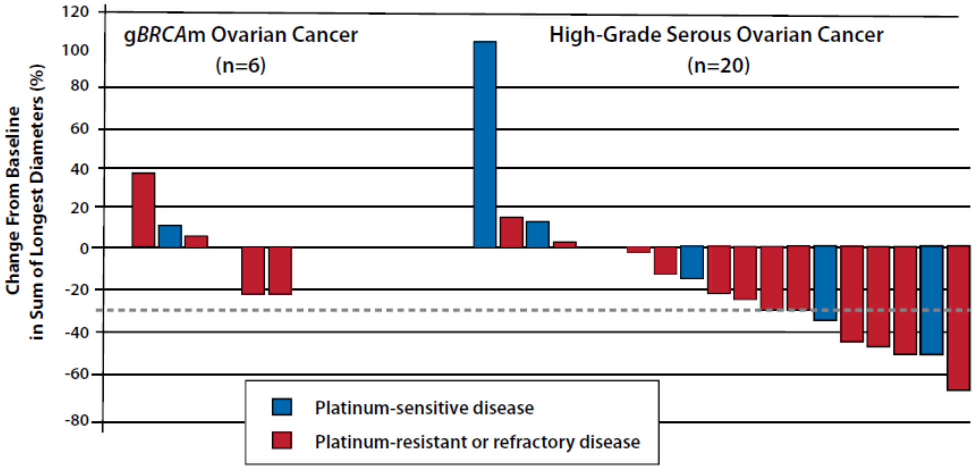

Abbreviation: *gBRCA*m = germline *BRCA* mutated.

Figure 8. Best response for target lesions by patient, among patients treated with LY2606368

The emerging data for LY2606368 suggest that this compound may demonstrate substantial improvement in heavily pretreated HGSOC patients with BRCA wild type on the basis of the clinically significant endpoint of RR. In particular, the preliminary data in patients with platinum-resistant disease, an area of high unmet need, are favorable. There are currently no agents approved by the FDA for patients with platinum-resistant disease without germline *BRCA* mutation who have progressed after at least 2 previous chemotherapy regimens. Although the LY2606368 data are limited, they are superior to historical response rates. *For this reason, we are opening the new cohort 6 for BRCA wild type platinum-resistant recurrent HGSOC - the same patient population as cohort 5 but without biopsiable disease - to facilitate acquiring efficacy data of LY2606368 prior to launching a randomized phase 2/3 trial.*

In addition, we recently opened the new cohort 5 for *BRCA* wild type patients with platinum-resistant recurrent HGSOC with mandatory paired biopsies (baseline and at progression) to study potential response/resistance mechanisms and also to confirm the preliminary monotherapy activity. LY2606368 is clinically active, but not all tumors respond and most actually progress while on therapy. Clinical activity to date is associated with the *BRCA* wild type subpopulation, but this may be a correlate; resistance may involve interactions with the PARP pathway, NBS1 in DNA repair (unpublished data, Murai and Pommier, DTB/CCR) and likely other as yet unknown pathways. Development of agents that target cell cycle and DNA damage repair is critical given ~50% of HGSOCs have defects in these pathways. Based on our preliminary

studies, the hypothesis to be tested in this cohort 5 is that Chk1/2 inhibition may have novel resistance mechanism(s) such as NBS1 deficiency in a subset of HGSOCs. By comparing tumor samples from pre-LY2606368 and at progression, and between responders and non-responder on clinical trial, we expect to identify novel targets for HGSOC patients, delineate mechanisms of Chk1/2 inhibition resistance and identify biomarkers predictive of response to Chk1/2 inhibition, which will ultimately assist in stratifying patients for these therapies.

Summary

Understanding more about the interaction of cell cycle checkpoint inhibition in BRCA mutation-associated and/or BRCA-like breast or ovarian cancers, exploring novel therapeutic trial strategies, and defining potential predictive biomarkers of response or resistance, are critical to advance therapeutic directions in these important but rare subsets of women's malignancies. We hypothesized that therapeutic inhibition of cell cycle check point and DNA damage repair functions will yield benefit in patients with BRCA mutation-associated breast or ovarian cancer, HGSOC, and TNBC at low genetic risk. Additionally, genomic studies have revealed clinically actionable alterations in DNA repair pathways, including p53 mutation in mCRPC. These aberrations are therapeutic targets in both BRCA mutation and BRCAwt disease. Chk1/2 inhibition is a novel treatment strategy in mCRPC where other treatment options become limited over time. In order to evaluate cell cycle inhibition in mCRPC, a subset of patients with soft tissue disease independent of BRCA mutation status will be enrolled (Cohort 4). We hypothesized therapeutic inhibition of Chk1 cell cycle and DNA damage repair functions will yield benefit in patients with pretreated mCPRC. A positive signal in this pilot single arm study will lead to discussions of appropriate next steps (e.g. RP2) or combination with other DNA repair inhibitor(s) to broaden evaluation of this agent. Additionally, effective with amendment I, the Cohort 4 (mCRPC) was closed due to slow accrual.

2 ELIGIBILITY ASSESSMENT AND ENROLLMENT

2.1 ELIGIBILITY CRITERIA

2.1.1 Inclusion Criteria

2.1.1.1 A documented deleterious germline BRCA1/2 mutation (gBRCA1/2m) obtained in a CLIA-certified laboratory, including but not limited to Myriad Genetics, either by multi-gene panels or individual testing, for Cohort 1 participants prior to study enrollment. Participants with documented somatic BRCA mutation obtained in a CLIA-certified laboratory also will be considered for Cohort 1. Variants of uncertain significance (VUS) of BRCA1/2 are not considered deleterious. Participants with VUS or

deleterious mutation in other genes without BRCA mutation can be considered for Cohort 2 or 3 or 5.

2.1.1.2 Participants enrolling in the sporadic high grade serous epithelial or high grade endometrioid ovarian cancer group, Cohort 2, must have a negative family history of hereditary breast ovarian cancer (HBOC) syndrome, or negative BRCA1/2 mutation test.

2.1.1.3 Participants enrolling in the triple negative breast cancer (ER-/PR-/Her2-) group, Cohort 3, must have a negative family history of HBOC syndrome, or negative BRCA1/2 mutation test. A family history of HBOC is defined by NCCN Genetic/Familial High-Risk Assessment: Breast and Ovarian guideline (**Appendix D**)

2.1.1.4 For Cohorts 1-3, 5 and 6: participants must have breast and/or epithelial or endometrioid ovarian cancer, primary peritoneal cancer, and/or fallopian tube cancer histologically or cytologically confirmed at the NCI that is metastatic or unresectable and for which standard curative measures do not exist or are no longer effective. ER/PR/HER2 status needs to be documented either by an outside source or at NCI. Participants with BRCA1/2 mutation with history of or active breast and ovarian cancers are considered for Cohort 1. Those without gBRCA1/2m or somatic BRCA mutation will follow Exclusion Criteria **2.1.2.10**.

2.1.1.5 Participants enrolling in Cohort 5, the recurrent platinum-resistant sporadic high grade serous epithelial or high grade endometrioid ovarian cancer group, must have a negative family history of HBOC syndrome, or negative BRCA1/2 mutation test. Participants should have recurrent platinum-resistant - defined as disease recurrence by imaging within 6 months of the last receipt of platinum-based chemotherapy. Rising CA125 only is not considered as platinum-resistant disease. Participants with primary platinum refractory disease defined as progression during or within 3 months after receiving first-line platinum based chemotherapy are not eligible.

2.1.1.6 All participants must have measurable disease, defined as at least one lesion that can be accurately measured in at least one dimension (longest diameter to be recorded for non-nodal lesions and short axis for nodal lesions) as $\geq$20 mm with conventional techniques or as $\geq$10 mm with spiral CT scan. See Section **6.2** for the evaluation of measurable disease.

2.1.1.7 All participants except Cohort 6 must have at least one lesion deemed safe to biopsy and be willing to undergo a mandatory

baseline biopsy. For Cohort 5, the second biopsy at progression is mandatory for the responders (PR/CR/SD) $\geq$ 4 months.

2.1.1.8 Participants enrolling in Cohort 6, the recurrent platinum-resistant sporadic high grade serous epithelial or high grade endometrioid ovarian cancer group, must have a negative family history of HBOC syndrome, or negative BRCA1/2 mutation test. Participants should have recurrent platinum-resistant, defined as disease recurrence by imaging within 6 months of the *last* receipt of platinum-based chemotherapy. This cohort should have measurable (defined by RECIST v1.1) but without biopsiable disease, determined by PI and Interventional Radiology (e.g., cystic abnormal mass, not safely biopsiable disease). Rising CA125 only is not considered as platinum-resistant disease. Participants with primary platinum refractory disease defined as progression during

or within 3 months after receiving *first-line* platinum based chemotherapy are not eligible.

2.1.1.9 Participants must be at least 4 weeks from previous therapy (chemotherapy, hormonal therapy, and radiation therapy, or investigational agents; 6 weeks for mitomycin C).

2.1.1.10 The use of raloxifene, denosumab, or bisphosphonates for bone health is allowed.

2.1.1.11 There is no limit on the number of prior therapies.

2.1.1.12 Participants must be at least 1 week from the last dose of complementary or alternative medications.

2.1.1.13 Participants who have had major surgery must be fully recovered and ≥4 weeks post-operative prior to enrolling on study.

2.1.1.14 Age ≥18 years.

2.1.1.15 ECOG performance status ≤2.

2.1.1.16 Participants must have normal organ and marrow function (in the absence of transfusion 24 hours prior to dosing) as defined below:

– leukocytes	≥3,000/mcL
– absolute neutrophil count	≥1,500/mcL
– platelets	≥100,000/mcL
– hemoglobin	≥10mg/dL
– total bilirubin	≤1.5 X institutional upper limit of normal
– AST(SGOT)/ALT(SGPT)	≤3 X institutional upper limit of normal
– creatinine	≤1.5 X institutional upper limit of normal
OR	
– measured creatinine clearance	≥45 mL/min/1.73 m^2 for participants with creatinine levels above institutional normal.

2.1.1.17 Potassium (K) should be within the range of ≥ 3.6 mEq/L.

2.1.1.18 Women of childbearing potential must have a negative urine or serum pregnancy test within 7 days prior to the start of the study.

2.1.1.19 The effects of LY2606368 on the developing human fetus are unknown. For this reason, all subjects of reproductive potential must agree to use adequate contraception prior to study entry, for the duration of study participation, and for at least four months following the last dose of experimental therapy. All subjects of reproductive potential must also agree to use both a barrier method and a second method of birth control during the course of the study and for four months after the last dose of study drug(s). Should a woman become pregnant or suspect she is pregnant while she is

participating in this study, she should inform her treating physician immediately.

2.1.1.20 Ability of subject to understand, adhere to protocol requirements and the willingness to sign a written informed consent document.

2.1.2 Exclusion Criteria

2.1.2.1 Participants who are receiving any other investigational agents.

2.1.2.2 Participants with known brain metastases should be excluded from this clinical trial because of their poor prognosis and because they often develop progressive neurologic dysfunction that would confound the evaluation of neurologic and other adverse events. Participants with brain metastases diagnosed greater than 1 year prior to study entry may be considered if they received sterilizing therapy to the CNS (resection or radiation) and have been CNS recurrence-free for the 1-year period.

2.1.2.3 Participants who have had prior treatment with LY2606368 or other Chk inhibitors

2.1.2.4 Participants with a serious cardiac condition, such as congestive heart failure; New York Heart Association Class III/IV heart disease; unstable angina pectoris; myocardial infarction within the last 3 months; valvulopathy that is severe, moderate, or deemed clinically significant despite medical intervention; or arrhythmias that are symptomatic or refractory to medical intervention.

2.1.2.5 Participants who have QTc interval of > 470 msec on a screening electrocardiogram.

2.1.2.6 Participants with a prior history of drug-induced serotonin syndrome, or a family history of long-QT syndrome.

2.1.2.7 Lack of recovery of prior adverse events due to prior cancer therapy to Grade ≤1 (NCI CTCAE; except alopecia). Electrolyte abnormalities that are corrected with supplementation will be eligible. Participants with platinum-related grade 2 or greater hypomagnesemia (on replacement) will be eligible. Stable persistent grade 2 peripheral neuropathy may be allowed as determined on a case-by-case basis at the discretion of the PI.

2.1.2.8 Uncontrolled intercurrent illness including, but not limited to, symptomatic congestive heart failure, unstable angina pectoris, cardiac arrhythmia, clinically significant GI bleeding or hemoptysis within 28 days prior to the start of the study, or psychiatric

illness/social situations that would limit compliance with study requirements.

2.1.2.9 Participants with active infection will not be eligible, but may become eligible once infection has resolved and they are at least 7 days from completion of antibiotics.

2.1.2.10 Another previous or current invasive malignancy within the last 2 years, with the exception of curatively treated stage Ia cervical carcinoma, or resected stage Ia endometrial cancer, and noninvasive nonmelanoma skin cancers. Participants with gBRCA1/2m and primary breast or ovarian cancers will be eligible for Cohort 1 (**2.1.1.4**).

2.1.2.11 HIV-positive participants on combination antiretroviral therapy are ineligible because of the potential for pharmacokinetic interactions with LY2606368. HIV- positive participants who are not on HAART and have CD4 counts > 500 will be considered on an individual basis.

2.2 Participant Registration and Status Update Procedures

Registration and status updates (e.g., when a participant is taken off protocol therapy and when a participant is taken off-study) will take place per CCR SOP ADCR-2, CCR Participant Registration & Status Updates found **here**.

2.3 Treatment Assignment and Randomization/Stratification Procedures

Cohorts

Number	Name	Description
1	BRCA mutation positive breast or ovarian cancer	Breast or ovarian cancer patients with a documented deleterious germline BRCA mutation (gBRCAm) or somatic BRCA mutation.
2	BRCA mutation negative ovarian cancer	High-grade serous ovarian cancer (HGSOC) patients with negative BRCA mutation testing or negative family history of hereditary breast and ovarian cancer syndrome
3	BRCA mutation negative triple negative breast cancer	Triple negative breast cancer patients with negative BRCA mutation testing or negative family history of hereditary breast and ovarian cancer syndrome
4	Prostate cancer	Effective with amendment I (version date 4/24/2017), metastatic castration-resistant prostate cancer cohort is closed.
5	BRCA mutation negative ovarian cancer, biopsy	High-grade serous ovarian cancer (HGSOC) patients with negative BRCA mutation testing or negative family history of hereditary breast and ovarian cancer syndrome, they must have recurrent platinum-resistant HGSOC with measurable and biopsiable disease.

6	BRCA mutation negative ovarian cancer, non-biopsy	High-grade serous ovarian cancer (HGSOC) patients with negative BRCA mutation testing or negative family history of hereditary breast and ovarian cancer syndrome, they must have platinum-resistant HGSOC with measurable but *without* biopsiable disease.

Arms

Number	Name	Description
1	Prexasertib	Prexasertib monotherapy treatment

Randomization/Stratification and Arm Assignment

Randomization procedures do not apply to this trial.

No stratification is planned for this study.

Participants in all Cohorts will be directly assigned to Arm 1.

2.4 SCREENING AND BASELINE EVALUATIONS

Evaluations required at baseline can be omitted if obtained at screening within 1 week of Cycle 1 Day 1. The baseline (enrollment) EKG may be used as pre-infusion EKG on Cycle 1, Day 1. The baseline CT scan of the chest, abdomen, and pelvis can be omitted if obtained at screening within 16 days of Cycle 1 Day 1.

2.4.1 Imaging Studies

Every participant must have a CT scan of chest, abdomen and/or pelvis prior to receiving treatment. PET/CT alone is not sufficient. These tests are deemed medically indicated to establish baseline disease characteristics. This must be completed within 16 days prior to enrollment. Participants with CT contrast hypersensitivity in the presence of appropriate pre-medication may be followed by MRI with gadolinium per investigator-approval. A contrast CT or MRI of brain may be requested if a participant has advanced breast cancer or has symptoms or physical examination findings that suggest a risk of brain metastases. In some participants, an MRI, PET, bone scans, or ultrasound may be appropriate to include in disease monitoring and may be included in addition to the baseline CT scan.

2.4.2 An EKG should be obtained within 1 week prior to enrollment.

2.4.3 Laboratory Evaluations [baseline is to be obtained within 1 week prior to enrollment].

- Hematological profile: CBC with differential and platelet count, PT/INR/PTT

- Biochemical profile:

    - Screening – Bilirubin, AST, ALT, creatinine or creatinine clearance
    - Baseline - 14 comprehensive metabolic panel (sodium, potassium, chloride, carbon dioxide, BUN, creatinine or creatinine clearance, glucose, AST, ALT, bilirubin, calcium, total protein, albumin, alkaline phosphatase), magnesium, urinalysis

- CA125 will be followed on all ovarian cancer and participants in whom it has been shown to be abnormal. CA125 will not be used in any participants for disease assessment decisions.
- Serum beta-hCG or urine pregnancy test in women of childbearing potential.

3 STUDY IMPLEMENTATION

3.1 STUDY DESIGN

This is an open label, non-randomized phase II pilot trial to examine activity of LY2606368 in participants in 6 independent cohorts:

1) Women with BRCA mutant breast or ovarian cancer
2) HGSOC at low genetic risk
3) TNBC at low genetic risk
4) mCRPC with or without BRCA mutation: Effective with Amendment I (version date 4/24/2017), Cohort 4 was closed to accrual.
5) Recurrent platinum-resistant HGSOC at low genetic risk
6) Recurrent platinum-resistant HGSOC at low genetic risk without biopsiable disease

Participants will be evaluated at baseline and prior to each cycle by history and physical examination (H&P). Laboratory studies will be performed prior to each drug administration. In cohorts 1 – 3, 5 and 6, participants will be evaluated every two cycles by imaging studies using RECIST criteria (CT scan or other as appropriate). In the mCRPC cohort, participants will be evaluated by imaging studies using RECIST criteria (CT scan and other imaging as appropriate) and technetium 99 bone scan[36] every 3 cycles. All participants in this cohort will have baseline CT and technetium 99 bone scan imaging. As per the Prostate Cancer Clinical Trials Working Group 2 recommendations, every effort should be made to keep participants on treatment with LY2606368 for at least 12 weeks[36] and thus for the purposes of this cohort, participants should be evaluated every 3 cycles. Effective with Amendment I (version date 4/24/2017), mCRPC cohort 4 was closed to accrual.

Participant adherence to drug treatment and reporting of adverse effects will be reviewed by history.

LY2606368 is administered intravenously once every 14-days in each 28-day cycle. Administration of LY2606368 may be delayed up to 7 days due to holidays, inclement weather, conflicts, or similar reasons. The timing of subsequent administrations is then adjusted to maintain a 14 days-interval. For safety reasons, LY2606368 may not be administered with less than a 14-days interval.

Table 4. Dose Levels of LY2606368

**Dose Level (DL)**	**LY2606368 IV q2w (mg/m^2)**
Starting Dose DL1	105
DL-1	80
DL-2	60

3.2 Drug Administration

See **Study Schema**:

The RP2 dose of 105 mg/m^2 LY2606368 administered by IV over 1 hour (+15 minutes) once every 14 days will be used. Administration of LY2606368 may be delayed up to 7 days due to holidays, inclement weather, conflicts, or similar reasons. The timing of subsequent administrations is then adjusted to maintain a 14 days-interval. For safety reasons, LY2606368 may not be administered with less than a 14-days interval.

Participants should avoid taking concomitant medications that are known or suspected to cause prolonged QTc or Torsades de Pointes (**Appendix B**) 12 hours before through 12 hours after administration of LY2606368 and, if possible, alternative agents should be considered.

Participants will continue therapy until documentation of progressive disease, limiting toxicity (**3.3**), intercurrent medical issues, or participant withdrawal. Participants with CR will discontinue therapy two cycles past a radiographically confirmed CR (see **6.2.4** for guidance), and will then be followed actively in clinic every 4 weeks with reassessment noninvasive imaging on an every 12 week schedule until progression or withdrawal. Participants with signs or symptoms of clinical progression may be imaged earlier in order to provide optimal care.

Palliative radiation therapy may be considered for symptom and/or pain control in consultation with Radiation Oncology, only for those who continue to have clinical benefit from LY2606368 treatment at the PI's discretion (see section **14.1.2**for guidance of concurrent radiation therapy and LY2606368).

Participants will be contacted over the phone or assessed in person at least once weekly during the first cycle of treatment. Subsequent follow up will be at least monthly, with the start of each new cycle, and then during follow up according to the defined schedule (see 3.4: Study Calendar).

Pre-cycle and off-treatment AE assessments will be done at a scheduled clinic visit. Off-study assessments will be performed within 30 days of stopping study treatment over the phone or at a clinic visit.

3.3 DOSE MODIFICATIONS

See **Table 4** Dose Levels.

Table 5: Treatment modifications of LY2606368 for hematologic toxicities *

Grade	*Occurrence*	*Immediate Action*	*Resumption of Therapy*
1	All	None	No interruption
2	All	None	No change in dose, but initiate appropriate medical therapy
3	1st **	Hold therapy until grade ≤2	Initiate appropriate medical therapy and no change in dose upon re-initiation if recovers to grade 2 or better within 3 weeks
	2nd**	Hold therapy until grade ≤2	Initiate appropriate medical therapy and then reduce LY2606368 to the next lower dose level upon re-initiation
4	All **	Hold therapy until grade ≤2	Initiate appropriate medical therapy and then reduce to the next lower dose level upon re-initiation

* For adverse events that are possible, probable, or definitely attributable to LY2606368 alone (attributions 3-5).
** Any event of temporary (≤ 7days) afebrile neutropenia (grade 3 or grade 4) will not require dose reduction or discontinuation of treatment.
** Grade 3 or 4 thrombocytopenia > 7 days or any thrombocytopenia requiring platelet transfusion for bleeding will result in dose reduction to the next lower dose level (see **3.3.1.2**).
** If the first event of grade 3 anemia is refractory to red cell transfusion and growth factor support within 3 weeks then LY2606368 should be dose reduced to the next lower dose level (see **3.3.1.3**).

**If no resolution occurs within the 3-week period, the study drug will be discontinued.

3.3.1 Hematologic Toxicity

Hold LY2606368 for participants with grade 3 or 4 hematologic toxicities until they recover to grade 2 or better. The participants at a high-risk for bleeding; e.g., concurrent use of anti-coagulation, may hold LY2606368 until platelets recover to grade 1 or better. Blood counts will be repeated every other day until resolution to grade 2 or better.

3.3.1.1 Neutropenia:

- Prophylactic and/or therapeutic growth factor support will be given following the ASCO practice guidelines[32].
- Participants who experience grade 3 or 4 neutropenia or fever during neutropenia and are not receiving growth factor support can be supplemented with filgrastim 5 mcg/kg/day until ANC is greater than 3,000/ml, or pegfilgrastim 6mg at the discretion of the team.
- If recurrent grade 3 or 4 neutropenia (> 7 days) is refractory to growth factor support, then LY2606368 should be dose reduced to the next lower dose level.
- A subsequent event of grade 4 neutropenia (> 7 days) or fever during neutropenia, while on therapeutic or prophylactic filgrastim growth factor support, will necessitate discontinuation of a study drug.
- Participants with ANC ≤1500/mL on day 1 of a cycle may receive filgrastim 5 mcg/kg for a period of 3-5 days or pegfilgrastim 6mg to avoid treatment delays and risk of dose reduction, if they did not receive growth factor support in the immediately preceding cycle.
- Prophylactic antibiotics may be considered for use in participants that have experienced febrile neutropenia on the study or participants deemed at higher risk for febrile neutropenia by the investigators.

3.3.1.2 Thrombocytopenia:

- Asymptomatic grade 3 or 4 thrombocytopenia lasting ≤ 7 days will not result in drug discontinuation.
- Grade 3 or 4 thrombocytopenia > 7 days or any thrombocytopenia requiring platelet transfusion for bleeding will result in dose reduction to the next lower dose level.
- The participants at a high-risk bleeding, e.g., concurrent use of anti-coagulation may hold LY2606368 until platelets recover to grade 1 or better ($\geq$75,000/mm^3).

3.3.1.3 Anemia:

- The first line response to symptomatic anemia is introduction of red cells, and consideration of growth factor support if participants require recurrent red cells transfusion.
- Symptomatic anemia should be treated with red blood cell transfusion and is recommended if the hemoglobin falls below 8 g/dL.
- The initiation of erythropoietic therapy for the management of chemotherapy-induced anemia follows the American Society of Hematology/ASCO clinical practice guidelines (http://www.asco.org).

- If grade 3/4 anemia is refractory to red cell transfusion and growth factor support within 3 weeks, then LY2606368 should be dose reduced to the next lower dose level.

Table 6: Treatment modifications of LY2606368 for non-hematologic toxicities [*]

Grade	***Occurrence***	***Immediate Action***	***Resumption of Therapy***
1	All	None	No interruption
2	All	Hold therapy until grade ≤1	No change in dose, but initiate appropriate medical therapy
3	1st **	Hold therapy until grade ≤1	No change in dose, but initiate appropriate medical therapy
	2nd**	Hold therapy until grade ≤1	Initiate appropriate medical therapy and then reduce LY2606368* to the next lower dose level upon re-initiation
4	1st	Discontinue therapy	

*For adverse events that are possibly, probably, or definitely attributable to LY2606368 alone (attributions 3-5)
Treatment will be discontinued if participants require the second dose reduction (see **3.3.1.1) see **3.3.3** below for exemptions of **

Drug will be reinitiated upon resolution of toxicity to grade 1 or better or as qualified below. Delay of treatment due to recovery may not persist longer than 3 weeks. Once a participant has a dose reduction for toxicity, the dose will not be increased back to the starting dose. Treatment will be discontinued if participants require the second dose reduction due to the occurrence of the same events.

3.3.2 QTc Prolongation

On study monitoring:

- ECGs will be performed before and at the end of infusion of each LY2606368 (within 30min +/- 20min), every two weeks x2, every 4 weeks x1, then every 3 months.
- For a single QTcF value of >500 ms, LY2606368 must be held. Electrolytes will be corrected and ECGs followed at least once per week, until QTcF falls below 480 ms.

- When drug is restarted after the QTcF prolongation has resolved within 3 weeks, ECGs (and electrolytes) should be followed every 2 weeks x2, every 4 weeks x1, then every 3 months.
- Serum potassium levels should be maintained ≥3.6 mEq/L, and serum magnesium and serum calcium should be kept within normal range to reduce the risk of QTcF prolongation. Some ovarian cancer participants who have had exposure to cis- and carboplatin may have persistent hypomagnesemia or hypokalemia despite replacement to the point of induction of diarrhea (if not within a normal range). Such participants will be closely monitored during the treatment, with IV potassium or additional dose of magnesium repletion.

3.3.3 Non-hematologic Toxicity

The participant will be removed from the study if a grade 4 non-hematologic toxicity occurs with the following exceptions:

- Asymptomatic hyperuricemia/hypouricemia/hypophosphatemia/hyponatremia with optimal repletion
- Asymptomatic hypomagnesemia with optimal repletion
- Asymptomatic hypocalcemia, or hypokalemia with optimal repletion; however, asymptomatic grade 4 hypokalemia not corrected to grade 1 or 0 within 48 hours despite optimal repletion will result in discontinuation of study drug.

  Subsequent grade 3 asymptomatic electrolyte toxicities that could be controlled with standard medications will not have dose reduction. However, asymptomatic grade 3 hypokalemia not corrected to grade 1 or 0 within 48 hours despite optimal repletion will result in dose reduction.

3.4 STUDY CALENDAR

Procedure	Screening/ Baseline	Cycle 1					Subsequent Cycles					
		Day 1	Day 8	Day 15	Day 22	Day 28	Day 1	Day 8	Day 15	Day 28	End of Tx Visit[11]	Post TX
LY2606368 Infusion[1]		X		X			X		X			
History and PE[2]	X	X		X			X				X	X
Vital signs[3]	X	X					X				X	X
Performance Score	X						X				**X**	
Labs (CBC w/diff, PT/INR/PTT, 14 metabolic panel (electrolytes, BUN, creatinine, glucose, AST, ALT, bilirubin, calcium, albumin, alkaline phosphatase), magnesium, urinalysis)[4]	X	X		X			X				X	X
Biopsies[5]	X			X								X
Correlative Research Studies (CTC, etc)[6]	X			X								X
CT Scan[7]	X						X					X
ECG[8]	X	X		X			X				X	X
Serum or Urine hCG[9]	X											
Adverse Events[2]	X	X	→	→	→	→	→	→			X	X
Concomitant Medications[2]	X	X	→	→	→	→	→	→			X	

		Cycle 1					Subsequent Cycles					
**Procedure**	**Screening/ Baseline**	**Day 1**	**Day 8**	**Day 15**	**Day 22**	**Day 28**	**Day 1**	**Day 8**	**Day 15**	**Day 28**	**End of Tx Visit[11]**	**Post TX**
NIH Advanced Directives Form[10]	X											

[1]Administration of LY2606368 may be delayed up to 7 days due to holidays, inclement weather, conflicts, or similar reasons. The timing of subsequent administrations is then adjusted to maintain a 2-week interval. For safety reasons, LY2606368 may not be administered with less than a 2-week interval.

[2]Performed every other week during Cycle 1 and every cycle (+/- one day due to holiday, inclement weather, or similar reasons) for safety measures in subsequent cycles. Phone follow up for symptom assessment every week during Cycle 1

[3]Performed before (anytime prior to Prexasertib infusion start) and after (within 30min +/- 30min) infusion of LY2606368

[4]Performed every two weeks (+/- one day due to holiday, inclement weather, or similar reasons). CBC will be repeated on Cycle 1 Day 8 (+/- one day due to holiday, inclement weather, or similar reasons) during Cycle 1 to check ANC nadir. May be omitted on Cycle 1 Day 1 if completed within 3 days of screening/baseline.

[5] For cohorts 1-3, second and at progression biopsies are optional, and strongly encouraged. For cohort 5, the second biopsy (= biopsy at progression) is mandatory for the responders (defined by CR/PR or SD ≥4 completed cycles of therapy. No biopsies are performed for cohort 6.

[6]Baseline, C1 D15 and at the time of progression for all participants.

[7]Groups 1-3, 5 and 6: Performed every 2 cycles (+/- 7 days), beginning with Cycle 3 Day 1, prior to drug administration. For participants who have been on study ≥ 4 years, CT scans will be performed every 3 cycles (+/-7 days). MRI may be used as clinically indicated for participants who cannot tolerate CT contrast.

[8]ECGs will be performed at screening/baseline, before (anytime prior to Prexasertib infusion start) and at the end of each infusion (within 30min +/-20min), C1D15, C2D1, C3D1, and then every 3 months (+/- 2 weeks) and post treatment. Baseline/screening EKG may be used as C1D1 pre-infusion EKG if done within one week.

[9]In women of childbearing potential

*[10]As indicated in section **12.3,** all subjects ≥ age 18 will be offered the opportunity to complete an NIH advanced directives form. This should be done preferably at baseline but can be done at any time during the study as long as the capacity to do so is retained. The completion of the form is strongly recommended, but is not required.*

[11]*End of treatment visit will occur approximately 30 days after the last dose of study drug. If the participant cannot return to the Clinical Center for this visit, a request will be made to collect required clinical labs (specify as needed) from a local physician or laboratory. If this is not possible, participants may be assessed by telephone for symptoms.*

3.5 COST AND COMPENSATION

3.5.1 Cost

NIH does not bill health insurance companies or participants for any research or related clinical care that participants receive at the NIH Clinical Center. If some tests and procedures performed outside the NIH Clinical Center, participants may have to pay for these costs if they are not covered by insurance company. Medicines that are not part of the study treatment will not be provided or paid for by the NIH Clinical Center.

3.5.2 Compensation

Participants will not be compensated on this study.

3.5.3 Reimbursement

The NCI will cover the costs of some expenses associated with protocol participation. Some of these costs may be paid directly by the NIH and some may be reimbursed to the participant/guardian as appropriate. The amount and form of these payments are determined by the NCI Travel and Lodging Reimbursement Policy.Criteria for Removal from Protocol Therapy and Off Study Criteria

Prior to documenting removal from study, effort must be made to have all subjects complete a safety visit approximately 30 days following the last dose of study therapy.

3.5.4 Criteria for removal from protocol therapy

In the absence of treatment delays due to adverse events, treatment may continue until one of the following criteria applies:

- Disease progression
- Intercurrent illness that prevents further administration of treatment
- Participant requests to be withdrawn from an active therapy.
- Participant withdraws consent. In this event, the reasons for withdrawal will be documented and the participant will be taken off study at that time.
- If participants are non-compliant with the protocol guidelines, they may be removed from the study at the discretion of the PI.
- Unacceptable adverse events as defined in section **3.3.3**
- Positive pregnancy test
- PI discretion: clinical disease progression

3.5.5 Off-Study Criteria

- Completed study follow-up period

- Participant requests to be withdrawn from study
- Death
- Start of another anti-cancer therapy
- Lost to follow up

3.5.6 Lost to follow up

A participant will be considered lost to follow-up if he or she fails to return for one scheduled treatment visit and is unable to be contacted by the study site staff.
The following actions must be taken if a participant fails to return to the clinic for a required study visit:

- The site will attempt to contact the participant by the methods described below to reschedule the missed visit within 7 days of the missed visit, counsel the participant on the importance of maintaining the assigned visit schedule, and ascertain if the participant wishes to and/or should continue in the study.
- Before a participant is deemed lost to follow-up, the investigator or designee will make every effort to regain contact with the participant within 7 days (a minimum of 3 contact attempts using the participant's preferred method of contact via phone, email, or a combination of both; and if necessary, a letter sent through a trackable delivery service to the participant's last known mailing address. These contact attempts should be documented in the participant's medical record or study file.
- Should the participant continue to be unreachable, he or she will be considered to have withdrawn from the study with a primary reason of lost to follow-up.

4 CONCOMITANT MEDICATIONS/MEASURES

- Other cancer chemotherapy, radiation therapy, immunotherapy, or investigational agents may not be administered to participants while they are receiving LY2606368 on this study.
- Concomitant use of medications generally known to cause prolonged QTc or Torsades de Pointes (**Appendix B**) are not allowed within 12 hours before and after the infusion of LY2606368.
- Preliminary results suggest that drug–drug interactions may be possible although clinical drug-drug interaction is unlikely, when LY2606368 is co-administered with medications that are classified as sensitive cytochrome P450 (CYP)1A2 and CYP2D6 substrates. This should be considered by the investigator when LY2606368 is administered to participants requiring treatment with sensitive CYP1A2 and CYP2D6 substrates, such as duloxetine, alosetron, and desipramine, or with CYP1A2 and CYP2D6 substrates that have a narrow therapeutic range, such as theophylline, tizanidine, and thioridazine.
- Because LY2606368 interacts with multiple 5-hydroxytryptamine (5-HT) receptors, participants should be monitored closely by a home physician or investigators at CCR for any signs and symptoms of serotonin syndrome.

Combined use of selective serotonin reuptake inhibitor (SSRI)s or change SSRIs without wash-out could potentially lead to serotonin syndrome. Serotonin syndrome is a potentially life threatening drug reaction due to excess of serotonergic activity at central nervous system (CNS) and peripheral serotonin receptor, and symptoms and signs may include agitation or restlessness, severe diarrhea, tachycardia, and hypertension. If observed, appropriate treatment (e.g., cyproheptadine or octreotide) may be used as a treatment.

- Alternative antiemetic medications available at the Clinical Center that will not prolong QTc interval, not affect 5-HT receptors, and not affect CYP1A2 and CYP2D6 substrates are listed below. These medications may be used for symptomatic relief of nausea: dexamethasone (glucocorticoid; CYP3A subfamily inducer), methylprednisolone (glucocorticoid; CYP3A subfamily substrate and inducer), lorazepam (benzodiazepine; undergoes phase II metabolism by glucuronidation; 85 to >90% protein bound), granisetron (serotonin 5 HT3 receptor antagonist with least potential for perturbing cardiac conduction among the pharmacological class; CYP3A4 substrate)
- Supportive care measures and symptomatic treatment for any treatment-associated toxicity may be instituted once the first signs of toxicity occur and should be recorded in the participant's chart.

5 CORRELATIVE STUDIES AND BIOSPECIMEN COLLECTION

5.1 CORRELATIVE STUDIES FOR RESEARCH/PHARMACODYNAMIC STUDIES

5.1.1 Rationale for Correlative Studies

The goal of the correlative studies is to identify potential predictive and proof-of-concept biomarkers of Chk1/2 inhibitor in BRCA mutation-associated and BRCA-like breast and ovarian cancers. In the context of this study, participants of Cohorts 1-3 will undergo *one mandatory and two optional (highly encouraged)* biopsies to be used to characterize the effects that LY2606368 upon the biology of both the tumor and the stroma. The timing of biopsies (baseline, C1d15 [6-24hr post-2nd dose], and at progression) has been defined to get tumor samples to investigate the biochemical changes by replication stress and in response to treatment.

In addition, we will study potential resistance mechanisms of LY2606368 in responders with recurrent platinum-resistant HGSOC. For cohort 5, all participants will undergo pretreatment biopsy. *The mandatory second biopsy (=at progression) will be done only for the responders (defined by CR/PR or SD ≥ 4 months).* The second biopsy may be performed if the sum of target lesions increases 15 +/- 4% from the best response before confirmed PD by RECIST v1.1 criteria, due to holidays, inclement weather, conflicts, or similar reasons.

These tumor biopsies will be obtained percutaneously or per vagina through Interventional Radiology as long as considered minimal surgical risk. Participants must agree to the mandatory biopsy prior to signing consent and enrolling on study. Participants may withdraw their consent

for optional biopsies after enrolling on the study and treatment will not be prejudiced. Molecular profiling, genomic sequencing and/or immune subsets panel will be used to evaluate target and related pathways. Further detailed molecular analysis of tumor and stroma may lead to an understanding of the mechanisms mediating clinical response, tumor progression, and toxicities.

Blood samples for circulating tumor cells (CTCs) and immune panel analyses will be collected at the following time points: at baseline, C1d15 (6-24hr post-2nd dose), and at progression for all study participants (cohorts 1-3, 5 and 6). Research samples (supernatant and pellet) to test for HR deficiency will be collected from participants who undergo either therapeutic or diagnostic thoracentesis or paracentesis while on study. Samples may be saved, and anonymized for LY2606368 concentration measurement in the body fluid.

All participants on the study are requested to submit a block of tissue or 20 paraffin-capped unstained slides from a recent resection or barring that, from the original surgery. All efforts will be made to obtain these samples to achieve the secondary objectives.

5.1.2 Tumor biopsies

5.1.2.1 Rationale

Cohorts 1-3: The on-study biopsy and the optional second biopsy are necessary for the execution of the critical translational endpoints evaluating proof of concept and demonstration of mechanism and activity.

Cohort 5: Paired baseline and at progression biopsies are necessary to investigate potential resistance mechanisms in clinical responders to LY2606368

5.1.2.2 Timing*

Cohorts 1-3: Biopsies will be performed at the following times:

- Mandatory – after consent, prior to treatment on cycle 1 day 1
- Optional - cycle 1 day 15 (6-24hr post-2nd dose)
- Optional - at the time of progression

Cohort 5: Biopsies will be performed at the following times:

- Mandatory – after consent, prior to treatment on cycle 1 day 1
- Mandatory second biopsy for the responders (CR, PR and SD ≥ 4 completed cycles of therapy) at progression.

**Biopsies may be not performed on the specific dates and times due to the following reasons, including but not limited to, delayed recovery of hematologic toxicities, delayed clinic schedule, or national holidays.*

Attempts will be made to obtain four cores if safe and feasible, which will be frozen for research studies. Two 3- millimeter punch biopsies of skin will be acceptable in lieu of 18 gauge core biopsies for participants with skin involvement. Inability to get tissue with a reasonable attempt will not preclude treatment and the participant will remain eligible for all other translational components. The use of imaging to facilitate biopsies will be decided upon by members of the interventional radiology team. Should CT scans be needed for biopsy, a limit of 10 scans for each procedure will be observed to minimize radiation exposure to the participant.

The biopsies (up to three cores) are to be immediately embedded, frozen, and stored in the Lee lab at -80^{0}C on site according to our laboratory SOP (**Appendix C**). The schedule for the biopsies will be made with Special Procedures (Dr. Brad Wood).

- For groups 1-3 and 5, members of the Lee lab will be on call to receive and embed biopsies: jayakumar.nair@nih.gov, Phone (301) 451-8403, beeper 102-11155.

Each participant sample set will be assigned a unique participant identifier. The protocol scientific investigator(s) handling the samples will be blinded as to the participant identification, participant data and outcome.

Frozen biopsies will be sectioned, stained, and subjected to tissue lysate array analysis as below (section **5.1.3.1**).

If an attempt at biopsy is unsuccessful, the participant will still be eligible for treatment and the subsequent biopsies will be foregone.

Biopsy material will be prioritized for proteomics. The remaining tissue will be released for additional testing prioritized to genomic sequencing, cell cycle check point pathways analyses, and immunohistochemistry, in that order.

5.1.3 Laboratory Correlative Studies

5.1.3.1 Tissue proteomic Analysis

Protein microarrays will be employed to quantitate activated and inactive signaling components of the DNA damage repair and cell cycle check point pathway proteins before and at two weeks of therapy. Analysis of specific proteins and their activation status allows interrogation of the Chk1/2 pathway and collateral pathways at multiple nodes. Target proteins can include but not limited to: ATM, pS1981 ATM, Chk1, pS345 Chk1, Chk2, pT68 Chk2, CDK1, FOXO3a, pS318/S321 FOXO3a, 53BP1. The expression and activation state of these proteins will be correlated with tumor response or resistance, and toxicity.

These proteomics endpoints will be studied using an Acrivon Predictive Precision Proteomics (AP3) platform in collaboration with Dr. Shipitsin at Acrivon. The AP3 method is based on both unbiased global phosphoproteomics drug profiling by mass spectrometry as well as biased tumor cell and human tumor intact tissue quantitative protein analyses. Results will be returned to the study team.

Coded participant samples will be batched to:

Dr. Michail (Misha) Shipitsin
Acrivon Therapeutics
Lab Central, 700 North Main Street,
Cambridge, MA 02139
Phone: 781-526-3759
Email: mshipitsin@acrivon.com

5.1.3.2 Saliva Samples

Group 4 mCRPC cohort only: a one-time saliva sample will be collected anytime during the study as a source of normal tissue for genomics comparison. Each saliva sample will be collected in one Oragene Saliva Kit at room temperature, and Dr. Figg's lab will be paged for pick-up at 102-11964. Dr. Figg's lab will barcode the sample, remove participant identifier, and send the sample to Dr. Meltzer's lab, Bldg. 37, Rm 6138 via courier. Participants will be instructed not to eat, drink, smoke or chew gum for 30 minutes before giving their saliva sample. Sequencing of DNA will be performed by the Genetics Branch (Drs. Khan/Meltzer).

5.1.3.3 RNAseq or next generation sequencing and genomic analysis

RNAseq or exome sequencing will be done to investigate potential predictive biomarkers in responders and non-responders to LY2606368 in the Sequencing facility, Frederick National Laboratory for Cancer Research. Tumor biopsies will be obtained at baseline (mandatory), 6-24hr after the second dose (optional), and time of disease progression (optional for Cohorts 1-3 but mandatory for cohort 5 responders).

The results of molecular studies conducted using specimens are for research purposes only and will not be disclosed to individual subjects. The exception to this is potential incidental findings that are deemed medically significant and actionable. The policy for this disclosure is outlined below and in the consent form for this study.

- Subjects will be contacted if a clinically actionable gene variant is discovered. Clinically actionable findings for the purpose of this study are defined as disorders appearing in the American College of Medical Genetics and Genomics recommendations for the return of incidental findings that is current at the time of primary analysis. (A list of current guidelines is maintained on the CCR intranet: https://ccrod.cancer.gov/confluence/display/CCRCRO/Incidental+Findings+Lists). Subjects will be contacted at this time with a request to provide a blood sample to be sent to a CLIA certified laboratory which has an FDA approved test for that mutation. If the research findings are verified in the CLIA certified lab, the subject will be referred to the CCR Genetics Branch for the disclosure of the results.
- This is the only time during the course of the study that incidental findings will be returned. No interrogations regarding clinically actionable findings will be made after the primary analysis.

- Contact information must be maintained on subjects to have their results returned. If a subject's participation in the study ends prior to the primary analysis, they should be enrolled on study 04-C-0165, a standard of care protocol, in order to allow for post study contact for the dissemination of any incidental findings and the maintenance of contact information.
- Subject's genetic data may be deposited in a database such as dbGaP. Although there is controlled access to such a database, such a submission carries theoretical risks of revealing the identity of the subject. This is discussed in the consent.

To evaluate potential predictive biomarkers, Dr. Elloumi at NCI will review, process and integrate the raw data (fastq files) from RNAseq and exome sequencing using the cellminer/patientMiner website (via VPN): https://discovery.nci.nih.gov/rsconnect/patientMiner_hg38/. All data and clinical information will be de-identified and access is controlled by the PI and collaborator.

Fathi Elloumi, PhD
Staff Scientist
Developmental Therapeutics Branch, Center for Cancer Research, NCI
Phone: 240-760-6601
fathi.elloumi@nih.gov

5.1.3.4 Cell Cycle Checkpoint Assays

Tests will be performed on participants' tumor tissues by proteomic microarray, immunohistochemistry (IHC) or immunoblot. These will include but are not limited to, γH2AX, Chk1, p296serChk1, p317serChk1, p345serChk1, Chk2, Ki67, pY15Cdk1, CCNE1 and caspase-3. The IHC will be studied in collaboration with Dr. Cimino-Mathews at Johns Hopkins University Hospital.
Coded participant samples will be batched to:

Ashley Cimino-Mathews, MD
Assistant Professor, Department of Pathology
The Johns Hopkins Hospital
401 N. Broadway St, Rm 2242
Baltimore, MD 21287
Phone: 410-955-3580
Email: acimino@jhmi.edu

Studies to be performed on paired pre- and/or post-treatment biopsies and on archival tissue:

- H&E staining
- p345serChk1
- p296serChk1
- p15TyrCDC2
- p123SerCDC25A and p216SerCDC25C (response biomarkers for cell cycle regulators)

Should there be sufficient tumor tissue remaining, exploratory correlative endpoints including but not limited to the following will be performed:

- p317serChk1
- Chk2
- pT68 Chk2
- γH2AX
- Ki-67
- IKKε[37]

5.1.3.5 Immune subsets and cell cycle pathways

Baseline and post-therapy tumor core biopsies will be analyzed for the immunoreactive subtype of ovarian cancer and/or DNA repair and cell cycle pathways, by gene expression analysis. Available core sample will be frozen and stored for analysis of gene expression via droplet digital PCR, real time PCR, NanoString or next-generation sequencing technology.

5.1.3.6 Regulatory B cells analysis (Bregs)

Data suggest Bregs promote distant metastasis by empowering other regulatory immune cells, such as regulatory T cells and myeloid suppressive cells. Further studies will be done using clinical samples for its relation to bone marrow progenitor cells and clinical characteristics in an exploratory fashion.

Archival or available biopsy or blood samples of triple negative breast cancer participants will be evaluated for bone marrow progenitor markers using Flow cytometry, real time PCR, NanoString, microarray, or RNA sequencing assays in collaboration with Arya Biragyn, PhD.

Coded, linked participant samples will be batched to:
Arya Biragyn, PhD
Chief, Senior Investigator, Immunoregulation Section, LMBI,
251 Bayview Blvd, suite 100, Rm.06C228
National Institute on Aging
Baltimore, MD 21224.
Ph. (410)558-8680
Email: biragyna@grc.nia.nih.gov

5.1.4 Blood Samples

Studies to be performed in blood samples:

5.1.4.1 Circulating Tumor Cells

- Blood will be collected to correlate changes in circulating tumor cells[38] with clinical response. CTCs enumeration will be investigated using ferrofluidic enrichment and multi-parameter flow cytometric detection. CTCs are identified by positive expression of epithelial markers and a viability marker and negative expression of hematopoietic markers.
- Collection of Specimen(s): Two 8-mL EDTA tubes (lavender top tubes) will be collected from each participant at the following time points: baseline, and C1d15 (4-24hr after second dose), and at time of progression. Plasma from these samples will be stored at Trepel lab after CTCs are harvested.
- Immediately after collection, invert the blood tubes 3-4 times.
- Notification and Handling of Samples: as soon as possible after the participant is scheduled please send email notification to the Trepel lab: Jane Trepel at trepel@helix.nih.gov; Min-Jung Lee at leemin@mail.nih.gov that the sample is scheduled. As soon as the sample is drawn, please call the Trepel lab at 240-760-6330 to communicate that the sample is ready. Keep the sample on the unit at room temperature. The sample will be picked up by the lab and processed for CTC enumeration.

5.1.4.2 Immune Subsets

- Peripheral blood mononuclear cells (PBMC) will be assessed using multiparameter flow cytometry for immune subsets including but not necessarily limited to Tregs, MDSC, effector and exhausted CD8+ T-cells. Assessment will include functional markers, i.e. PD-1, TIM3, CTLA-4 and/or CD40.
- Collection of Specimen(s): Two 8-ml BD Vacutainer Cell Preparation Tubes (CPT) will be collected from each participant at the following time points: baseline, and C1d15 (4-24hr after second dose), and at time of progression.
- Immediately after collection, mix the blood sample by gentle inversion several times. The date and exact time of each blood draw should be recorded on the tube.
- Notification and Handling of Samples: as soon as possible after the participant is scheduled please send email notification to the Trepel lab at trepel@helix.nih.gov; Min-Jung Lee at leemin@mail.nih.gov) and call the Trepel lab at 240-760-6330 to arrange for immediate pick-up when the sample is drawn.

5.1.4.3 γH2AX/Mre11

- PBMC samples will be collected and archived for flow cytometric determination of DNA damage and repair using flow cytometric γH2AX and MRE11 dual-labeling assay.
- Cohorts 1-3: Samples will be collected in Two-8ml BD Vacutainer CPT (blue/black tubes) at baseline only.
- Cohort 5 and 6: Samples will be collected in Two-8ml BD Vacutainer CPT at baseline, C1d15 (4-24hr after second dose), and at time of progression..
- The same unique participant identifier linked to the tumor biopsies will be used to shield the archived blood samples.

- Notification and Handling of Samples: as soon as possible after the participant is scheduled please send email notification to the Trepel lab at trepel@helix.nih.gov; Min-Jung Lee at leemin@mail.nih.gov) and call the Trepel lab at 240-760-6330 to arrange for immediate pick-up when the sample is drawn.

5.1.5 Other body fluids samples

Fluid specimens may be collected from participants with abnormal fluid collections who undergo either therapeutic or diagnostic thoracentesis or paracentesis while on study. Up to one liter of fluid may be collected, depending on the source and volume of the abnormal fluid collection. Standard techniques will be used for fluid collection that may include CT and/or ultrasound-guided aspiration.

5.1.6 Radiomics

5.1.6.1 Rationale

Tissue based biomarkers remain the gold standard for pharmacodynamics studies but need to be carefully calibrated to the most informative tumor region, which is currently very difficult to achieve in a multi-site heterogeneous disease such as recurrent ovarian cancer.

Professor Evis Sala's lab at the University of Cambridge in U.K. has been a leading expert in the quantitative assessment of image features, known as radiomics, using their artificial intelligence (AI) tools for (a) automatic whole volume tumor segmentation and (b) deriving predictive and prognostic models of response and outcome. Radiomics biomarkers therefore could serve as "virtual biopsies" and may provide biomarkers reporting changes in both tumor and microenvironment during therapy and have the potential to personalize treatment by objective assessment of these effects. This project is supported by the Biomarkers Consortium by Foundation of National Institutes of Health.

Separately, Dr. Summer's group at NIH will use AI to automatically detect and quantify ovarian cancer on CT scans to evaluate potential predictive biomarkers. (**5.1.6.2**, **5.1.6.3**).

All imaging, reports, and clinical information will be de-identified.

5.1.6.2 Collaborators

Evis Sala, MD, PhD, FRCR
Professor of Oncological Imaging
University of Cambridge
Department of Radiology
Box 218, Cambridge Biomedical Campus
Cambridge CB2 0QQ
United Kingdom
Phone: +44 1223 746440

Email: es220@cam.ac.uk

Professor James D. Brenton PhD FRCP
Senior Group Leader and Honorary Consultant in Medical Oncology
Functional Genomics of Ovarian Cancer Laboratory
Cancer Research UK Cambridge Institute
University of Cambridge
Li Ka Shing Centre
01223 769761 Assistant
mailto:james.brenton@cruk.cam.ac.uk
http://www.cruk.cam.ac.uk/research-groups/brenton-group

Ronald M. Summers, MD, PhD, FSAR, FAIMBE
Senior Investigator and Staff Radiologist
Chief, Imaging Biomarkers and Computer-Aided Diagnosis Laboratory
Radiology and Imaging Sciences
National Institutes of Health Clinical Center
Building 10 Room 1C224D MSC 1182
Bethesda, MD 20892-1182
Phone (office): (301) 402-5486
Fax: (301) 451-5721
E-mail: rms@nih.gov
https://www.cc.nih.gov/meet-our-doctors/rsummers.html

5.1.6.3 Procedure

- An independent review of all scans of ovarian cancer participants will be conducted by Professor Sala's lab in order to develop and validate precise and robust radiogenomics biomarkers to optimize response assessment.
- Separately, Dr. Summers' group will review scans with an aim of training AI on the ovarian cancer and to evaluate potential predictive biomarkers.
- Copies of all imaging assessments including unscheduled visit scans will be collected on an ongoing basis and sent to the collaborators listed in **5.1.6.2**.
- Prior radiation therapy reports for participants (at baseline) and information on any lesions that were biopsied to provide a tumor sample for study entry will be provided to allow the selection of appropriate target lesions.
- Results of this scan review will not be communicated to Investigators to make a clinical decision.

5.2 Sample Storage, Tracking and Disposition

Samples will be ordered in CRIS and tracked through a Clinical Trial Data Management system. Should a CRIS screen not be available, the CRIS downtime procedures will be followed. Samples will not be sent outside NIH without appropriate approvals and/or agreements, if required.

Each participant sample set will be assigned a unique participant identifier in the recipient lab with bar coding in the Clinical Pharmacology Program (CPP) and Lee laboratories and the scientific investigator handling and performing analyses on the samples will be blinded as to the sample source, participant data, and outcome. There will be no date identifiers on the samples. Samples will be grouped for scientific analyses. If a participant needs to have a malignant effusion tapped for diagnostic or therapeutic purposes, a sample will be collected for research. At the end of the protocol, samples will be stored for potential further analysis as new information becomes available (only for those subjects who consented to future optional studies). Any new use of identified or coded samples, specimens, or data will undergo prospective and continuing IRB review and approval.

Samples, and associated data, will be stored in the locations listed above unless the participant withdraws consent. If researchers have samples remaining once they have completed all studies associated with the protocol, they must be returned to Dr. Lee's laboratory or to the CPP repository. Samples can only be saved at completion of study for future use if subjects consent. Access to these samples will only be granted following IRB approval of an additional protocol, granting the rights to use the material.

If, at any time, a participant withdraws from the study and does not wish for their existing samples to be utilized, the individual must provide a written request. Following receipt of this request, the samples will be destroyed (or returned to the participant, if so requested). The PI will record any loss or unanticipated destruction of samples as a deviation. Reporting will be per the requirements of section **7.2**.

5.2.1 Clinical Pharmacology Program

5.2.1.1 Procedures for sample data collection for the Clinical Pharmacology Program:

- All samples sent to the Clinical Pharmacology Program (CPP) will be barcoded, with data entered and stored in Labmatrix. This is a secure program, with access to Labmatrix limited to defined CPP personnel, who are issued individual user accounts. Installation of Labmatrix is limited to computers specified by Dr. Figg. These computers all have a password restricted login screen.
- Labmatrix creates a unique barcode ID for every sample and sample box, which cannot be traced back to participants without Labmatrix access. The data recorded for each sample includes the participant ID, name, trial name/protocol number, time

drawn, cycle time point, dose, material type, as well as box and freezer location. Participant demographics associated with the clinical center participant number are provided in the system. For each sample, there are notes associated with the processing method (delay in sample processing, storage conditions on the ward, etc.).

5.2.1.2 Procedures for sample storage at the Clinical Pharmacology Program:

- Barcoded samples are stored in barcoded boxes in a locked freezer at either -20°C or -80°C according to stability requirements. These freezers are located onsite in the CPP and offsite at NCI Frederick repository facilities in Frederick, MD. Visitors to the laboratory are required to be accompanied by laboratory staff at all times.
- Access to stored clinical samples is restricted. Samples will be stored until requested by a researcher named on the protocol. All requests are monitored and tracked in Labmatrix. All researchers are required to sign a form stating that the samples are only to be used for research purposes associated with this trial (as per the IRB approved protocol) and that any unused samples must be returned to the CPP. It is the responsibility of the NCI Principal Investigator to ensure that the samples requested are being used in a manner consistent with IRB approval.
- Following completion of this study, samples will remain in storage as detailed above. Access to these samples will only be granted following IRB approval of an additional protocol, granting the rights to use the material.
- Sample barcodes are linked to participant demographics and limited clinical information. This information will only be provided to investigators listed on this protocol, via registered use of Labmatrix. It is critical that the sample remains linked to participant information such as race, age, dates of diagnosis and death, and histological information about the tumor, in order to correlate genotype with these variables.

5.3 SAMPLES FOR CORRELATIVE STUDIES ANALYSIS

5.3.1 Management of Correlative Studies Results

The analyses performed in various NCI laboratories under this protocol are for research purposes only. Independently of the research testing, we will provide guidance and referral (within the NIH or outside) to participants requesting additional counseling and testing.

Any comparisons involving biochemical changes from baseline to after treatment will be considered exploratory, and reported as such, without formal adjustment for multiple comparisons. Furthermore, since this study is mainly pilot in intent, the findings will be reported in that context. Any such evaluations will be done with exploratory intent, without adjustment for multiple comparisons, and with the results reported in that context.

6 DATA COLLECTION AND EVALUATION

6.1 Data Collection

The PI will be responsible for overseeing entry of data into an in-house password protected electronic system (C3D) and ensuring data accuracy, consistency and timeliness. The principal investigator, associate investigators/research nurses and/or a contracted data manager will assist with the data management efforts. Primary and final analyzed data will have identifiers so that research data can be attributed to an individual human subject participant.

All adverse events (AEs), including clinically significant abnormal findings on laboratory evaluations, regardless of severity, will be followed until return to baseline or stabilization of event.

AEs will be documented from the first study intervention, Study Day 1, through 30 days after the subject received the last dose of the study agent. Beyond 30 days after the last intervention, only adverse events which are serious and related to the study intervention need to be recorded.

An abnormal laboratory value will be considered an AE if the laboratory abnormality is characterized by any of the following:

- Results in discontinuation from the study
- Is associated with clinical signs or symptoms
- Requires treatment or any other therapeutic intervention
- Is associated with death or another serious adverse event, including hospitalization.
- Is judged by the Investigator to be of significant clinical impact

If any abnormal laboratory result is considered clinically significant, the investigator will provide details about the action taken with respect to the test drug and about the participant's outcome.

An enrollment log will be maintained in the regulatory binder/file; and source documents, including a family history questionnaire (**Appendix D**) will be stored in the research record for each participant.

Quality assurance requires maintaining complete records on each participant treated on the protocol, including primary documentation (e.g., laboratory reports, X-ray reports, scan reports, pathology reports, physician notes, etc.) that confirms the following:

- The participant met each eligibility criterion.
- Signed informed consent was obtained prior to treatment. (An on-study confirmation of eligibility form will be filled out before entering the study.)

- Documentation of specific dates and times of all treatments, doses administered, and the reason for any dose modification.
- Toxicity was assessed according to protocol (see section 6.3)
- Response was assessed according to protocol (X-ray, scan, lab reports, dated notes on measurements, and clinical assessment, as appropriate).
- NCI Drug Accountability Records were kept for each participant.

Clinical data will be entered in a secure electronic database, NCI C3D database, and hard copies will be stored in locked, secured areas. Completed eligibility checklists, participant information/registration forms, and blood sample flow sheets will also be stored. Copies of all records of serious adverse events will be kept in the research record.

Complete records must be maintained on each participant, including the hospital chart with any supplementary information obtained from outside laboratories, radiology reports, or physician's records. These records will serve as the primary source material that forms the basis for the research record. All relevant data will also be entered in a computer database from which formal analyses will be done.

The primary source documentation will include: on-study information, including participant eligibility data and participant history; flow sheets, records of adverse events, specialty forms for pathology, radiation, or surgery; and off-study summary sheets, including a final assessment by the treating physician.

End of study procedures: Data will be stored according to HHS, FDA regulations and NIH Intramural Records Retention Schedule as applicable.

Loss or destruction of data: Should we become aware that a major breach in our plan to protect subject confidentiality and trial data has occurred, this will be reported expeditiously per requirements in section **7.2.1**.

6.2 DATA SHARING PLAN

6.2.1 Human Data Sharing Plan

What data will be shared?

I will share human data generated in this research for future research as follows:
_X_Coded, linked data in BTRIS (automatic for activities in the Clinical Center)

How and where will the data be shared?

Data will be shared through:
_X_BTRIS (automatic for activities in the Clinical Center)

6.2.2 Genomic Data Sharing Plan

Unlinked genomic data will be deposited in public genomic databases such as dbGaP in compliance with the NIH Genomic Data Sharing Policy.

6.3 RESPONSE CRITERIA

For the purposes of this study, participants should be re-evaluated for response every 2 cycles (+/- 7 days) for Cohorts 1-3, 5, and 6. In addition to a baseline scan, confirmatory scans should also be obtained 4 weeks (+/- 7 days) following initial documentation of objective response. The timing of subsequent re-evaluation for response is then adjusted to continue every 2 cycles (+/- 7 days). Participants who have been on study four years or longer should be re-evaluated for response every 3 cycles (+/-7 days).

Response and progression will be evaluated in this study using the new international criteria proposed by the revised Response Evaluation Criteria in Solid Tumors (RECIST) guideline (version 1.1)[39]. Changes in the largest diameter (unidimensional measurement) of the tumor lesions and the shortest diameter in the case of malignant lymph nodes are used in the RECIST criteria. For group 4, participants will be assessed primarily by CT scan of the chest, abdomen and pelvis and by technetium 99 bone scan at baseline. Participants will undergo CT imaging, technetium 99 bone scintigrapy (and research imaging if applicable) every 12 weeks (+/- 4 days). Response and progression will be evaluated in this study using the new international criteria proposed by the revised Response Evaluation Criteria in Solid Tumors (RECIST) guideline (version 1.1)[39] and Prostate Cancer Clinical Trials Working Group criteria (PCWG2)[36]. [Effective with Amendment I (version date 4/24/2017), mCRPC cohort 4 was closed to accrual.] Changes in the largest diameter (unidimensional measurement) of the tumor lesions and the shortest diameter in the case of malignant lymph nodes are used in the RECIST criteria.

6.3.1 Definitions

Evaluable for toxicity: All participants will be evaluable for toxicity from the time of their first treatment with LY2606368.

Evaluable for objective response: Only those participants who have measurable disease present at baseline, have received at least one cycle of therapy, and have had their disease re-evaluated will be considered evaluable for response. These participants will have their response classified according to the definitions stated below. (Note: Participants who exhibit objective disease progression prior to the end of cycle 1 will also be considered evaluable.)

Evaluable Non-Target Disease Response: Participants who have lesions present at baseline that are evaluable but do not meet the definitions of measurable disease, have received at least one cycle of therapy, and have had their disease re-evaluated will be considered evaluable for non-target disease. The response assessment is based on the presence, absence, or unequivocal progression of the lesions.

6.3.2 Disease Parameters

Measurable disease: Measurable lesions are defined as those that can be accurately measured in at least one dimension (longest diameter to be recorded) as:

- By chest x-ray: ≥20 mm;
- By CT scan:
    - Scan slice thickness 5 mm or under: as ≥10 mm
    - Scan slice thickness >5mm: double the slice thickness
- With calipers on clinical exam: ≥10 mm.

All tumor measurements must be recorded in millimeters (or decimal fractions of centimeters).

Malignant lymph nodes. To be considered pathologically enlarged and measurable, a lymph node must be ≥15 mm in short axis when assessed by CT scan (CT scan slice thickness recommended to be no greater than 5 mm). At baseline and in follow-up, only the short axis will be measured and followed.

Non-measurable disease. All other lesions (or sites of disease), including small lesions (longest diameter <10 mm or pathological lymph nodes with ≥10 to <15 mm short axis), are considered non-measurable disease. Bone lesions, leptomeningeal disease, ascites, pleural/pericardial effusions, lymphangitis cutis/pulmonitis, inflammatory breast disease, and abdominal masses (not followed by CT or MRI), are considered as non-measurable.

Note: Cystic lesions that meet the criteria for radiographically defined simple cysts should not be considered as malignant lesions (neither measurable nor non-measurable) since they are, by definition, simple cysts.

'Cystic lesions' thought to represent cystic metastases can be considered as measurable lesions, if they meet the definition of measurability described above. However, if non-cystic lesions are present in the same participant, these are preferred for selection as target lesions.

Target lesions. All measurable lesions up to a maximum of 2 lesions per organ and 5 lesions in total, representative of all involved organs, should be identified as **target lesions** and recorded and measured at baseline. Target lesions should be selected on the basis of their size (lesions with the longest diameter), be representative of all involved organs, but in addition should be those that lend themselves to reproducible repeated measurements. It may be the case that, on occasion, the largest lesion does not lend itself to reproducible measurement in which circumstance the next largest lesion which can be measured reproducibly should be selected. A sum of the diameters (longest for non-nodal lesions, short axis for nodal lesions) for all target lesions will be calculated and reported as the baseline sum diameters. If lymph nodes are to be included in the sum, then only the short axis is added into the sum. The baseline sum diameters will be used as reference to further characterize any objective tumor regression in the measurable dimension of the disease.

Non-target lesions. All other lesions (or sites of disease) including any measurable lesions over and above the 5 target lesions should be identified as **non-target lesions** and should be recorded at baseline. Measurements of these lesions are not required, but the presence, absence, or in rare cases unequivocal progression of each should be noted throughout follow-up.

6.3.3 Methods for Evaluation of Measurable Disease

All measurements should be taken and recorded in metric notation using a ruler or calipers. All baseline evaluations should be performed as closely as possible to the beginning of treatment and never more than 4 weeks before the beginning of the treatment.
The same method of assessment and the same technique should be used to characterize each identified and reported lesion at baseline and during follow-up. Imaging-based evaluation is preferred to evaluation by clinical examination unless the lesion(s) being followed cannot be imaged but are assessable by clinical exam.

Clinical lesions: Clinical lesions will only be considered measurable when they are superficial (e.g., skin nodules and palpable lymph nodes) and ≥10 mm diameter as assessed using calipers (e.g., skin nodules). In the case of skin lesions, documentation by color photography, including a ruler to estimate the size of the lesion, is recommended.

Chest x-ray: Lesions on chest x-ray are acceptable as measurable lesions when they are clearly defined and surrounded by aerated lung. However, CT is preferable.

Conventional CT and MRI: This guideline has defined measurability of lesions on CT scan based on the assumption that CT slice thickness is 5 mm or less. If CT scans have slice thickness greater than 5 mm, the minimum size for a measurable lesion should be twice the slice thickness. MRI is also acceptable in certain situations (e.g. for body scans).
Use of MRI remains a complex issue. MRI has excellent contrast, spatial, and temporal resolution; however, there are many image acquisition variables involved in MRI, which greatly impact image quality, lesion conspicuity, and measurement. Furthermore, the availability of MRI is variable globally. As with CT, if an MRI is performed, the technical specifications of the scanning sequences used should be optimized for the evaluation of the type and site of disease. Furthermore, as with CT, the modality used at follow-up should be the same as was used at baseline and the lesions should be measured/assessed on the same pulse sequence. It is beyond the scope of the RECIST guidelines to prescribe specific MRI pulse sequence parameters for all scanners, body parts, and diseases. Ideally, the same type of scanner should be used and the image acquisition protocol should be followed as closely as possible to prior scans. Body scans should be performed with breath-hold scanning techniques, if possible.

Tumor markers: Tumor markers alone cannot be used to assess response. If markers are initially above the upper normal limit, they must normalize for a participant to be considered in complete clinical response. Specific guidelines for both CA-125 response (in recurrent ovarian cancer) and PSA response (in recurrent prostate cancer) have been published[40-42]. In addition, the Gynecologic Cancer Intergroup has developed CA-125 progression criteria which are to be integrated with objective tumor assessment for use in first-line trials in ovarian cancer[43].

6.3.4 Response Criteria

6.3.4.1 Evaluation of Target Lesions

Complete Response[32]: Disappearance of all target lesions. Any pathological lymph nodes (whether target or non-target) must have reduction in short axis to <10 mm.

Partial Response (PR): At least a 30% decrease in the sum of the diameters of target lesions, taking as reference the baseline sum of diameters.

Progressive Disease (PD): At least a 20% increase in the sum of the diameters of target lesions, taking as reference the smallest sum on study (this includes the baseline sum if that is the smallest on study). In addition to the relative increase of 20%, the sum must also demonstrate an absolute increase of at least 5 mm. (Note: the appearance of one or more new lesions is also considered progressions).

Stable Disease (SD): Neither sufficient shrinkage to qualify for PR nor sufficient increase to qualify for PD, taking as reference the smallest sum of diameters while on study.

6.3.4.2 Evaluation of Non-Target Lesions

Complete Response (CR): Disappearance of all non-target lesions and normalization of tumor marker level. All lymph nodes must be non-pathological in size (<10 mm short axis).
Note: If tumor markers are initially above the upper normal limit, they must normalize for a participant to be considered in complete clinical response.

Non-CR/Non-PD: Persistence of one or more non-target lesion(s) and/or maintenance of tumor marker level above the normal limits.

Progressive Disease (PD): Appearance of one or more new lesions and/or *unequivocal progression* of existing non-target lesions. *Unequivocal progression* should not normally trump target lesion status. It must be representative of overall disease status change, not a single lesion increase.

Although a clear progression of "non-target" lesions only is exceptional, the opinion of the treating physician should prevail in such circumstances, and the progression status should be confirmed at a later time by the review panel (or Principal Investigator).

6.3.4.3 Evaluation of Best Overall Response

The best overall response is the best response recorded from the start of the treatment until disease progression/recurrence (taking as reference for progressive disease the smallest measurements recorded since the treatment started). The participant's best response assignment will depend on the achievement of both measurement and confirmation criteria.

For Participants with Measurable Disease (i.e., Target Disease)

Target Lesions	Non-Target Lesions	New Lesions	Overall Response	Best Overall Response when Confirmation is Required*
CR	CR	No	CR	≥4 wks. Confirmation**
CR	Non-CR/Non-PD	No	PR	≥4 wks. Confirmation**
CR	Not evaluated	No	PR	
PR	Non-CR/Non-PD/not evaluated	No	PR	
SD	Non-CR/Non-PD/not evaluated	No	SD	Documented at least once ≥4 wks. from baseline**
PD	Any	Yes or No	PD	no prior SD, PR or CR
Any	PD***	Yes or No	PD	
Any	Any	Yes	PD	

* See RECIST 1.1 manuscript for further details on what is evidence of a new lesion.
** Only for non-randomized trials with response as primary endpoint.
*** In exceptional circumstances, unequivocal progression in non-target lesions may be accepted as disease progression.
Note: Participants with a global deterioration of health status requiring discontinuation of treatment without objective evidence of disease progression at that time should be reported as "*symptomatic deterioration.*" Every effort should be made to document the objective progression even after discontinuation of treatment.

For Participants with Non-Measurable Disease (i.e., Non-Target Disease)

Non-Target Lesions	New Lesions	Overall Response
CR	No	CR
Non-CR/non-PD	No	Non-CR/non-PD*
Not all evaluated	No	not evaluated
Unequivocal PD	Yes or No	PD
Any	Yes	PD

* 'Non-CR/non-PD' is preferred over 'stable disease' for non-target disease since SD is increasingly used as an endpoint for assessment of efficacy in some trials so to assign this category when no lesions can be measured is not advised

6.3.5 Duration of Response

Duration of overall response: The duration of overall response is measured from the time measurement criteria are met for CR or PR (whichever is first recorded) until the first date that

recurrent or progressive disease is objectively documented (taking as reference for progressive disease the smallest measurements recorded since the treatment started).

The duration of overall CR is measured from the time measurement criteria are first met for CR until the first date that progressive disease is objectively documented.

Duration of stable disease: Stable disease is measured from the start of the treatment until the criteria for progression are met, taking as reference the smallest measurements recorded since the treatment started, including the baseline measurements.

6.4 Toxicity Criteria

The following adverse event management guidelines are intended to ensure the safety of each participant while on the study. The descriptions and grading scales found in the revised NCI Common Terminology Criteria for Adverse Events (CTCAE) version 4.0 will be utilized for AE reporting. All appropriate treatment areas should have access to a copy of the CTCAE version 4.0. A copy of the CTCAE version 4.0 can be downloaded from the CTEP web site (http://ctep.cancer.gov/protocolDevelopment/electronic_applications/ctc.htm#ctc_40).

7 NIH REPORTING REQUIREMENTS/DATA AND SAFETY MONITORING PLAN

7.1 Definitions

Please refer to definitions provided in Policy 801: Reporting Research Events found at: https://irbo.nih.gov/confluence/pages/viewpage.action?pageId=36241835#Policies&Guidance-800Series-ComplianceandResearchEventReportingRequirements.

7.2 OHSRP Office of Compliance and Training / IRB Reporting

7.2.1 Expedited Reporting

Please refer to the reporting requirements in Policy 801: Reporting Research Events and Policy 802 Non-Compliance Human Subjects Research found at: https://irbo.nih.gov/confluence/pages/viewpage.action?pageId=36241835#Policies&Guidance-800Series-ComplianceandResearchEventReportingRequirements. Note: Only IND Safety Reports that meet the definition of an unanticipated problem will need to be reported per these policies.

7.2.2 IRB Requirements for PI Reporting at Continuing Review

Please refer to the reporting requirements in Policy 801: Reporting Research Events found at: https://irbo.nih.gov/confluence/pages/viewpage.action?pageId=36241835#Policies&Guidance-800Series-ComplianceandResearchEventReportingRequirements.

7.3 NCI Clinical Director Reporting

Problems expeditiously reviewed by the OHSRP in the NIH eIRB system will also be reported to the NCI Clinical Director/designee; therefore, a separate submission for these reports is not necessary.

In addition to those reports, all deaths that occur within 30 days after receiving a research intervention should be reported via email unless they are due to progressive disease.

To report these deaths, please send an email describing the circumstances of the death to NCICCRQA@mail.nih.gov within one business day of learning of the death.

7.4 NIH Required Data and Safety Monitoring Plan

7.4.1 Principal Investigator/Research Team

The clinical research team will meet on a regular basis weekly when participants are being actively treated on the trial to discuss each participant. Decisions about dose level enrollment and dose escalation if applicable will be made based on the toxicity data from prior participants.

All data will be collected in a timely manner and reviewed by the principal investigator or a lead associate investigator. Events meeting requirements for expedited reporting as described in section **7.2.1** will be submitted within the appropriate timelines.

The principal investigator will review adverse event and response data on each participant to ensure safety and data accuracy. The principal investigator will personally conduct or supervise the investigation and provide appropriate delegation of responsibilities to other members of the research staff.

7.4.2 Central Reading of Scans

An independent central review (ICR) of all scans for HGSOC participants used in the assessment of tumors using RECIST 1.1 will be conducted. Copies of all imaging assessments including unscheduled visit scans will be collected on an ongoing basis and sent to the contract research organization appointed by Eli Lilly.

Prior radiation therapy reports for participants (at baseline) and information on any lesions that were biopsied to provide a tumor sample for study entry will be provided to allow the selection of appropriate target lesions.

The imaging scans will be reviewed by two independent radiologists using RECIST 1.1 criteria and will be adjudicated if required. For each participant, the ICR will define the overall visit response data (CR, PR, SD, PD or not evaluable [NE]) and the relevant scan dates for each time point (i.e. for visits where response or progression is/is not identified). If a participant has had a tumor assessment which cannot be evaluated then the participant will be assigned a visit response of NE (unless there is evidence of progression in which case the response will be assigned as PD).

The management of participants will be based solely upon the results of the RECIST assessment conducted by the Investigator. Results of this ICR will not be communicated to Investigators to make a clinical decision.

8 SPONSOR SAFETY REPORTING

8.1 DEFINITIONS

8.1.1 Adverse Event

Any untoward medical occurrence in a patient or clinical investigation subject administered a pharmaceutical product and which does not necessarily have a causal relationship with this treatment. An adverse event (AE) can therefore be any unfavorable and unintended sign (including an abnormal laboratory finding), symptom, or disease temporally associated with the use of a medicinal (investigational) product, whether or not related to the medicinal (investigational) product (ICH E6 (R2))

8.1.2 Serious Adverse Event (SAE)

An adverse event or suspected adverse reaction is considered serious if in the view of the investigator or the sponsor, it results in any of the following:

- Death,
- A life-threatening adverse event (see **8.1.3**)
- Inpatient hospitalization or prolongation of existing hospitalization
    - A hospitalization/admission that is pre-planned (i.e., elective or scheduled surgery arranged prior to the start of the study), a planned hospitalization for pre-existing condition, or a procedure required by the protocol, without a serious deterioration in health, is not considered a serious adverse event.
    - A hospitalization/admission that is solely driven by non-medical reasons (e.g., hospitalization for patient convenience) is not considered a serious adverse event.
    - Emergency room visits or stays in observation units that do not result in admission to the hospital would not be considered a serious adverse event. The reason for seeking medical care should be evaluated for meeting one of the other serious criteria.
- Persistent or significant incapacity or substantial disruption of the ability to conduct normal life functions
- A congenital anomaly/birth defect.
- Important medical events that may not result in death, be life-threatening, or require hospitalization may be considered a serious adverse drug experience when, based upon appropriate medical judgment, they may jeopardize the patient or subject and may require medical or surgical intervention to prevent one of the outcomes listed in this definition.

8.1.3 Life-threatening

An adverse event or suspected adverse reaction is considered "life-threatening" if, in the view of either the investigator or sponsor, its occurrence places the patient or subject at immediate risk of death. It does not include an adverse event or suspected adverse reaction that, had it occurred in a more severe form, might have caused death. (21CFR312.32)

8.1.4 Severity

The severity of each Adverse Event will be assessed utilizing the CTCAE version 4.0.

8.1.5 Relationship to Study Product

All AEs will have their relationship to study product assessed using the terms: related or not related.

- Related – There is a reasonable possibility that the study product caused the adverse event. Reasonable possibility means that there is evidence to suggest a causal relationship between the study product and the adverse event.

- Not Related – There is not a reasonable possibility that the administration of the study product caused the event.

8.2 Assessment of Safety Events

AE information collected will include event description, date of onset, assessment of severity and relationship to study product and alternate etiology (if not related to study product), date of resolution of the event, seriousness and outcome. The assessment of severity and relationship to the study product will be done only by those with the training and authority to make a diagnosis and listed on the Form FDA 1572 as the site principal investigator or sub-investigator. AEs occurring during the collection and reporting period will be documented appropriately regardless of relationship. AEs will be followed through resolution.

SAEs will be:

- Assessed for severity and relationship to study product and alternate etiology (if not related to study product) by a licensed study physician listed on the Form FDA 1572 as the site principal investigator or sub-investigator.
- Recorded on the appropriate SAE report form, the medical record and captured in the clinical database.
- Followed through resolution by a licensed study physician listed on the Form FDA 1572 as the site principal investigator or sub-investigator.

For timeframe of recording adverse events, please refer to section **6.1**. All serious adverse events recorded from the time of first investigational product administration must be reported to the sponsor.

8.3 Reporting of Serious Adverse Events

Any AE that meets protocol-defined serious criteria or meets the definition of Adverse Event of Special Interest that require expedited reporting must be submitted immediately (within 24 hours of awareness) to OSRO Safety using the CCR SAE report form.

All SAE reporting must include the elements described in **8.2**.

SAE reports will be submitted to the Center for Cancer Research (CCR) at: OSROSafety@mail.nih.gov and to the CCR PI and study coordinator. CCR SAE report form and instructions can be found at:
https://ccrod.cancer.gov/confluence/pages/viewpage.action?pageId=157942842

Following the assessment of the SAE by OSRO, other supporting documentation of the event may be requested by the OSRO Safety and should be provided as soon as possible.

8.4 Safety Reporting Criteria to the Pharmaceutical Collaborators

All events listed below must be reported in the defined timelines to OSROSafety@mail.nih.gov.

The CCR Office of Sponsor and Regulatory Oversight (OSRO) will send all reports to the manufacturer as described below.

Eli Lilly and Company will be notified via fax at 866-644-1697 or phone (800-545-5979) in a written IND Safety Report (MedWatch, Form 3500A) as soon as possible and no later than 24 hours of Investigator and/or Institution receiving notification of any serious adverse event experienced by a patient participating in the study and receiving study drug. To the extent possible, any AE whose severity and attribution require expedited submission of the AE report to the FDA will be sent to Eli Lilly and company in advance of report submission to the FDA.

The Investigative site must also indicate, either in the SAE report or the fax cover page, the causality of events in relation to each of the study medications and if the SAE is related to disease progression, as determined by the principal investigator. Any event or hospitalization that is unequivocally due to progression of disease, as determined by the PI, will not be reported as an SAE to the FDA, however should be communicated to Eli Lilly.

A cover page should accompany the MedWatch form indicating the following:

- LY2606368 Investigator Sponsored Study (ISS)
- The investigator IND number assigned by the FDA
- The investigator's name and address
- The trial name and Eli Lilly Reference number
- Causality

Follow-up information on SAEs must also be reported by the investigator to NCI and then Eli Lilly within 2 business days for death or life threatening or 7 business days for all SAEs for which serious criteria apply after initial receipt of the information.

If follow-up indicates a change in the SAE from serious to fatal or life threatening, this information also needs to be communicated to NCI and Eli Lilly within 1 business day. If follow-up indicates a change from serious to non-serious, this information needs to be communicated to NCI and Eli Lilly within 5 days.

All SAEs will be reported to Eli Lilly on a MedWatch form using the following guidelines:

- if there is more than one SAE listed on the MedWatch form, each should be bulleted or numbered
- causality for each SAE must be specified in the narrative section
- the date the site notified NCI and Eli Lilly of the SAE must be specified in the narrative section
- initial and follow-up MedWatch reports should be numbered either in the narrative section or in section G.7 of the MedWatch form

Once a MedWatch form is completed, it will be sent to Eli Lilly via fax at 1-866-644-1697.

8.5 Reporting Pregnancy

All required pregnancy reports/follow-up to OSRO will be submitted to: OSROSafety@mail.nih.gov and to the CCR PI and study coordinator. Forms and instructions can be found here: https://ccrod.cancer.gov/confluence/display/CCRCRO/Forms+and+Instructions

8.5.1 Maternal exposure

If a participant becomes pregnant during the course of the study, the study treatment should be discontinued immediately, and the pregnancy reported to the Sponsor no later than 24 hours of when the Investigator becomes aware of it. The Investigator should notify the Sponsor no later than 24 hours of when the outcome of the Pregnancy becomes known.

Pregnancy itself is not regarded as an SAE. However, congenital abnormalities or birth defects and spontaneous miscarriages that meet serious criteria (**8.1.2**) should be reported as SAEs.

The outcome of all pregnancies should be followed up and documented.

8.5.2 Paternal exposure

Male participants should refrain from fathering a child or donating sperm during the study and for 4 months after the last dose of LY2606368.

Pregnancy of the participant's partner is not considered to be an AE. The outcome of all pregnancies (spontaneous miscarriage, elective termination, ectopic pregnancy, normal birth, or congenital abnormality) occurring from the date of the first dose until 4 months after the last dose should, if possible, be followed up and documented. Pregnant partners may be offered the opportunity to participate in an institutional pregnancy registry protocol (e.g., the NIH IRP

pregnancy registry study) to provide data about the outcome of the pregnancy for safety reporting purposes.

8.6 Regulatory Reporting for Studies Conducted Under CCR-Sponsored IND

Following notification from the investigator, CCR, the IND sponsor, will report any suspected adverse reaction that is both serious and unexpected. CCR will report an AE as a suspected adverse reaction only if there is evidence to suggest a causal relationship between the study product and the adverse event. CCR will notify FDA and all participating investigators (i.e., all investigators to whom the sponsor is providing drug under its INDs or under any investigator's IND) in an IND safety report of potential serious risks from clinical trials or any other source, as soon as possible, in accordance to 21 CFR Part 312.32.

All serious events will be reported to the FDA at least annually in a summary format.

9 CLINICAL MONITORING

As a sponsor for clinical trials, FDA regulations require the CCR to maintain a monitoring program. The CCR's program allows for confirmation of: study data, specifically data that could affect the interpretation of primary and secondary study endpoints; adherence to the protocol, regulations, ICH E6, and SOPs; and human subjects protection. This is done through independent verification of study data with source documentation focusing on:

- Informed consent process
- Eligibility confirmation
- Drug administration and accountability
- Adverse events monitoring
- Response assessment.

This trial will be monitored by personnel employed by a CCR contractor. Monitors are qualified by training and experience to monitor the progress of clinical trials. Personnel monitoring this study will not be affiliated in any way with the trial conduct.

10 STATISTICAL CONSIDERATIONS

The primary objective of this trial is to establish whether LY2606368 is associated with at least a minimal response rate in participants with BRCA mutation-associated breast or ovarian cancer (cohort 1), HGSOC at low genetic risk (cohort 2), TNBC at low genetic risk (cohort 3), and mCRPC (cohort 4). Note that, effective with amendment I (version date 4/24/2017), Cohort 4 (mCRPC) was closed due to slow accrual. In each of these cohorts, the study will be conducted using an optimal two-stage phase II trial design[43] in order to rule out an unacceptably low 5% overall response rate (p0=0.05) in favor of an improved response rate of 25% (p1=0.25). With

alpha=0.10 (probability of accepting a poor treatment=0.10) and beta = 0.10 (probability of rejecting a good treatment=0.10), each cohort of this study will initially enroll 9 evaluable participants, and if 0 of the 9 have a response, then no further participants will be accrued in that cohort. If 1 or more of the first 9 participants in a cohort has a response, then accrual would continue until a total of 24 participants have been enrolled in that cohort. As it may take several weeks to determine if a participant has experienced a response, a temporary pause in the accrual to the trial for that cohort may be necessary to ensure that enrollment to the second stage is warranted. If there are 1 to 2 responses in 24 participants, this would be an uninterestingly low response rate. If there were 3 or more responses in 24 participants (12.5%), this would be sufficiently interesting to warrant further study in later trials. Under the null hypothesis (5% response rate), the probability of early termination is 63.0%.

Based on the promising results from the HGSOC at low genetic risk cohort 2, the cohort 5 of recurrent platinum-resistant HGSOC will use a single stage design, with an early stopping rule for substantially lower than expected efficacy. A total of 36 evaluable participants will be enrolled in the new cohort in order to have 89% power to rule out a 20% response rate in favor of an alternative response rate of 45%, using a two-tailed 0.05 significance level exact binomial test. In practice, a 95% confidence interval will be formed about the reported proportion of responders, and those with clinical benefit, among the 36 evaluable participants to describe the results obtained. If there are 13 (or more) responders out of 36 evaluable participants (36.1%), the exact two-sided 95% confidence interval about the proportion responding extends from 20.8% to 53.8%, thus ruling out 20% and demonstrating consistency with a result of at least 45%. An early stopping rule will be implemented as follows: if after 10 evaluable participants have been enrolled, there are 0 to 1 responders, then no further participants will be accrued since the upper one-sided 90% confidence bound on 1/10 is 0.337. This would mean that there is a reasonably high probability that the true response rate is below 1/3, obtained previously, and thus the results may not be as promising as expected.

Also, based on the promising results from the HGSOC at low genetic risk cohort 2, the new cohort 6 of non-BRCA mutated HGSOC participants without biopsiable disease will use a single stage design, with an early stopping rule for substantially lower than expected efficacy. A total of 35 evaluable participants will be enrolled in the new cohort in order to have 88% power to rule out a 15% response rate in favor of an alternative response rate of 40%, using a two-tailed 0.05 significance level exact binomial test. In practice, a 95% confidence interval will be formed about the reported proportion of responders, and those with clinical benefit, among the 35 evaluable participants to describe the results obtained. If there are 11 (or more) responders out of 35 evaluable participants (31.4%), the exact two-sided 95% confidence interval about the proportion responding extends from 16.9% to 49.3%, thus ruling out 15% and demonstrating consistency with a result of at least 40%. An early stopping rule also will be implemented as follows: if after 10 evaluable participants have been enrolled, there are 0 to 1 responders, then no further participants will be accrued since the upper one-sided 90% confidence bound on 1/10 is 0.337. This would mean that there is a reasonably high probability that the true response rate is below 1/3, obtained previously, and thus the results may not be as promising as expected.

The primary analyses will be done separately by disease cohort, although the response rates may not differ substantially by type of disease. In addition, if results are sufficiently similar, the participants from all four-disease types may be evaluated together in a single cohort in an exploratory fashion. In each cohort, participants will be divided into groups based on whether they responded (PR+CR) or not, or experienced at least some clinical benefit (CR+PR+SD) or not, and when any biologic or biochemical parameters are obtained, the changes in these parameters from baseline will be compared between the two groups using a Wilcoxon rank sum test. As these will be considered exploratory analyses, the results will not be adjusted for any multiple comparisons but will be considered in the context of the hypothesis generating nature of the evaluations. PFS will be estimated using a Kaplan-Meier curve based on all evaluable participants, as well as separately by cohort. The PFS at selected points will also be reported, along with associated 80% and 95% confidence intervals. Safety and toxicity in this population will be determined by tabulating the toxicity grades identified in the participants treated on the trial, by cohort, and describing the findings as needed.

Other secondary exploratory endpoint for the cohorts 1-3 and 5 will focus on the ability of biochemical changes in the DNA damage repair and cell cycle checkpoint pathways to be associated with response. This will be evaluated in a descriptive fashion. For cohort 5, all participants will undergo a biopsy at baseline. It is expected that 12 participants may be clinical responders and that perhaps 6 to 12 more may have clinical benefit. Beginning at the first time after which a tumor grows >15% over baseline, among those who have responded or shown clinical benefit by 4 months, a second biopsy at progression will be obtained to provide potential resistance mechanisms. For a potential single protein or gene biomarker, with 16 paired biopsies available, there would be 80% power to detect a change from baseline with an effect size of 0.75 (0.75 SD of the change) which would be significant at the 0.05 two-tailed significance level using a paired t-test. Similarly, 15 paired biopsies would result in 94% power to detect a change with a 1.0 SD effect size, while 10 paired biopsies would have 80% power for a 1.0 SD effect size, both with a 0.05 two-tailed significance level. If there were 3 total potential protein or gene biomarkers instead of one, 22 paired biopsies would yield 81% power for a 0.75 SD effect size using a two-tailed 0.017 significance level paired t-test in order to permit adequate participants for a very strict Bonferroni adjustment, holding the three tests to an overall 0.05 significance level, while 14 paired biopsies would have 81% power for detecting a 1.0 SD effect size, also at the 0.017 significance level. In practice, a Wilcoxon signed rank test will be used instead of a paired t-test if the changes are not normally distributed ($p<0.05$ by a Shapiro-Wilks test). Also, if multiple markers are being evaluated, a Hochberg adjustment may be used instead of an overly conservative Bonferroni correction.

In the participants enrolled in cohort 6, analyses of biomarkers will be undertaken based on blood samples. As indicated in section 5.1.4, a variety of molecular markers will be collected and evaluated to determine if there are changes in the values after treatment vs. at baseline. For each of the parameters mentioned in section 5.1.4 (CTCs, immune subsets such as Tregs, MDSC, CD8 T+ cells, PD-1, TIM3, CTLA-4, and/or CD40, and γH2AX and MRE11), values will be obtained at baseline and at C1D15. The change in values between the two timepoints will be determined and assessed for statistical significance in an exploratory fashion. If there are 30

participants with paired values, for a given biomarker, there would be 90% power to detect a difference from baseline with an effect size of 0.75 (0.75 SD of the difference), with a 0.01 two-sided significance level, using a paired t-test. In practice, a Wilcoxon signed rank test will be used if the differences are not normally distributed ($p<0.05$ by a Shapiro-Wilks test). Also, as this will be an exploratory analysis, the significance of the changes will be reported without any formal adjustment for multiple comparisons, but in the context of the number of tests performed.

Several of the molecular markers may also be compared between responders and non-responders in cohort 6 in an exploratory fashion. Assuming that 1/3 of the participants are responders and 2/3 are non-responders, and data are available on 30 of the participants in cohort 6, with data on changes from baseline to cycle 1 day 15 from 10 responders and 20 non-responders, for a given parameter, there would be 87% power to detect a difference between the two groups with an effect size of 1.25 (1.25 SD of the difference), with a 0.05 two-sided significance level, using a two-group t-test. In practice, a Wilcoxon rank sum test will be used if the differences are not normally distributed ($p<0.05$ by a Shapiro-Wilks test). Also, as this will be an exploratory analysis, the significance of the changes will be reported without any formal adjustment for multiple comparisons but in the context of the number of tests performed.

It is expected that 35-40 participants per year may enroll onto this trial. In order to have 143 total evaluable participants, it is anticipated that 4-4.5 years may be required for accrual. To allow for a small number of non-evaluable participants, the accrual ceiling will be set at 153 participants.

- **NOTE:** Effective with 4/17/2020, the study was closed for enrollment because Eli Lilly withdrew sponsorship for drug supplies on 2/19/2020. Therefore, cohorts 5 and 6 were prematurely closed for accrual before enrolling the planned numbers. To have a reasonable number of data analysis, the participants from the cohorts 5 and 6 may be reported together as a combined dataset if the results are sufficiently similar between the cohorts 5 and 6.

11 COLLABORATIVE AGREEMENTS

11.1 COOPERATIVE RESEARCH AND DEVELOPMENT AGREEMENT (CRADA)

11.1.1 Eli Lilly and Company

The investigational study agent, LY2606368, is provided by Eli Lilly and Company under a Collaborative Agreement [Cooperative Research and Development Agreement (CRADA #02915)].

12 HUMAN SUBJECTS PROTECTIONS

12.1 Rationale For Subject Selection

Subjects from all racial/ethnic groups are eligible for this study if they meet the eligibility criteria. To date, there is no information that suggests that differences in drug metabolism or disease response would be expected in one group compared to another. Efforts will be made to extend accrual to a representative population, but in this preliminary study, a balance must be struck between patient safety considerations and limitations on the number of individuals exposed to potentially toxic and/or ineffective treatments on the one hand and the need to explore ethnic aspects of clinical research on the other hand. If differences in outcome that correlate to ethnic identity are noted, accrual may be expanded or a follow-up study may be written to investigate those differences more fully. This study will be recruited through internal referral, our local physician referral base, and through Cancer Hotline information.

12.2 Participation of Children

Subjects under 18 are excluded because the incidence of breast or ovarian cancer in this population is extremely rare. The study medication is also investigational and has not been studied in pediatric populations.

12.3 Participation of Subjects Unable to Give Consent

Adults unable to provide consent are excluded from enrolling in the protocol. However, it is possible that subjects enrolled in the protocol may permanently lose the capacity to consent for themselves during the course of this study. In the event this occurs, the subjects will remain in the study because, although the risk of participation is greater than minimal due to the potential toxicities of treatment, there is a prospect for direct benefit due to the potential for reduction of tumor burden. All subjects $\geq$ age 18 will be offered the opportunity to fill in their wishes for research and care, and assign a substitute decision maker on the "NIH Advance Directive for Health Care and Medical Research Participation" form so that another person can make decisions about their medical care in the event that they become incapacitated or cognitively impaired during the course of the study.

Note: The PI or AI will contact the NIH Ability to Consent Assessment Team (ACAT) for evaluation to assess ongoing capacity of the subjects and to identify an LAR, as needed.

Please see section **12.6.1** for consent procedure.

12.4 Evaluation of Benefits and Risks/Discomforts

Subjects enrolled on this study may receive direct benefit from participating in this protocol. LY2606368 demonstrated inhibition of tumor growth in multiple-tumor xenograft models as a single agent and in combination with gemcitabine. LY2606368, formulated in Captisol® for intravenous (IV) administration, appears to have a high volume of distribution, which indicates a wide distribution into tissues and organs. The most common SAEs deemed related to

LY2606368 treatment were hematologic toxicities or complications resulting from neutropenia, leucopenia, or thrombocytopenia.

12.4.1 Risks of Radiation

The study will involve radiation from the following sources:

- Up to 3 CT scans for the collection of biopsies
- Up to 7 CT scans per year for disease assessment

Subjects in this study may be exposed to approximately 10.1 rem. This amount is more than would be expected from everyday background radiation. Being exposed to excess radiation can increase the risk of cancer. The risk of getting cancer from the radiation exposure in this study is 1 out of 100 (1%) and of getting a fatal cancer is 0.5 out of 100 (0.5%).

12.4.2 Risk of Treatment: Details of the risk of drug therapy are detailed in section **13.**

12.4.3 Blood draws: Side effects of blood draws include pain and bruising, lightheadedness, and rarely, fainting.

12.4.4 CT scans: In addition to the radiation risks discussed above, risks associated with CT scans are allergic reaction to and kidney damage from the contrast dye, nausea, vomiting, and anxiety.

12.4.5 Biopsies: The non-radiation risks associated with the biopsy procedure include pain, swelling and/or bleeding at the biopsy site; infection; and allergic reaction to the local anesthetic used.

12.5 RISKS/BENEFITS ANALYSIS

This dose and schedule of 105mg/m^2 was selected based on the nonclinical PK/PD model, the clinical PK data demonstrating that the LY2606368 systemic exposure at the Maximum Tolerated Dose (MTD) for each schedule of administration is in the range predicted for maximal tumor response, the human PD simulations that predict the average %pChk1 inhibition at the MTD for each schedule of administration achieves the requirements described for the minimum tumor response, the similar safety profile for each schedule, and the increased patient convenience.

- Adult subjects: More than minimal risk and the prospect of direct benefit.
- Adults unable to consent: Category B - Research involving greater than minimal risk but presenting the prospect of direct benefit to the individual subjects.

- Protocol Specific Justification for Risk Assessment: For adults who lose the capacity to consent the risk is greater than minimal due to the potential toxicities of treatment, but there is prospect for direct benefit due to the potential for reduction of tumor burden.

12.6 CONSENT PROCESS AND DOCUMENTATION

The informed consent document will be provided to the participant or consent designee(s) as applicable for review prior to consenting. A designated study investigator will carefully explain the procedures and tests involved in this study, and the associated risks, discomforts and benefits. In order to minimize potential coercion, as much time as is needed to review the document will be given, including an opportunity to discuss it with friends, family members and/or other advisors, and to ask questions of any designated study investigator. A signed informed consent document will be obtained prior to entry onto the study.

The initial consent process as well as re-consent, when required, may take place in person or remotely (e.g., via telephone or other NIH approved remote platforms) per discretion of the designated study investigator and with the agreement of the participant/consent designee(s).

Whether in person or remote, the privacy of the subject will be maintained. Consenting investigators (and participant/consent designee, when in person) will be located in a private area (e.g., clinic consult room). When consent is conducted remotely, the participant/consent designee will be informed of the private nature of the discussion and will be encouraged to relocate to a more private setting if needed.

12.6.1 Consent Process for Adults Who Lack Capacity to Consent to Research Participation

For participants addressed in section **12.3**, an LAR will be identified consistent with Policy 403 and informed consent obtained from the LAR, as described in Section **12.6**.

13 REGULATORY AND OPERATIONAL CONSIDERATIONS

13.1 STUDY DISCONTINUATION AND CLOSURE

This study may be temporarily suspended or prematurely terminated if there is sufficient reasonable cause. Written notification, documenting the reason for study suspension or termination, will be provided by the suspending or terminating party to study participants, investigator, funding agency, the Investigational New Drug (IND) sponsor, and regulatory authorities. If the study is prematurely terminated or suspended, the Principal Investigator (PI) will promptly inform study participants, the Institutional Review Board (IRB), and the sponsor, and will provide the reason(s) for the termination or suspension. Study participants will be contacted, as applicable, and be informed of changes to the study visit schedule.

Circumstances that may warrant termination or suspension include, but are not limited to:

- Determination of unexpected, significant, or unacceptable risk to participants
- Demonstration of efficacy that would warrant stopping

- Insufficient compliance to protocol requirements
- Data that are not sufficiently complete and/or evaluable
- Determination that the primary endpoint has been met
- Determination of futility

Study may resume once concerns about safety, protocol compliance, and data quality are addressed, and satisfy the sponsor, IRB and as applicable, Food and Drug Administration (FDA).

13.2 QUALITY ASSURANCE AND QUALITY CONTROL

Each clinical site will perform internal quality management of study conduct, data and biological specimen collection, documentation and completion. An individualized quality management plan will be developed to describe a site's quality management.

Quality control (QC) procedures will be implemented beginning with the data entry system and data QC checks that will be run on the database will be generated. Any missing data or data anomalies will be communicated to the site(s) for clarification/resolution.

Following written Standard Operating Procedures (SOPs), the monitors will verify that the clinical trial is conducted and data are generated and biological specimens are collected, documented (recorded), and reported in compliance with the protocol, International Council on Harmonisation Good Clinical Practice (ICH GCP), and applicable regulatory requirements (e.g., Good Laboratory Practices (GLP), Good Manufacturing Practices (GMP)).

The investigational site will provide direct access to all trial related sites, source data/documents, and reports for the purpose of monitoring and auditing by the sponsor, and inspection by local and regulatory authorities.

13.3 CONFLICT OF INTEREST POLICY

The independence of this study from any actual or perceived influence, such as by the pharmaceutical industry, is critical. Therefore, any actual conflict of interest of persons who have a role in the design, conduct, analysis, publication, or any aspect of this trial will be disclosed and managed. Furthermore, persons who have a perceived conflict of interest will be required to have such conflicts managed in a way that is appropriate to their participation in the design and conduct of this trial. The study leadership in conjunction with the National Cancer Institute has established policies and procedures for all study group members to disclose all conflicts of interest and will establish a mechanism for the management of all reported dualities of interest.

13.4 CONFIDENTIALITY AND PRIVACY

Participant confidentiality and privacy is strictly held in trust by the participating investigators, their staff, and the sponsor(s). This confidentiality is extended to cover testing of biological

samples and genetic tests in addition to the clinical information relating to participants. Therefore, the study protocol, documentation, data, and all other information generated will be held in strict confidence. No information concerning the study or the data will be released to any unauthorized third party without prior written approval of the sponsor.

All research activities will be conducted in as private a setting as possible.

The study monitor, other authorized representatives of the sponsor, representatives of the Institutional Review Board (IRB), and/or regulatory agencies may inspect all documents and records required to be maintained by the investigator, including but not limited to, medical records (office, clinic, or hospital) and pharmacy records for the participants in this study. The clinical study site will permit access to such records.

The study participant's contact information will be securely stored at the/each clinical site for internal use during the study. At the end of the study, all records will continue to be kept in a secure location for as long a period as dictated by the reviewing IRB, Institutional policies, or sponsor requirements.

Study participant research data, which is for purposes of statistical analysis and scientific reporting, will be stored at the NCI CCR. This will not include the participant's contact or identifying information. Rather, individual participants and their research data will be identified by a unique study identification number. The study data entry and study management systems used by the clinical site and by NCI CCR research staff will be secured and password protected. At the end of the study, all study databases will be archived at the NIH.

To further protect the privacy of study participants, a Certificate of Confidentiality has been issued by the National Institutes of Health (NIH). This certificate protects identifiable research information from forced disclosure. It allows the investigator and others who have access to research records to refuse to disclose identifying information on research participation in any civil, criminal, administrative, legislative, or other proceeding, whether at the federal, state, or local level. By protecting researchers and institutions from being compelled to disclose information that would identify research participants, Certificates of Confidentiality help achieve the research objectives and promote participation in studies by helping assure confidentiality and privacy to participants.

14 PHARMACEUTICAL INFORMATION

14.1 LY2606368

14.1.1 Source

LY2606368 is an investigational agent (IND #123172) and will be supplied to the NIH Clinical Center by Eli Lilly and Company (Indianapolis, IN).

14.1.2 Toxicity

As of 04 April 2016, data were available from 210 patients who were treated with LY2606368 on Schedule 1 or 2. PK data are available from 194 patients from 3 ongoing clinical trials (studies JTJA, JTJF, and JTJK). Study JTJA is the first-in-human phase 1 study of LY2606368.

Hematologic Toxicity

Neutropenia is the most frequent toxicity in study JTJA and has been observed at all doses and schedules of LY2606368.

Prophylactic granulocyte colony stimulating factor (G-CSF) use is permitted as indicated in **3.3.1.1.**

Non-Hematologic Toxicity

The only non-hematologic treatment-emergent AEs (TEAEs) occurring in greater than 10% of patients and deemed by the investigators to be related to LY2606368 treatment (combined Schedule 1 and Schedule 2) were nausea (15%, grade 1: 14%, grade 2: 1%) and fatigue (29%, grade 1/2: 27%, grade 3: 2%). Related non-hematologic TEAEs occurring in 5% to 10% of patients included: anorexia (8%), headache (7%), oral mucositis (7%), diarrhea (5%), and vomiting (5%). All of these were grade 1 or 2.

Cardiovascular Effects
Please see **3.3.2** QTc Prolongation.

No corrected QT interval (QTc) prolongation was observed in dogs at doses up to 10 mg/kg (200 mg/m^2). The mean maximum plasma concentration (C_{max}) levels at the dose at which QTc prolongation was seen in dogs (6950 and 5862 ng/mL at 40 mg/kg, or 800 mg/m^2, given as a 1- or 2-hour infusion, respectively) are significantly greater than the largest mean LY2606368 C_{max} in humans at the MTD of 40 mg/m^2 administered on days 1 to 3 every 14 days (Schedule 1) (cohort mean = 347 ng/mL on day 3 of cycle 1 from Part A in the current phase I study [dose escalation]), and the largest mean LY2606368 C_{max} in humans at the MTD of 105 mg/m^2 administered on day 1 every 14 days (cohort mean = 867 ng/mL on day 1 of cycle 2 from Part A in the current phase I study).

As of April 2016, in study JTJA (LY2606368 monotherapy), no prolongations in average QTcF interval to >480 ms or of ≥ 60 ms from baseline were observed with LY2606368 monotherapy (n=101) at any time point. Eight patients (7.9%) had a QTcF change of ≥ 30 ms from baseline. None of these changes exceeded 39 ms, and none were observed to be associated with AEs. In study JTJK (LY2606368 monotherapy in Japanese patients), two of 10 patients had grade 2 QTcF prolongation (both were on 80mg/m^2; one with baseline QTcF 468 ms, changed to post-treatment QTcF 511 ms with grade 1 atrial fibrillation, and no known history of cardiovascular issues and normal electrolytes, deemed DLT; another with baseline QTcF 432 ms but had

nonspecific T wave changes, precluding accurate measurement of the QT interval. No other cardiac AEs were reported for these patients. A thorough QTc study has not yet been conducted to date for Japanese patients.

On days when LY2606368 is administered, patients should avoid taking any concomitant medications within 12 hours before and after the infusion of LY2606368 that are known or suspected to cause prolonged QTc or Torsades de Points and, if possible, alternative agents should be considered.
Because LY2606368 interacts with multiple 5-hydroxytryptamine (5-HT) receptors, patients should be monitored closely for any signs and symptoms of serotonin syndrome. If observed, appropriate treatment (eg, cyproheptadine or octreotide) may be used as a treatment.

Additional findings in animals that also may occur in humans at clinically relevant doses include abnormal stools, skin reddening, decreased body weight and/or food consumption, and tremors. In addition, 8 of 20 dogs given doses of LY2606368 above the MTD were sacrificed for humane reasons prior to scheduled termination. The primary clinical signs associated with the declining condition of these animals were consistent with gastrointestinal (GI) injury and dehydration.

Experience with concurrent radiation therapy (RT) and LY2606368

There is an ongoing study with LY2606368 in combination with either cisplatin/RT or cetuximab/RT in patients with locally advanced HNSCC (study JTJI). As of August 2017, 19 patients have been dosed at LY2606368 doses of 20 mg/m^2 or 40 mg/m^2 in combination with IMRT – 2.0 Gy/day fractions (daily x 5 for 7 weeks)- LY2606368 is still given biweekly. Validated data is available from 14 patients (Study JTJI, **Table 7** and below). The toxicities have been consistent with either radiation, LY2606368, or cetuximab/cisplatin. During palliative radiation, a break in LY2606368 therapy is allowed not to overlap the therapies. Currently, the estimated median terminal half-life of LY2606368 is 27 hours.

Table 7 Treatment-Emergent Adverse Events Related to Study Treatment Occurring in ≥2 Patients in Each Treatment Group in Study I4D-MC-JTJI

Preferred Term	Prexasertib with Cisplatin and Radiation (N=7) n (%)		Prexasertib with Cetuximab and Radiation (N=7) n (%)		Total (N=14) n (%)	
	All Grades	Grade 3/4/5	All Grades	Grade 3/4/5	All Grades	Grade 3/4/5
Radiation skin injury	5 (71.4)	0	6 (85.7)	2 (28.6)	11 (78.6)	2 (14.3)
Anaemia	5 (71.4)	1 (14.3)	4 (57.1)	0	9 (64.3)	1 (7.1)
Dry mouth	4 (57.1)	0	5 (71.4)	0	9 (64.3)	0
Stomatitis	3 (42.9)	2 (28.6)	6 (85.7)	4 (57.1)	9 (64.3)	6 (42.9)
Platelet count decreased	6 (85.7)	0	3 (42.9)	0	9 (64.3)	0
Neutrophil count decreased	6 (85.7)	5 (71.4)	2 (28.6)	2 (28.6)	8 (57.1)	7 (50.0)
Dysphagia	3 (42.9)	1 (14.3)	4 (57.1)	2 (28.6)	7 (50.0)	3 (21.4)
Weight decreased	2 (28.6)	0	5 (71.4)	0	7 (50.0)	0
Nausea	4 (57.1)	0	2 (28.6)	0	6 (42.9)	0
Dermatitis acneiform	0	0	5 (71.4)	4 (57.1)	5 (35.7)	4 (28.6)
Febrile neutropenia	3 (42.9)	1 (14.3)	1 (14.3)	1 (14.3)	4 (28.6)	2 (14.3)
Sinus tachycardia	2 (28.6)	0	2 (28.6)	0	4 (28.6)	0
Hyponatraemia	3 (42.9)	1 (14.3)	1 (14.3)	0	4 (28.6)	1 (7.1)
Aphasia	2 (28.6)	2 (28.6)	2 (28.6)	1 (14.3)	4 (28.6)	3 (21.4)
Vomiting	3 (42.9)	0	1 (14.3)	0	4 (28.6)	0
Fatigue	2 (28.6)	0	2 (28.6)	0	4 (28.6)	0
Mucosal infection	2 (28.6)	0	2 (28.6)	0	4 (28.6)	0
Alanine aminotransferase increased	2 (28.6)	0	2 (28.6)	0	4 (28.6)	0
Aspartate aminotransferase increased	2 (28.6)	0	2 (28.6)	0	4 (28.6)	0
Blood creatinine increased	2 (28.6)	0	1 (14.3)	0	3 (21.4)	0
White blood cell count decreased	2 (28.6)	0	1 (14.3)	0	3 (21.4)	0
Tinnitus	2 (28.6)	0	0	0	2 (14.3)	0
Lymphocyte count decreased	2 (28.6)	0	0	0	2 (14.3)	0
Dyspepsia	0	0	2 (28.6)	0	2 (14.3)	0
Infusion related reaction	0	0	2 (28.6)	0	2 (14.3)	0
Headache	0	0	2 (28.6)	0	2 (14.3)	0

Source: /lillyce/prd/ly2606368/cibjan2017/output/i4d_mc_jtji/smteaeptsoc and smteaeptsocmax.

14.1.3 Formulation and preparation

The drug product, LY2606368 for injection, is supplied for clinical trial use as a lyophilized, yellow to white solid, in glass vials and is composed of LY2606368 mesylate monohydrate and the inactive ingredient Captisol®. Each vial contains 20 mg or 40 mg of the base compound LY2606368. The vial contains a 5% excess to facilitate the withdrawal of the label amount, 20 mg/vial or 40 mg/vial for application with an appropriate device, such as an infusion set.

LY2606368 will be infused through a low-sorbing, polyolefin-lined administration set with in-line 0.2-micrometer polyethersulfone filter. All components must be DEHP-free.

Patients will receive LY2606368 at the RP2D of 105 mg/m^2 IV once every 14 days of a 28-day cycle.
Reconstituting the vial contents with 10 mL of water gives a clear yellow solution with a concentration of 2 mg/mL of LY2606368, pH 4 to 7, and an osmotic pressure ratio of 344 mOsm.

14.1.4 Stability and Storage

The drug product is stable when stored at room temperature.
The reconstituted formulation is stable for at least 24 hours at room temperature; however, since the reconstituted drug product does not contain a preservative, the unused solution must be discarded after 12 hours.

14.1.5 Administration procedures

LY2606368 dose MAY be recalculated during repeated treatment cycles as a function of minor changes in body weight measurements through time. Dose recalculation during repeated treatment cycles is REQUIRED only when a patient's weight changes +/- 10% or more from their body weight measured just before starting study treatment (baseline weight).

LY2606368 is administered as an IV infusion over 1hour (+/-10min) using a central or free flowing peripheral IV line with an appropriate filter. Subsequent infusion may be extended if infusion over 1hr was not well tolerated, including but not limited to grade 1 facial flushing. LY2606368 should not come in contact with normal saline. The patient's vascular access device should be flushed with dextrose 5% injection (D5W) before and after LY2606368 administration.

The reconstituted drug product from the vials should be diluted with Dextrose 5% Injection (D5W) to a final volume of no more than 250 ml. If less than 250 ml is used, the concentration of LY2606368 must be maintained within a range of 0.08 mg/mL to 1.60 mg/mL of LY2606368.

14.1.6 Incompatibilities

LY2606368 is incompatible with solutions containing saline or lactated Ringer and must not be mixed or administered simultaneously with other drugs through the same infusion line.

15 REFERENCES

1. Moynahan ME, Chiu JW, Koller BH, Jasin M. Brca1 controls homology-directed DNA repair. Molecular cell 1999;4:511-8.
2. Turner N, Tutt A, Ashworth A. Hallmarks of 'BRCAness' in sporadic cancers. Nature reviews Cancer 2004;4:814-9.
3. Press JZ, De Luca A, Boyd N, et al. Ovarian carcinomas with genetic and epigenetic BRCA1 loss have distinct molecular abnormalities. BMC cancer 2008;8:17.
4. Integrated genomic analyses of ovarian carcinoma. Nature 2011;474:609-15.
5. Gallagher DJ, Konner JA, Bell-McGuinn KM, et al. Survival in epithelial ovarian cancer: a multivariate analysis incorporating BRCA mutation status and platinum sensitivity. Annals of oncology : official journal of the European Society for Medical Oncology / ESMO 2011;22:1127-32.
6. Safra T, Borgato L, Nicoletto MO, et al. BRCA mutation status and determinant of outcome in women with recurrent epithelial ovarian cancer treated with pegylated liposomal doxorubicin. Molecular cancer therapeutics 2011;10:2000-7.
7. Alsop K, Fereday S, Meldrum C, et al. BRCA mutation frequency and patterns of treatment response in BRCA mutation-positive women with ovarian cancer: a report from the Australian Ovarian Cancer Study Group. Journal of clinical oncology : official journal of the American Society of Clinical Oncology 2012;30:2654-63.
8. Long KC, Kauff ND. Hereditary ovarian cancer: recent molecular insights and their impact on screening strategies. Current opinion in oncology 2011;23:526-30.
9. Walsh T, Casadei S, Lee MK, et al. Mutations in 12 genes for inherited ovarian, fallopian tube, and peritoneal carcinoma identified by massively parallel sequencing. Proceedings of the National Academy of Sciences of the United States of America 2011;108:18032-7.
10. McCabe N, Turner NC, Lord CJ, et al. Deficiency in the repair of DNA damage by homologous recombination and sensitivity to poly(ADP-ribose) polymerase inhibition. Cancer research 2006;66:8109-15.
11. Konstantinopoulos PA, Spentzos D, Karlan BY, et al. Gene expression profile of BRCAness that correlates with responsiveness to chemotherapy and with outcome in patients with epithelial ovarian cancer. Journal of clinical oncology : official journal of the American Society of Clinical Oncology 2010;28:3555-61.
12. Carrassa L, Damia G. Unleashing Chk1 in cancer therapy. Cell Cycle 2011;10:2121-8.
13. Robinson D, Van Allen EM, Wu YM, et al. Integrative clinical genomics of advanced prostate cancer. Cell 2015;161:1215-28.
14. Beltran H, Yelensky R, Frampton GM, et al. Targeted next-generation sequencing of advanced prostate cancer identifies potential therapeutic targets and disease heterogeneity. European urology 2013;63:920-6.
15. Dai Y, Grant S. Targeting Chk1 in the replicative stress response. Cell Cycle 2010;9:1025.
16. Comprehensive molecular portraits of human breast tumours. Nature 2012;490:61-70.

17. Ma CX, Janetka JW, Piwnica-Worms H. Death by releasing the breaks: CHK1 inhibitors as cancer therapeutics. Trends in molecular medicine 2011;17:88-96.
18. Maya-Mendoza A, Petermann E, Gillespie DA, Caldecott KW, Jackson DA. Chk1 regulates the density of active replication origins during the vertebrate S phase. The EMBO journal 2007;26:2719-31.
19. Bucher N, Britten CD. G2 checkpoint abrogation and checkpoint kinase-1 targeting in the treatment of cancer. British journal of cancer 2008;98:523-8.
20. Zachos G, Black EJ, Walker M, et al. Chk1 is required for spindle checkpoint function. Developmental cell 2007;12:247-60.
21. Farmer H, McCabe N, Lord CJ, et al. Targeting the DNA repair defect in BRCA mutant cells as a therapeutic strategy. Nature 2005;434:917-21.
22. Bryant HE, Schultz N, Thomas HD, et al. Specific killing of BRCA2-deficient tumours with inhibitors of poly(ADP-ribose) polymerase. Nature 2005;434:913-7.
23. Fong PC, Boss DS, Yap TA, et al. Inhibition of poly(ADP-ribose) polymerase in tumors from BRCA mutation carriers. The New England journal of medicine 2009;361:123-34.
24. Deng CX. BRCA1: cell cycle checkpoint, genetic instability, DNA damage response and cancer evolution. Nucleic acids research 2006;34:1416-26.
25. Ashwell S, Zabludoff S. DNA damage detection and repair pathways--recent advances with inhibitors of checkpoint kinases in cancer therapy. Clinical cancer research : an official journal of the American Association for Cancer Research 2008;14:4032-7.
26. Yarden RI, Pardo-Reoyo S, Sgagias M, Cowan KH, Brody LC. BRCA1 regulates the G2/M checkpoint by activating Chk1 kinase upon DNA damage. Nature genetics 2002;30:285-9.
27. Samuel C. McNeely TFB, Sara DurlandBusbice, Darlene S. Barnard, Mark S. Marshall, Aimee K. Bence, Richard P. Beckmann. LY2606368, a second generation Chk1 inhibitor, inhibits growth of ovarian carcinoma xenografts either as monotherapy or in combination with standard-of-care agents. Molecular cancer therapeutics 2011;10:Supplement 1; abstr A108.
28. Turner NC, Reis-Filho JS, Russell AM, et al. BRCA1 dysfunction in sporadic basal-like breast cancer. Oncogene 2007;26:2126-32.
29. Deeb KK, Michalowska AM, Yoon CY, et al. Identification of an integrated SV40 T/t-antigen cancer signature in aggressive human breast, prostate, and lung carcinomas with poor prognosis. Cancer research 2007;67:8065-80.
30. Neve RM, Chin K, Fridlyand J, et al. A collection of breast cancer cell lines for the study of functionally distinct cancer subtypes. Cancer Cell 2006;10:515-27.
31. Bennett CN, Tomlinson CC, Michalowski AM, et al. Cross-species genomic and functional analyses identify a combination therapy using a CHK1 inhibitor and a ribonucleotide reductase inhibitor to treat triple-negative breast cancer. Breast Cancer Res 2012;14:R109.
32. Smith TJ, Khatcheressian J, Lyman GH, et al. 2006 update of recommendations for the use of white blood cell growth factors: an evidence-based clinical practice guideline. Journal of clinical oncology : official journal of the American Society of Clinical Oncology 2006;24:3187-205.
33. Patch AM, Christie EL, Etemadmoghadam D, et al. Whole-genome characterization of chemoresistant ovarian cancer. Nature 2015;521:489-94.
34. Pujade-Lauraine E, Hilpert F, Weber B, et al. Bevacizumab combined with chemotherapy for platinum-resistant recurrent ovarian cancer: The AURELIA open-label randomized phase III

trial. Journal of clinical oncology : official journal of the American Society of Clinical Oncology 2014;32:1302-8.

35. Hong D, Infante J, Janku F, et al. Phase I Study of LY2606368, a Checkpoint Kinase 1 Inhibitor, in Patients With Advanced Cancer. Journal of clinical oncology : official journal of the American Society of Clinical Oncology 2016;34:1764-71.

36. Scher HI, Halabi S, Tannock I, et al. Design and End Points of Clinical Trials for Patients With Progressive Prostate Cancer and Castrate Levels of Testosterone: Recommendations of the Prostate Cancer Clinical Trials Working Group. Journal of Clinical Oncology 2008;26:1148-59.

37. Hsu S, Kim M, Hernandez L, et al. IKK-epsilon coordinates invasion and metastasis of ovarian cancer. Cancer research 2012;72:5494-504.

38. Pecot CV, Bischoff FZ, Mayer JA, et al. A novel platform for detection of CK+ and CK- CTCs. Cancer discovery 2011;1:580-6.

39. Eisenhauer EA, Therasse P, Bogaerts J, et al. New response evaluation criteria in solid tumours: revised RECIST guideline (version 1.1). Eur J Cancer 2009;45:228-47.

40. Bubley GJ, Carducci M, Dahut W, et al. Eligibility and response guidelines for phase II clinical trials in androgen-independent prostate cancer: recommendations from the Prostate-Specific Antigen Working Group. Journal of clinical oncology : official journal of the American Society of Clinical Oncology 1999;17:3461-7.

41. Sonpavde G, Fleming MT, Hutson TE, Galsky MD. Trial design for metastatic castration-resistant prostate cancer. Journal of clinical oncology : official journal of the American Society of Clinical Oncology 2008;26:3647-8; author reply 8-9.

42. Vergote I, Rustin GJ, Eisenhauer EA, et al. Re: new guidelines to evaluate the response to treatment in solid tumors [ovarian cancer]. Gynecologic Cancer Intergroup. Journal of the National Cancer Institute 2000;92:1534-5.

43. Rustin GJ, Quinn M, Thigpen T, et al. Re: New guidelines to evaluate the response to treatment in solid tumors (ovarian cancer). Journal of the National Cancer Institute 2004;96:487-8.

16 APPENDICES

16.1 Appendix A – Performance Status Criteria

ECOG Performance Status Scale		Karnofsky Performance Scale	
Grade	Descriptions	Percent	Description
0	Normal activity. Fully active, able to carry on all pre-disease performance without restriction.	100	Normal, no complaints, no evidence of disease.
		90	Able to carry on normal activity; minor signs or symptoms of disease.
1	Symptoms, but ambulatory. Restricted in physically strenuous activity, but ambulatory and able to carry out work of a light or sedentary nature (e.g., light housework, office work).	80	Normal activity with effort; some signs or symptoms of disease.
		70	Cares for self, unable to carry on normal activity or to do active work.
2	In bed <50% of the time. Ambulatory and capable of all self-care, but unable to carry out any work activities. Up and about more than 50% of waking hours.	60	Requires occasional assistance, but is able to care for most of his/her needs.
		50	Requires considerable assistance and frequent medical care.
3	In bed >50% of the time. Capable of only limited self-care, confined to bed or chair more than 50% of waking hours.	40	Disabled, requires special care and assistance.
		30	Severely disabled, hospitalization indicated. Death not imminent.
4	100% bedridden. Completely disabled. Cannot carry on any self-care. Totally confined to bed or chair.	20	Very sick, hospitalization indicated. Death not imminent.
		10	Moribund, fatal processes progressing rapidly.
5	Dead.	0	Dead.

16.2 APPENDIX B – DRUGS KNOWN TO CAUSE QT PROLONGATION AND/OR INDUCE TORSADES DE POINTES

Generic Name	Brand Names	Drug Class	Therapeutic Use
Amiodarone	Cordarone®, Pacerone®, Nexterone®	Anti-arrhythmic	Abnormal heart rhythm
Anagrelide	Agrylin®, Xagrid®	Phosphodiesterase 3 inhibitor	Thrombocythemia
Arsenic trioxide	Trisenox®	Anti-cancer	Leukemia
Azithromycin	Zithromax®, Zmax®	Antibiotic	Bacterial infection
Chloroquine	Aralen®	Anti-malarial	Malaria infection
Chlorpromazine	Thorazine®, Largactil®, Megaphen®	Anti-psychotic / Anti-emetic	Schizophrenia/ nausea
Citalopram	Celexa®, Cipramil®	Anti-depressant, SSRI	Depression
Clarithromycin	Biaxin®, Prevpac®	Antibiotic	Bacterial infection
Cocaine	Cocaine	Local anesthetic	Topical anesthetic
Disopyramide	Norpace®	Anti-arrhythmic	Abnormal heart rhythm
Dofetilide	Tikosyn®	Anti-arrhythmic	Abnormal heart rhythm
Dronedarone	Multaq®	Anti-arrhythmic	Atrial Fibrillation
Droperidol	Inapsine®, Droleptan®, Dridol®, Xomolix®	Anti-psychotic / Anti-emetic	Anesthesia adjunct, nausea
Erythromycin	EES®, MY-E®, Robimycin®, EMycin®, Erymax®, Ery-Tab®, Eryc Ranbaxy®, Erypar®, Eryped®, Erythrocin Stearate Filmtab®, Erythrocot®, E-Base®, Erythroped®, Ilosone®, Pediamycin®, Zineryt®, Abboticin®, Abboticin-ES®, Erycin®, PCE Dispertab®, Stiemycine®, Acnasol®, Tiloryth®	Antibiotic	Bacterial infection; increase GI motility
Escitalopram	Cipralex®, Lexapro®, Nexito®, Seroplex®, Elicea®, Lexamil®, Lexam®, [Anxiset-E®, Exodus®, Esto®, Entact®, Losita®, Reposil®, Animaxen®, Esitalo®, Lexamil® (Not available in the US)]	Anti-depressant, SSRI	Major depression/ Anxiety disorders
Generic Name	Brand Names	Drug Class	Therapeutic Use
Flecainide	Tambocor®, Almarytm®, Apocard®, Ecrinal®, Flécaine®	Anti-arrhythmic	Abnormal heart rhythm
Halofantrine	Halfan®	Anti-malarial	Malaria infection

Haloperidol	Aloperidin®, Bioperidolo®, Brotopon®, Dozic®, Einalon S®, Eukystol®, Halosten®, Keselan®, Linton®, Peluces®, Serenace®, Serenase®, Sigaperidol®, Haldol® (US & UK), Duraperidol® (Germany),	Anti-psychotic	Schizophrenia, agitation
Ibutilide	Corvert®	Anti-arrhythmic	Abnormal heart rhythm
Methadone	Dolophine®, Symoron®, Amidone®, Methadose®, Physeptone®, Heptadon®	Opiate	Pain control, narcotic dependence
Moxifloxacin	Avelox®, Avalox®, Avelon®	Antibiotic	Bacterial infection
Ondansetron	Zofran®, Anset®, Ondemet®, Zuplenz®, Emetron®, Ondavell®, Emeset®, Ondisolv®, Setronax®	Anti-emetic	Nausea, vomiting
Pentamidine	NebuPent®, Pentam®	Antibiotic	Pneumocystis pneumonia
Pimozide	Orap®	Anti-psychotic	Tourette's tics
Procainamide (Oral off US mkt)	Pronestyl®, Procan®	Anti-arrhythmic	Abnormal heart rhythm
Quinidine	Quinaglute®, Duraquin®, Quinact®, Quinidex®, Cin-Quin®, Quinora®	Anti-arrhythmic	Abnormal heart rhythm
Sevoflurane	Ulane®, Sojourn®	Anesthetic, general	Anesthesia
Sotalol	Betapace®, Sotalex®, Sotacor®	Anti-arrhythmic	Abnormal heart rhythm
Thioridazine	Mellaril®, Novoridazine®, Thioril®	Anti-psychotic	Schizophrenia
Vandetanib	Caprelsa®	Anti-cancer	Thyroid cancer

Source: www.QTdrugs.org Updated September 2013.

16.3 APPENDIX C – LABORATORY STANDARD OPERATING PROCEDURE- TISSUE CORE COLLECTION

Lee Laboratory Standard Operating Procedure for Tissue Core Collection-
Needle Biopsy – Cryopreservation in OCT

Principle:
Core needle biopsies are used to sample tissue from a specific, defined location. These biopsies may consist of normal, pre-malignant and malignant tissue due to the multi-level tissue sample that is obtained. This type of sample is ideal for studying the micro-tumor environment.
Rapid freezing of the sample is required to prevent degradation of the proteins or RNA. Optimal Cutting Temperature (OCT) compound is an alcohol polymer that is liquid at room temperature and a solid at –20oC. This polymer is used to cryo-protect the tissue and provide a medium for cryo-sectioning.

Materials:
Cryomolds (Sakura Finetek Ca.t # 4728)
OCT (Sakura Finetek Cat. # 4583)
Dry ice
Ultra cold freezer (-700 to -800C)
Needle: 16 or 18 gauge
Permanent marker
Sterile forceps
Sterile Glass slides
Aluminum foil or 50ml Falcon tubes

Procedure:
1. Prepare all supplies prior to the biopsy procedure to avoid delay once the specimen has been obtained.
2. Label the handle and the front surface of the cryomold with the sample or participant's identifying information.
3. Perform core needle biopsy.
4. Pick the core from the biopsy needle onto a sterile glass slide.
5. Fill cryomold about 1/3 full with OCT. Place the cryomold in dry ice to partially freeze the OCT. The OCT should be jelly-like, not completely frozen.
6. Carefully lift the core biopsy by both ends with sterile forceps. **Do not stretch the biopsy or it will break.**
7. Lay the biopsy as straight as possible in the OCT. Once the sample touches the OCT, you

cannot reposition it or the sample will break apart.
8. Quickly add OCT on top of the biopsy, completely covering the sample.
9. Ensure the sample is level and freeze immediately in dry ice.
10. Store wrapped in aluminum foil or in a 50ml Falcon tube at –70ºC.

Note:
Do not lay the biopsy on frozen OCT and cover it with liquid OCT. The OCT will not fuse and will split into two sections when cutting the frozen tissue sections.

Frozen Section Slides
1. Frozen sections for proteomic analysis should be cut at 5-8um on plain, uncoated glass microscope slides.
2. The tissue section should be placed as close as possible to the center of the slide. Do not place the frozen section at the end of the slide.
3. Two tissue sections from the same biopsy may be placed on the same glass slide if space permits.
4. Do not allow the tissue section to air on the slide. Freeze immediately on dry ice or at –80ºC.

16.4 Appendix D – Hereditary Breast/Ovarian Cancer Syndrome Family History Criteria (modified from NCCN, 2013)

Personal history of **breast cancer** AND one or more of the following:

- A family member with a known deleterious *BRCA1* or *BRCA2* mutation.
- Diagnosed ≤50 with ≥ 1 first, second, or third degree relative with invasive breast cancer and/or ductal carcinoma in situ diagnosed at any age.
- Diagnosed at any age with ≥ 1 first, second, or third degree relative with invasive breast cancer and/or ductal carcinoma in situ ≤50.
- Diagnosed at any age with ≥ 2 first, second, or third degree relative with invasive breast cancer and/or ductal carcinoma in situ diagnosed at any age.
- Diagnosed at any age with ≥ 1 first, second, or third degree relative with epithelial ovarian cancer diagnosed at any age.
- Diagnosed at any age with ≥ 2 first, second, or third degree relative with pancreatic cancer and/or aggressive prostate cancer (Gleason score ≥7) diagnosed at any age.
- ≥ 1 first, second, or third degree MALE relative with invasive breast cancer and/or ductal carcinoma in situ diagnosed at any age.
- Individual ethnicity associated with higher mutation frequency (i.e. Ashkenazi Jewish).

Personal history of **ovarian cancer** AND one or more of the following:

- A family member with a known deleterious *BRCA1* or *BRCA2* mutation.
- ≥ 1 first, second, or third degree relative with invasive breast cancer and/or ductal carcinoma in situ diagnosed at any age.
- ≥ 2 first, second, or third degree relative with pancreatic cancer and/or aggressive prostate cancer (Gleason score ≥7) diagnosed at any age.
- ≥ 1 first, second, or third degree MALE relative with invasive breast cancer and/or ductal carcinoma in situ diagnosed at any age.
- Individual ethnicity associated with higher mutation frequency (i.e. Ashkenazi Jewish).

Reference:

NCCN, (2013). Genetic/familial high-risk assessment: Breast and ovarian. Version 4.2013. NCCN.org
