# Supplementary material for: The CHK1 inhibitor prexasertib in BRCA wild-type platinum-resistant recurrent high-grade serous ovarian carcinoma: a phase 2 trial
Source: Nat Commun. 2024 Mar 30;15:2805. doi: 10.1038/s41467-024-47215-6 (PMC10981752; doi:10.1038/s41467-024-47215-6)
Supplement: Supplementary file 3 — Description of Additional Supplementary Files [file 41467_2024_47215_MOESM3_ESM.pdf]

Description of Additional Supplementary Files

Supplementary Data 1. Summary of translational analyses performed on evaluable patient included in the study.

Supplementary Data 2. Treatment-related adverse events (TRAEs; n=49).

Supplementary Data 3. BROCA-GOv1 panel: gene list.

Supplementary Data 4. DNAseq analysis (BROCA-GOv1 panel).

Supplementary Data 5. Transcriptomic profiling of each patient.

Supplementary Data 6. Enriched KEGG pathways in clinical benefit (CB) and no clinical benefit (NCB) groups.

Supplementary Data 7. Differential gene expression of clinical benefit (CB) vs. no clinical benefit (NCB).

Supplementary Data 8. Sequences of siRNAs used in this study.
