# Supplementary material for: The CHK1 inhibitor prexasertib in BRCA wild-type platinum-resistant recurrent high-grade serous ovarian carcinoma: a phase 2 trial
Source: Nat Commun. 2024 Mar 30;15:2805. doi: 10.1038/s41467-024-47215-6 (PMC10981752; doi:10.1038/s41467-024-47215-6)
Supplement: Supplementary file 12 — Reporting Summary [file 41467_2024_47215_MOESM12_ESM.pdf]

nature portfolio

Corresponding author(s): Jung-Min Lee

Last updated by author(s): Mar 15, 2024

Reporting Summary

Nature Portfolio wishes to improve the reproducibility of the work that we publish. This form provides structure for consistency and transparency in reporting. For further information on Nature Portfolio policies, see our Editorial Policies and the Editorial Policy Checklist.

Statistics

For all statistical analyses, confirm that the following items are present in the figure legend, table legend, main text, or Methods section.

n/a	Confirmed	
☐	☒	The exact sample size (*n*) for each experimental group/condition, given as a discrete number and unit of measurement
☐	☒	A statement on whether measurements were taken from distinct samples or whether the same sample was measured repeatedly
☐	☒	The statistical test(s) used AND whether they are one- or two-sided<br>*Only common tests should be described solely by name; describe more complex techniques in the Methods section.*
☒	☐	A description of all covariates tested
☐	☒	A description of any assumptions or corrections, such as tests of normality and adjustment for multiple comparisons
☐	☒	A full description of the statistical parameters including central tendency (e.g. means) or other basic estimates (e.g. regression coefficient) AND variation (e.g. standard deviation) or associated estimates of uncertainty (e.g. confidence intervals)
☐	☒	For null hypothesis testing, the test statistic (e.g. *F*, *t*, *r*) with confidence intervals, effect sizes, degrees of freedom and *P* value noted<br>*Give P values as exact values whenever suitable.*
☒	☐	For Bayesian analysis, information on the choice of priors and Markov chain Monte Carlo settings
☒	☐	For hierarchical and complex designs, identification of the appropriate level for tests and full reporting of outcomes
☐	☒	Estimates of effect sizes (e.g. Cohen's *d*, Pearson's *r*), indicating how they were calculated

Our web collection on statistics for biologists contains articles on many of the points above.

Software and code

Policy information about availability of computer code

Data collection	No open source or commercial code was used for the data collection in this manuscript.
Data analysis	Software used: GraphPad Prism v9 and Microsoft Excel for statistical testing and visualization, DRAGEN v3.9.5 and Sequenza v3.0.0 for somatic variants, variant calls and CN files were input into R/Bioconductor (v4.2.2) using the maftools (v.2.14.0) package for analysis and visualization, GSEA (v4.3.2) softward for gene set enrichment analysis, FlowJo software v.10.6.1 for immune cell subset analysis, Gen5™ software for microplate data collection and analysis, Odyssey™ Fc gel documentation system for blot visualization, ImageStudio™ software for densitometric analysis, Clopper-Pearson method with R Studio, Version2022.12.0+353 for response measurements, Median Kaplan-Meier method with R Studio, Version2022.12.0+353 for progression-free survival analysis.

For manuscripts utilizing custom algorithms or software that are central to the research but not yet described in published literature, software must be made available to editors and reviewers. We strongly encourage code deposition in a community repository (e.g. GitHub). See the Nature Portfolio guidelines for submitting code & software for further information.

Data

Policy information about availability of data

All manuscripts must include a data availability statement. This statement should provide the following information, where applicable:

- Accession codes, unique identifiers, or web links for publicly available datasets
- A description of any restrictions on data availability
- For clinical datasets or third party data, please ensure that the statement adheres to our policy

The DNA sequencing data of BROCA-GOv1 generated in this study have been deposited in the BioProject database under accession code PRJNA1087413 [https://www.ncbi.nlm.nih.gov/bioproject/1087413]. The WES data generated in this study have been deposited in the dbGaP database under accession code phs003588.v1.p1 [http://www.ncbi.nlm.nih.gov/projects/gap/cgi-bin/study.cgi?study_id=phs003588.v1.p1]. The RNAseq data generated in this study have been deposited in the GEO database under accession code GSE249587 [https://www.ncbi.nlm.nih.gov/geo/query/acc.cgi?acc=GSE249587]. The processed RNAseq data are available at Supplementary Data 5. The raw clinical data are protected and are not available due to data privacy laws. Specific requests for access to de-identified clinical data should be sent to the corresponding author. The study protocol is available as a Supplementary Note in the Supplementary Information. The publicly available data of mRNA alteration of POLE and POLA1 used in this study are available in the cBioPortal database [https://www.cbioportal.org82-84]. The publicly available data of mRNA alteration of POLE and PFS in HGSOC used in this study are available in the Kaplan-Meier Plotter [ovarian cancer] database [http://www.kmplot.com85]. Source data are provided with this paper. The remaining data are available within the Article, Supplementary Information or Source Data file.

Research involving human participants, their data, or biological material

Policy information about studies with human participants or human data. See also policy information about sex, gender (identity/presentation), and sexual orientation and race, ethnicity and racism.

Reporting on sex and gender	Sex and gender were not considered as covariates for the study design as all patients are female. The study was not designed to capture sex or gender based effects. Sex of participants was determined by self-report.
Reporting on race, ethnicity, or other socially relevant groupings	N/A
Population characteristics	Patient and disease characteristics at baseline is provided in Supplementary Table 1.
Recruitment	Patients were recruited to and enrolled at National Cancer Institute, USA. Trial information was posted to the general public on the institutional webpage as well as governmental website (clinicaltrials.gov). The trial was publicized by presentations at regional and national conferences.<br><br>A potential selection bias may be represented by the distance traveled from a patient's home to the treatment center as it has been demonstrated to be an important prognostic variable for phase II clinical trials. If the study enrolled only distant patients, the overall results would have been impressively positive. Conversely, enrollment restricted to local patients may lead discouraging results. However, the relatively small number of patients included in the study makes hard to adjust adequately for known and unknown variables.
Ethics oversight	The trial was conducted according to federal law and good clinical practice regulations and was approved by the Institutional Review Board of the Center for Cancer Research, National Cancer Institute, USA.

Note that full information on the approval of the study protocol must also be provided in the manuscript.

Field-specific reporting

Please select the one below that is the best fit for your research. If you are not sure, read the appropriate sections before making your selection.

☒ Life sciences ☐ Behavioural & social sciences ☐ Ecological, evolutionary & environmental sciences

For a reference copy of the document with all sections, see nature.com/documents/nr-reporting-summary-flat.pdf

Life sciences study design

All studies must disclose on these points even when the disclosure is negative.

Sample size	For cohort 5, the sample size of 36 patients was selected to rule out an ORR of 20% in favor of ORR of 45%, using a two-tailed α=0.05 for an 89% power. The sample size of 35 patients for cohort 6 was chosen to rule out ORR of 15% in favor of ORR of 40%, using two-tailed α=0.05 for an 88% power. The regimen would be considered sufficient for the next stage of clinical development if ≥ 13/36 patients had a CR or PR in cohort 5, with the exact two-sided 95% CI ranging 20.8-53.8%, surpassing the minimum 20% ORR and containing the target 45% ORR. Conversely, the data would be sufficient if ≥ 11/35 patients achieved CR or PR in cohort 6, with the exact two-sided 95% CI ranging 16.9-49.3%, surpassing the minimum 15% ORR and containing the target 40% ORR. The accrual would be stopped if no one in the first 10 enrolled patients had CR or PR in either group, as the upper bound on a one-sided 90% CI of 1/10 patients would have been 33.7%. There was a high probability that the true ORR could be less than 33%, which was obtained in a previous cohort of recurrent BRCAwt HGSOC. Because cohorts 5 and 6 were closed early for enrollment, the participants from the cohorts 5 and 6 were reported together as a combined dataset to have a reasonable number of data analysis given the results are sufficiently similar between the two cohorts.

Data exclusions	No data was excluded.
Replication	Reproducibility of results were confirmed by repeating the experiments independently. The precise number of repeats is provided in the Methods section or in the Figure legends.
Randomization	This is a non-randomized, multi-cohort, single-arm phase II study, so patients were not randomly assigned into different groups but patients were allocated in each cohort according to the possibility of safely performing pre-treatment fresh biopsy (cohort 5: BRCA wild-type, platinum-resistant high grade serous ovarian cancer patients with biopsable disease, cohort 6: BRCA wild-type, platinum-resistant high grade serous ovarian cancer patients without biopsable disease).<br><br>For in vitro experiments, cells were randomly allocated into control and experimental groups.
Blinding	Investigators were blinded during data collection and analysis.

Reporting for specific materials, systems and methods

We require information from authors about some types of materials, experimental systems and methods used in many studies. Here, indicate whether each material, system or method listed is relevant to your study. If you are not sure if a list item applies to your research, read the appropriate section before selecting a response.

Materials & experimental systems

n/a	Involved in the study	
☐	☒	Antibodies
☐	☒	Eukaryotic cell lines
☒	☐	Palaeontology and archaeology
☒	☐	Animals and other organisms
☐	☒	Clinical data
☒	☐	Dual use research of concern
☒	☐	Plants

Methods

n/a	Involved in the study	
☒	☐	ChIP-seq
☐	☒	Flow cytometry
☒	☐	MRI-based neuroimaging

Antibodies

Antibodies used

CHK1 (#2360, 1:2000), Cell Signaling Technology
CHK1-S345 (#2348, 1:2000), Cell Signaling Technology
CHK1-S296 (#2349, 1:2000), Cell Signaling Technology
pKAP1-S824 (#4127, 1:2000), Cell Signaling Technology
KAP1 (#5868, 1:2000), Cell Signaling Technology
beta-actin (#3700, 1:2000), Cell Signaling Technology
Anti-mouse IgG, HRP-linked Antibody (#7076, 1:3000), Cell Signaling Technology
Anti-rabbit IgG, HRP-linked Antibody (#7074, 1:3000), Cell Signaling Technology
POLA1 (#Ab31777, 1:2000), Abcam
POLE (#PA5-78113, 1:2000), Thermofisher Scientific
CD11b (#301318, 1:50), BioLegend
CD11c (#337218, 1:50), BioLegend
CD14 (#325622, 1:50), BioLegend
CD141 (#344114, 1:50), BioLegend
CD15 (#323040, 1:100), BioLegend
CD16 (#302018, 1:50), BioLegend
CD19 (#302220, 1:50), BioLegend
CD197 (#353204, 1:50), BioLegend
CD1c (#331522, 1:50), BioLegend
CD25 (#302612, 1:50), BioLegend
CD27 (#124222, 1:100), BioLegend
CD279 (PD1) (#329920, 1:50), BioLegend
CD28 (#302966, 1:50), BioLegend
CD3 (#317312, 1:50), BioLegend
CD303 (#354210, 1:50), BioLegend
CD33 (#303404, 1:50), BioLegend
CD366 (TIM-3) (#345008, 1:50), BioLegend
CD4 (#357408 and #357410, 1:50), BioLegend
CD45RA (#304122, 1:50), BioLegend
CD56 (#304612, 1:50), BioLegend
CD8 (#344714 and #344748, 1:50), BioLegend
CD83 (#305308, 1:50), BioLegend

CTLA-4 (#349906, 1:50), BioLegend
Foxp3 (#320112, 1:50), BioLegend
GITR (#371204, 1:50), BioLegend
HLA-DR (#307620 and #307616, 1:50), BioLegend
ICOS (#313518, 1:50), BioLegend
Ki-67 (#350510, 1:50), BioLegend
PE-conjugated anti-EpCAM (#130-091-253, 1:200), Miltenyi Biotec
CD45 (#304014, 1:50), BioLegend
CD117 (#313212, 1:100), BioLegend
CXCR4 (#306516, 1:100), BioLegend
PDL1 (#329708, 1:100), BioLegend
MUC-1 (#559774, 1:50), BD Biosciences

Validation

All the commercial antibodies undergo extensive validation testing tailored to specific applications by manufacturers provided above. This includes analyzing a diverse range of cell lines with known target expression levels, verifying correct subcellular localization or treatment-induced translocation, and comparing results with both the antibody and an isotype control to ensure an acceptable signal-to-background ratio. Additionally, the target-specific signal is confirmed in transfected cells, knockout cells, or cells treated with siRNA.

Eukaryotic cell lines

Policy information about cell lines and Sex and Gender in Research

Cell line source(s)

OVCAR3 and OVCAR5 (BRCAwt, platinum-resistant HGSOC cell lines) were obtained from NCI-60 collection at the NCI, Frederick, MD, USA. The CHK1i resistant OVCAR5-PrexR cell line was developed from parental OVCAR5 as described earlier (ref 45 in the manuscript). OVCAR3-PrexR cell line was a gift from Dr. Michail Shipitsin, Acrivon therapeutics Inc., based on an MTA between Acrivon and NCI.

Authentication

All cell lines authentication was evaluated by Short Tandem Repeat analysis conducted by Labcorp (NC, USA).

Mycoplasma contamination

All cell lines applied in this study were tested negative for mycoplasma using MycoAlert (#NC9719283, Lonza).

Commonly misidentified lines
(See ICLAC register)

No cell lines used in this paper are listed in the database of commonly misidentified cell lines.

Clinical data

Policy information about clinical studies

All manuscripts should comply with the ICMJEguidelines for publication of clinical research and a completedCONSORT checklist must be included with all submissions.

Clinical trial registration

NCT02203513

Study protocol

The full study protocol can be found in ClinicalTrials.gov (https://classic.clinicaltrials.gov/ct2/show/NCT02203513)

Data collection

Between January 25, 2017, and March 23, 2020, 49 BRCAwt, platinum-resistant recurrent HGSOC patients were enrolled, including 24 patients with biopsiable disease (cohort 5) and 25 patients without safely biopsiable disease (cohort 6).Data were collected by the study staff at the Women’s Malignancies Branch, Center for Cancer Research, National Cancer Institute, National Institutes of Health, Bethesda, Maryland, 20892, USA.

Outcomes

The primary endpoint was ORR as assessed by Investigators according to RECISTv1.1. Secondary endpoints included safety and PFS. The evaluation of pharmacodynamic and predictive biomarkers of CHK1i response or resistance was an exploratory endpoint.

All 49 patients received at least one dose of prexasertib. Ten patients were not assessable for tumor response per Response Evaluation Criteria In Solid Tumors (RECIST) 1.1 criteria because of no restaging CT scans after 2 cycles of treatment due to withdrawal of consent or intercurrent illness during cycle 1 (Fig. 2a-b). Among the RECIST-evaluable patients (n=39), objective response rate (ORR) was 30.8% (12/39, 95% confidence interval [CI] 17-47.6), with 33.3% in cohort 5 (6/18) and 28.6% in cohort 6 (6/21), respectively. Disease control rate (DCR), defined by the sum of patients with partial response (PR) and stable disease (SD) ≥ 6 months, was 56.4% (22/39), with 44.4% (8/18) in cohort 5 and 66.7% (14/21) in cohort 6. The median progression-free survival (PFS) was 5 months, with 4 months in cohort 5 (n=18) and 6 months in cohort 6 (n=21) (Fig. 2c-d and Supplementary Data 1). In the intention-to-treat (ITT) population (n=49), an ORR of 24.5% (12/49) and a DCR of 44.9% (22/49) were observed. These results were similar to our previous report in which ORR was 31.6% (6/19) in the platinum-resistant HGSOC, and 28.6% (8/28) in ITT population (ref 29 in the manuscript), confirming the therapeutic potential of CHK1i in this population. Of 12 who had PR per RECIST criteria, median duration of response (DoR) was 5 months (95% CI 3-11), with 6.5 months in cohort 5 (n=6) and 4.5 months in cohort 6 (n=6), respectively.

Any grade treatment-related adverse events (TRAEs) were listed in Supplementary Data 2. The most common (in >10% patients) grade 3 or 4 TRAEs were hematological toxicities, such as neutropenia (42/49, 85.7%), leukocytopenia (38/49, 77.6%), lymphocytopenia (23/49, 46.9%), thrombocytopenia (20/49, 40.8%), anemia (15/49, 30.6%) and febrile neutropenia (6/49, 12.2%), consistent with previous reports (ref 29 in the manuscript). Of note, granulocyte colony-stimulating factors were given after checking the nadir on cycle 1 day 8 to avoid treatment delay or dose reduction.

Flow Cytometry

Plots

Confirm that:

- [x] The axis labels state the marker and fluorochrome used (e.g. CD4-FITC).
- [x] The axis scales are clearly visible. Include numbers along axes only for bottom left plot of group (a 'group' is an analysis of identical markers).
- [x] All plots are contour plots with outliers or pseudocolor plots.
- [x] A numerical value for number of cells or percentage (with statistics) is provided.

Methodology

Sample preparation	Circulating tumor cells (CTCs) analysis<br>Peripheral blood samples (8-mL EDTA tubes) were collected at baseline, and at C1D15. After RBC lysis, blood cells were incubated with nuclear dye (#H3570, Hoechst 33342, Life Technologies, DC, USA), viability dye (#L34966, LIVE/DEAD Fixable Aqua, Life Technologies) and antibodies including PE-conjugated anti-human epithelial cell adhesion molecule (EpCAM) Ab (#130-091-253, clone HEA-125, Miltenyi Biotec, CA, USA).<br><br>Immune cell subset analysis<br>Peripheral blood specimens (two-8ml BD Vacutainer CPT tubes) were collected at baseline, and at C1D15. Peripheral blood mononuclear cells (PBMCs) were obtained using centrifugation and viably frozen until analysis. PBMCs were incubated with Fc receptor blocking reagent (Miltenyi Biotec) and stained with monoclonal antibodies (20 minutes at 4°C).
Instrument	Multiparametric flow cytometry (MACSQuant; Miltenyi Biotec)
Software	FlowJo software v.10.6.1
Cell population abundance	Dead cells were excluded from the analysis using the viability dye, LIVE/DEAD Fixable Aqua. Purity above 90% was accepted for downstream applications.
Gating strategy	All cells were gated on forward and side scatter parameters (FSC/SSC), then dead cells were excluded.<br><br>CTCs: The anti-PE magnetic beads (#130-048-801, Miltenyi Biotec) were used to enrich EpCAM-positive cells. Viable, nucleated, EpCAM-positive, CD45 (#304014, clone HI30, BioLegend, CA, USA) negative cells were considered CTCs and further characterized for CD117 (#313212, clone 104D2, BioLegend), CXCR4 (#306516, clone 12G5, BioLegend), PDL1 (#329708, clone 29E.2A3, BioLegend) and MUC-1 (#559774, clone HMPV, BD Biosciences, CA, USA) expression.<br><br>Immune cell subset: Cells were gated on specific immune cell subsets (Supplementary Fig. 9-12), and further for functional markers (Supplementary Table 5).

- [x] Tick this box to confirm that a figure exemplifying the gating strategy is provided in the Supplementary Information.
